# Id1 Stabilizes Epiblast Identity by Sensing Delays in Nodal Activation and Adjusting the Timing of Differentiation

**DOI:** 10.1016/j.devcel.2019.05.032

**Published:** 2019-08-19

**Authors:** Mattias Malaguti, Rosa Portero Migueles, Guillaume Blin, Chia-Yi Lin, Sally Lowell

**Affiliations:** 1 MRC Centre for Regenerative Medicine, Institute for Stem Cell Research, School of Biological Sciences, the University of Edinburgh, 5 Little France Drive, Edinburgh EH16 4UU, UK

**Keywords:** pluripotent, id1, peri-implantation development, Nodal, FGF, Nanog, epiblast

## Abstract

Controlling responsiveness to prevailing signals is critical for robust transitions between cell states during development. For example, fibroblast growth factor (FGF) drives naive pluripotent cells into extraembryonic lineages before implantation but sustains pluripotency in primed cells of the post-implantation epiblast. Nanog supports pluripotency in naive cells, while Nodal supports pluripotency in primed cells, but the handover from Nanog to Nodal does not proceed seamlessly, opening up the risk of aberrant differentiation if FGF is activated before Nodal. Here, we report that Id1 acts as a sensor to detect delays in Nodal activation after the downregulation of Nanog. Id1 then suppresses FGF activity to delay differentiation. Accordingly, Id1 is not required for naive or primed pluripotency but rather stabilizes epiblast identity during the transition between these states. These findings help explain how development proceeds robustly in the face of imprecise signals and highlight the importance of mechanisms that stabilize cell identity during developmental transitions.

## Highlights

• Id1 acts as a “sensor” to detect when cells lack both Nanog and Nodal activity
• Id1 suppresses FGF in order to protect cells from aberrant differentiation
• Id1 protects pluripotency during transitions between naive and primed cell states
• Id1 is required for a robust transition through peri-implantation development.

## Graphical Abstract

**Figure:**
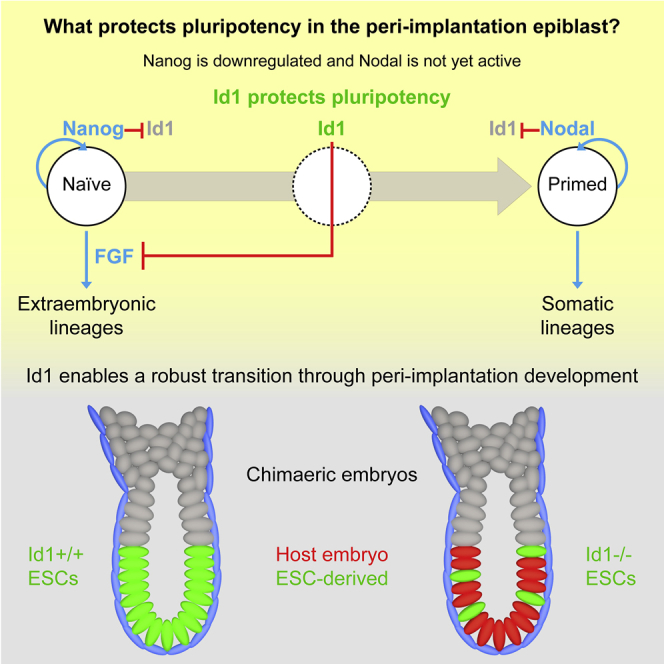

## Teaser

Signals are often re-purposed as development proceeds. For example, FGF drives extraembryonic differentiation of pluripotent cells before the embryo implants, yet maintains somatic potency in their post-implantation descendants. Malaguti et al. show how pluripotent cells sense changes in their environment to ensure that FGF does not become activated prematurely.

## Introduction

Pluripotent cells in the early embryo choose their fate according to the signals they receive from their local environment (Arnold and Robertson, 2009). However, pluripotent cells are unlikely to respond passively to prevailing signals. Rather, the ability to respond to or ignore particular signals must be tightly coordinated with changes in differentiation potential in order to ensure that cell fate decisions are not misdirected by premature fluctuations in pro-differentiation cues.

Control over signal responsiveness becomes particularly important where the same signal is re-deployed to regulate successive cell fate restrictions. For example, fibroblast growth factor (FGF) drives naive pluripotent cells in the early embryo to differentiate into extraembryonic cell types (Chazaud et al., 2006, Hamilton and Brickman, 2014, Nichols et al., 2009, Yamanaka et al., 2010), whereas FGF helps to sustain pluripotency once pluripotent cells have transitioned into a “primed” state (Arnold and Robertson, 2009, Brons et al., 2007, Tesar et al., 2007). Therefore, for this transition to proceed successfully, the shift in FGF activity must somehow be timed to occur only after cells irreversibly commit to the primed epiblast state.

Nodal protects pluripotency in the primed epiblast of the post-implantation embryo (Camus et al., 2006, Mesnard et al., 2006) while Nanog protects pluripotency in the naive epiblast of the pre-implantation embryo (Mitsui et al., 2003). The handover between these two factors does not, however, appear to proceed seamlessly: some Nanog-negative epiblast cells lack Nodal activity in the late pre-implantation embryo (Granier et al., 2011). With neither Nanog nor Nodal available to sustain epiblast identity, these transiting epiblast cells would be in a precarious state, unless some other factor comes into play to protect them against the pro-endoderm effects of autocrine FGF (Chazaud et al., 2006, Hamilton and Brickman, 2014, Nichols et al., 2009, Yamanaka et al., 2010). This putative factor should have three key properties: the ability to sense low levels of Nodal activity, the ability to dampen FGF responsiveness, and the ability to protect pluripotent cells from differentiation.

A likely candidate is the BMP (Bone Morphogenic Protein) target gene *Id1*. *Id1* is sensitive to Nodal activity (Galvin et al., 2010) and is able to prevent differentiation of pluripotent cells (Ying et al., 2003, Zhang et al., 2010), but the details of when and how it operates remain unclear. It has been proposed that Id1 supports naive pluripotency by maintaining high levels of Nanog (Galvin-Burgess et al., 2013, Romero-Lanman et al., 2012, Ying et al., 2003). However, surprisingly, we report here that Id1 protein is absent from the embryonic day (E) 3.5 embryo and is only expressed in cells that have lost Nanog expression during peri-implantation development. This seems incompatible with the idea that BMP-Id1 maintains naive pluripotency but is consistent with idea that Id1 comes into play to protect epiblast identity after downregulation of Nanog.

Here, we report that Id1 stabilizes an epiblast identity specifically during the transition between naive and primed states. Id1 acts as a “sensor” to detect when cells have lost Nanog expression but have not yet acquired Nodal activity. Id1 then suppresses FGF in order to protect these cells from aberrant differentiation. Once a Nodal-responsive post-implantation epiblast state has been achieved, Nodal suppresses Id1 expression and so permits FGF activity to rise to help sustain pluripotency in newly configured primed epiblast cells.

We propose that this mechanism helps to coordinate changes in extrinsic and intrinsic information to ensure a robust transition through peri-implantation development.

## Results

### Pluripotent Cells Remain Resistant to BMP Signaling until Peri-implantation Development

We examined whether pluripotent cells modulate responsiveness to prevailing signals as they proceed toward differentiation. We focused on BMP signaling because BMP suppresses differentiation of pluripotent cells in culture (Ying et al., 2003) and *in vivo* (Di-Gregorio et al., 2007). The BMP target gene *Id1* (Hollnagel et al., 1999) recapitulates the effects of BMP on pluripotent cells (Malaguti et al., 2013, Ying et al., 2003, Zhang et al., 2010) and provides a biologically relevant readout of BMP activity (Figures S1A–S1C).

**Figure 1:**
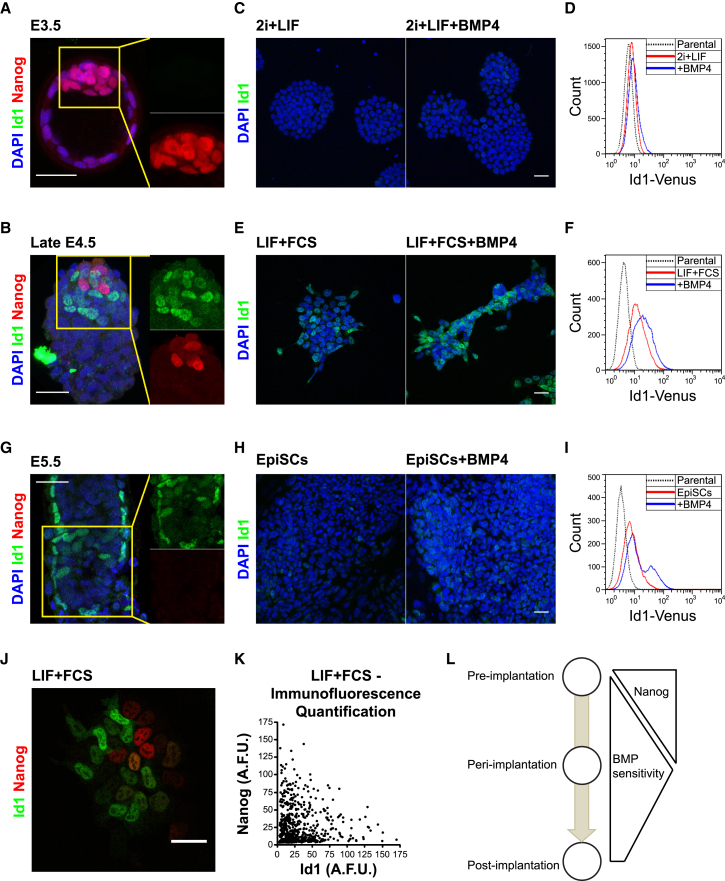
Pluripotent Cells Remain Resistant to BMP Signaling until Peri-implantation Stages of Development (A) Immunofluorescent staining of E3.5 blastocyst for Nanog and the BMP target Id1. (B) Immunofluorescent staining of late E4.5 blastocyst for Id1 and Nanog. (C) Immunofluorescent Id1 staining of ESCs cultured in 2i + LIF, unstimulated or stimulated with 10 ng/mL BMP4 for 48 h. (D) Flow cytometry analysis of Id1-Venus reporter ESCs cultured in 2i + LIF, unstimulated or stimulated with 10 ng/mL of BMP4 for 48 h. (E) Immunofluorescent Id1 staining of ESCs cultured in LIF + FCS, unstimulated or stimulated with 10 ng/mL of BMP4 for 48 h. (F) Flow cytometry analysis of Id1-Venus reporter ESCs cultured in LIF + FCS, unstimulated or stimulated with 10 ng/mL of BMP4 for 48 h. (G) Immunofluorescent staining of E5.5 embryo for Id1 and Nanog. (H) Immunofluorescent Id1 staining of EpiSCs, unstimulated or stimulated with 10 ng/mL of BMP4 for 48 h. (I) Flow cytometry analysis of Id1-Venus reporter EpiSCs, unstimulated or stimulated with 10 ng/mL of BMP4 for 48 h. (J) Immunofluorescent staining of ESCs cultured in LIF + FCS for Id1 and Nanog. (K) Quantification of Id1 and Nanog immunofluorescent signal in single ESCs cultured in LIF + FCS. (L) Diagram illustrating how BMP sensitivity increases around the time of implantation, as Nanog is being lost, and decreases following implantation. Scale bars, 30 μm. See also Figure S1.

*Bmp4*/*7* and pSmad1 are readily detectable in pre-implantation embryos at E3.5 (Coucouvanis and Martin, 1999, Graham et al., 2014). However, to our surprise, we were unable to detect the product of the direct BMP target gene Id1 in E3.5 embryos (Figure 1A) or in early E4.5 embryos (data not shown). We then examined embryos after E4.5, at the latest stage obtainable before the embryo implants. These embryos contain a subpopulation of Id1+ cells scattered throughout the epiblast in a salt-and-pepper distribution (Figure 1B). This suggests that patterning of Id1 is unlikely to be explained only by exposure to exogenous BMP ligands (because these ligands are diffusible and so unlikely to adopt a salt-and-pepper distribution) and instead might reflect cell-cell variability in BMP responsiveness.

In order to test this, we examined pluripotent cells in culture, where we could stimulate cells with BMP4. We first examined cells in 2i + LIF culture, which supports a stage of pluripotency equivalent to that of the early E4.5 blastocyst (Boroviak et al., 2015). We were unable to detect Id1 protein even after stimulating 2i + LIF cells with high doses (10 ng/mL) of BMP4 (Figure 1C). These findings were confirmed using cells in which an Id1-Venus fusion was expressed from the *Id1* locus (Figures 1D and S1D–S1G) (Malaguti et al., 2013, Nam and Benezra, 2009).

We then examined embryonic stem cells (ESCs) in LIF (leukemia inhibitory factor) + fetal calf serum (FCS), a culture condition that supports a mixture of naive and primed cells (Nichols and Smith, 2009). We could detect Id1 protein in some cells, although a subpopulation remained Id1 negative even when stimulated with BMP4 (Figures 1E and 1F), in keeping with reports that naive cells do not activate Id1 in response to BMP (Gomes Fernandes et al., 2016).

We then examined post-implantation embryos and epiblast stem cells (EpiSCs) (Brons et al., 2007, Tesar et al., 2007). As expected, the E5.5 epiblast expresses Id1 in cells close to the extraembryonic ectoderm, a source of BMP signals (Arnold and Robertson, 2009) (Figures 1G and S1H). EpiSC stimulated with BMP4 express moderate levels of Id1 in a minor subset of cells (Figures 1H and 1I), indicating that BMP responsiveness decreases as pluripotent cells reach a primed state. This transient window of Id1 expression at the onset of the transition between naive and primed pluripotency can be recapitulated *in vitro* in epiblast-like cell (EpiLC) differentiation (Figure S1J).

Some epiblast cells in the late E4.5 embryo downregulate the naive determinant Nanog to prepare for the transition to a primed state (Xenopoulos et al., 2015). We detect Id1 exclusively within these Nanog-low cells (Figure 1B). Similarly, Id1 is expressed predominantly in Nanog-low cells in LIF + FCS (Figures 1J and 1K).

We conclude that pluripotent cells modulate responsiveness to BMP4 over time. They become most responsive as they enter a transition phase between naive and primed pluripotency, corresponding to a stage of peri-implantation development after downregulation of Nanog but before establishment of a primed pluripotent state (Figure 1L).

### Id1 Predicts the Probability of Differentiating after Downregulation of Nanog

It is surprising that Id1, which maintains pluripotency of ESCs (Ying and Smith, 2003, Zhang et al., 2010), is not expressed in Nanog-high cells *in vivo* or in culture (Figures 1B, 1J, and 1K). Could it instead be protecting epiblast identity during the transition from naive to primed states?

The transition to a primed state is initiated by downregulation of Nanog in concert with other components of the naive gene regulatory network (GRN) (Kalkan et al., 2017). However, loss of Nanog does not commit cells to undergo this transition: some Nanog-low cells resist differentiation and revert back to a Nanog-high state (Chambers et al., 2007, Kalmar et al., 2009). We asked whether Id1 identifies those cells that resist differentiation after loss of Nanog.

**Figure 2:**
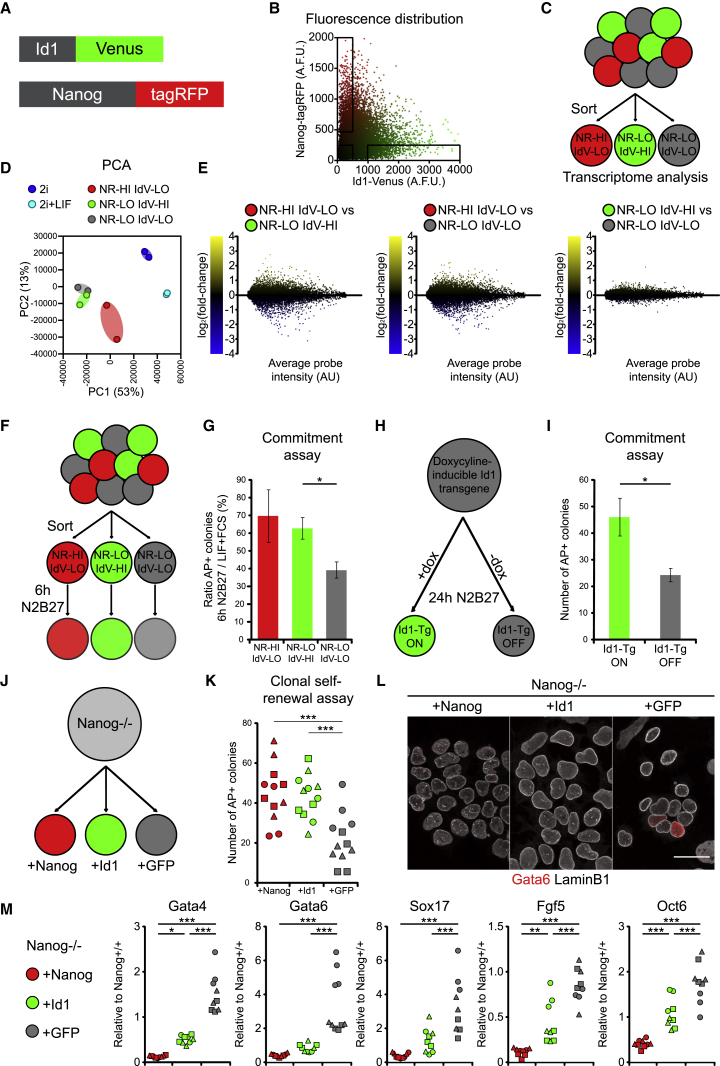
Id1 Protects Pluripotent Cells from Differentiation in the Absence of Nanog (A) Diagrammatic structure of Id1-Venus Nanog-tagRFP double reporter ESCs. (B) Flow cytometry of Id1-Venus Nanog-tagRFP cells cultured in LIF + FCS confirm that high levels of Id1 expression are observed predominantly in Nanog-low cells. Gates used for sorting experiments are displayed. (C) Sorting strategy for downstream transcriptome analysis of LIF + FCS cultures. (D) PCA of the sorted subpopulations, 2i and 2i + LIF cultures. (E) Pairwise transcriptomic comparisons of the three sorted subpopulations. (F) Experimental strategy. Id1-Venus Nanog-tagRFP ESCs cultured in LIF + FCS were sorted into three subpopulations then assayed for their ability to form AP+ colonies when plated at clonal density immediately after sorting and after 6-h N2B27 culture. (G) Proportion of cells capable of resisting differentiation after 6-h N2B27 culture. Number of AP+ colonies obtained after replating 6-h N2B27 cultures in LIF + FCS, divided by number of AP+ colonies obtained after replating cells in LIF + FCS immediately after sorting. Plating density: 1,000 cells/9 cm dish. Data are represented as mean ± SEM of three independent experiments. (H) Experimental strategy for Id1 gain-of-function experiment. ESCs carrying a doxycycline-inducible *Id1* transgene were transferred from LIF + FCS culture to N2B27 for 24 h, in the presence or absence of 1μg/mL doxycycline. The cells were assayed for their ability to form AP+ colonies when replated at clonal density in LIF + FCS. (I) Number of AP+ colonies obtained after replating cells as described in (H). Plating density: 100 cells/well of a 6-well plate. Data are represented as mean ± SEM of five independent experiments. (J) Diagram of rescue of Nanog^−⁄−^ cells. Clonal cell lines were generated to stably express *Nanog* (positive control), *Id1,* or *GFP* (negative control). (K) Number of undifferentiated AP+ colonies obtained upon plating Nanog-rescue cells in LIF + FCS. Each shape represents a different clonal line. Plating density: 100 cells/well of a 6-well plate. (L) Immunofluorescent staining of Nanog-rescue cells cultured in LIF + FCS for Gata6 and LaminB1. Scale bar, 30 μm. (M) qRT-PCR analysis of Nanog-rescue cells cultured in LIF + FCS. Each shape represents a different clonal line. Statistical analyses: for comparison of two samples: two-tailed unpaired Student’s t test; for comparison of three samples: one-way ANOVA followed by Tukey’s multiple comparison test. ^∗^p < 0.05, ^∗∗^p < 0.01, ^∗∗∗^p < 0.001. NR, Nanog-tagRFP; IdV, Id1-Venus; HI, high; LO, low; AP, alkaline phosphatase; dox, doxycycline; Tg, transgene. See also Figure S2 and Table S1.

We generated a dual-reporter ESC line, which expresses an Id1-Venus fusion protein from the endogenous *Id1* locus (Malaguti et al., 2013, Nam and Benezra, 2009), and a Nanog-tagRFP fusion protein from the endogenous *Nanog* locus (Figures 2A and S2A–S2E). We first confirmed that Nanog and Id1 tend to mark different subpopulations in LIF + FCS (Figure 2B). We then sorted three populations of cells from LIF + FCS: Nanog-high (NR-HI IdV-LO), Id1-high Nanog-low (IdV-HI NR-LO), and Id1-low Nanog-low cells (IdV-LO NR-LO) (Figures 2C and S2F).

As expected (Festuccia et al., 2012), transcriptomes differed between Nanog-high cells and Nanog-low cells. In contrast, within the Nanog-low compartment, transcriptomes of Id1-high and Id1-low cells were almost indistinguishable (Figures 2D and 2E; Table S1).

Id1 is not a transcriptional regulator: it acts by controlling the activity of a range of proteins (Norton, 2000, Roberts et al., 2001, Yates et al., 1999), so it seemed plausible that Id1-high cells may be more resistant to differentiation than Id1-low cells despite their similar transcriptomes.

We tested the sorted subpopulations for resistance to differentiation. We challenged cells with differentiation medium (N2B27) for 6 h and then returned them to self-renewal conditions at clonal density to assess how many cells remained undifferentiated. We also plated sorted cells directly into self-renewal conditions at clonal density to measure the number of undifferentiated cells in each starting population. We combined these data to establish the proportion of undifferentiated cells that resist differentiation during the 6 h challenge (Figure 2F).

This reveals that Id1-high cells resist differentiation more effectively than Id1-low cells: the majority (62% ± 6%) of IdV-HI NR-LO cells resisted differentiation, as did the majority (69% ± 15%) of NR-HI IdV-LO cells. Only a minority (39% ± 5%) of IdV-LO NR-LO cells were able to resist differentiation (Figure 2G).

Although there is low residual expression of Nanog and other naive pluripotency transcription factors within our “Nanog-low” sorted subpopulations (Figures S2F and S2G), this cannot explain our findings because there was no difference in expression of these factors between Id1-high and Id1-low cells (Figures S2G and S2H), nor was there any difference in the number of colony-forming cells prior to the differentiation challenge (Figure S2I), indicating that there are no functional differences in naive transcription factor activity between the two populations.

We conclude that Id1 identifies a subpopulation of Nanog-low cells that resist differentiation independently of the activity of the naive pluripotency GRN.

### Id1 Protects Pluripotent Cells from Differentiation in the Absence of Nanog

Having seen that Id1 correlates with resistance to differentiation after downregulation of Nanog, we asked whether Id1 is capable of suppressing differentiation after downregulation of Nanog. We made use of an ESC line containing a doxycycline-inducible *Id1* transgene (Malaguti et al., 2013). We placed these cells in N2B27 for 24 h, a time frame that is sufficient to downregulate members of the naive GRN (Kalkan et al., 2017), in the presence or absence of doxycycline. We then replated the cells clonally in self-renewal conditions (Figure 2H). We find that forced expression of Id1 during this time window in which the naive GRN is dismantled increases the number of cells that resist differentiation (Figure 2I).

If Id1 protects pluripotent cells from differentiation in the absence of Nanog, then it should be able to rescue the spontaneous differentiation phenotype of Nanog-null cells in LIF + FCS (Chambers et al., 2007). Forced expression of Id1 restores the colony-forming ability of Nanog-null cells to a similar extent to forced expression of Nanog itself (Figures 2J and 2K) and reduces the expression of markers of primitive endoderm (*Gata4*, *Gata6*, and *Sox17*) and primed epiblast (*Fgf5* and *Oct6*, also known as *Pou3f1*) (Figures 2L and 2M).

These data suggest that Id1 is responsible for protecting pluripotent cells from differentiation after downregulation of Nanog.

### A Coordinated Shift in BMP and FGF and Nodal Responsiveness after Downregulation of Nanog

We looked for transcriptional changes that might explain why IdV-LO NR-LO cells are more susceptible to differentiation than IdV-HI NR-LO cells.

**Figure 3:**
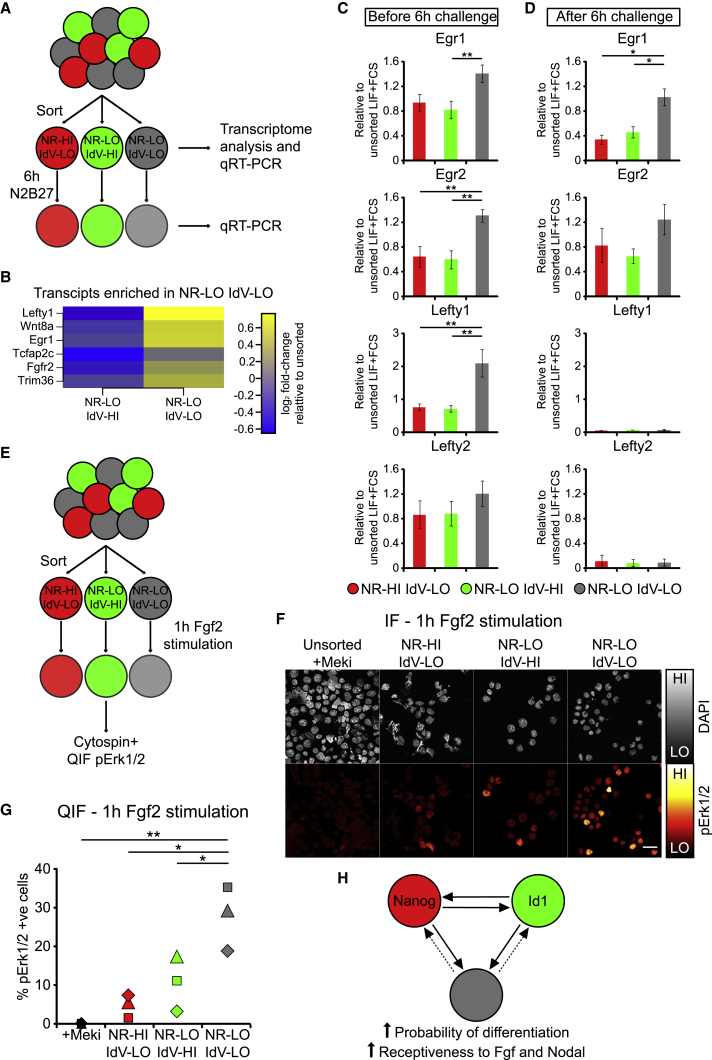
A Coordinated Shift in BMP, FGF, and Nodal Responsiveness after Downregulation of Nanog (A) Experimental strategy. ESCs cultured in LIF + FCS were sorted into three subpopulations based on Id1-Venus and Nanog-tagRFP. Samples were taken for gene expression analysis immediately after sorting and after 6 h culture in N2B27 differentiation medium. (B) Heatmap of transcripts significantly enriched in NR-LO IdV-LO relative to NR-LO IdV-HI subpopulations. Significance was defined as log_2_(fold-change) > 0.5, p value adjusted for multiple testing correction < 0.5. (C) qRT-PCR analysis of sorted subpopulations before the 6-h N2B27 differentiation challenge. Data are represented as mean ± SEM of seven independent experiments. (D) qRT-PCR analysis of sorted subpopulations after the 6-h N2B27 differentiation challenge. Data are represented as mean ± SEM of three independent experiments. (E) Experimental strategy for acute Fgf stimulation of sorted subpopulations. Following sorting, cells were cultured in N2B27+10 ng/mL Fgf2 in suspension, then cytospun and stained for pErk1/2 expression. (F) Immunofluorescent staining of sorted subpopulations for pErk1/2. Scale bar, 30 μm. (G) Percentage of pErk1/2-positive cells following 1 h Fgf2 stimulation of sorted subpopulations, calculated by quantitative immunofluorescence of cells shown in (F). Samples from the same sort are indicated with the same shape. (H) Model: once Nanog is lost from pluripotent cells, Id1 expression is associated with resistance to differentiation and lower expression of Fgf and Nodal signaling targets. Statistical analyses of qRT-PCR and QIF data were performed using a one-way ANOVA followed by Tukey’s multiple comparison test. ^∗^p < 0.05, ^∗∗^p < 0.01, ^∗∗∗^p < 0.001. Methods for statistical analysis of transcriptomic data are described in the STAR Methods section. NR, Nanog-tagRFP; IdV, Id1-Venus; HI, high; LO, low, Meki: Mek inhibitor (1 μM PD0325901); QIF, quantitative immunofluorescence; +ve, positive. See also Figure S3 and Table S1.

Compared with IdV-HI NR-LO cells, only six protein-coding genes were enriched in IdV-LO NR-LO cells (Figures 3A and 3B). Only two of these, *Egr1* and *Lefty1*, were confirmed by qRT-PCR to be differentially expressed (Figures 3C and S3A). *Egr1* and *Lefty1* are readouts of the FGF and Nodal signaling pathways, respectively, and these are the two pathways that sustain pluripotency in primed pluripotent cells (Brons et al., 2007, Camus et al., 2006, Tesar et al., 2007). We confirmed that the related FGF target gene *Egr2*, although not the related Nodal target gene *Lefty2*, is also enriched in IdV-LO NR-LO cells (Figure 3C). Id1 rescue of Nanog-null cells also correlates with reduced expression of *Egr1* (Figure S3B).

The FGF target gene *Egr1* (although not the Nodal targets genes *Lefty1* or *Lefty2*) remained enriched in Id1-low cells after a 6 h challenge with differentiation media, which is the time at which Id1-high cells display their relative resistance to differentiation (Figure 3D). Intriguingly, we do not observe differences in expression of naive or primed pluripotency markers between IdV-LO NR-LO and IdV-HI NR-LO cells at this time point (Figures S3C and S3D), suggesting that differences in FGF sensitivity and response can predict resistance to differentiation prior to overt changes in pluripotency marker expression.

Having observed an increase in FGF target gene expression in IdV-LO NR-LO cells, we next asked whether these cells are more responsive to acute stimulation with exogenous Fgf2 than Id1-high cells (Figure 3E). We find that a higher proportion of IdV-LO NR-LO cells respond to Fgf2 stimulation by phosphorylating Erk1/2 (a direct readout of FGF activity) than Id1-high or Nanog-high cells (Figures 3F and 3G).

These data suggest that there is a coordinated shift in signal responsiveness within the Nanog-low compartment, with cells becoming more responsive to FGF and Nodal signaling, as they lose Id1 expression. It is the increase in FGF responsiveness that best correlates with a higher probability of differentiating (model shown in Figure 3H).

### Id1 Is Responsible for Suppressing Differentiation within the Nanog-Low Compartment

We next asked how differentiation is suppressed within Id1-high cells. Eight genes are enriched in Id1-high cells (Figure S4), including Id1 itself. Id1 has previously been reported to block differentiation of naive and primed pluripotent cells (Aloia et al., 2015, Malaguti et al., 2013, Romero-Lanman et al., 2012, Ying et al., 2003, Zhang et al., 2010), but a role during the transition between these two states has not been explored.

**Figure 4:**
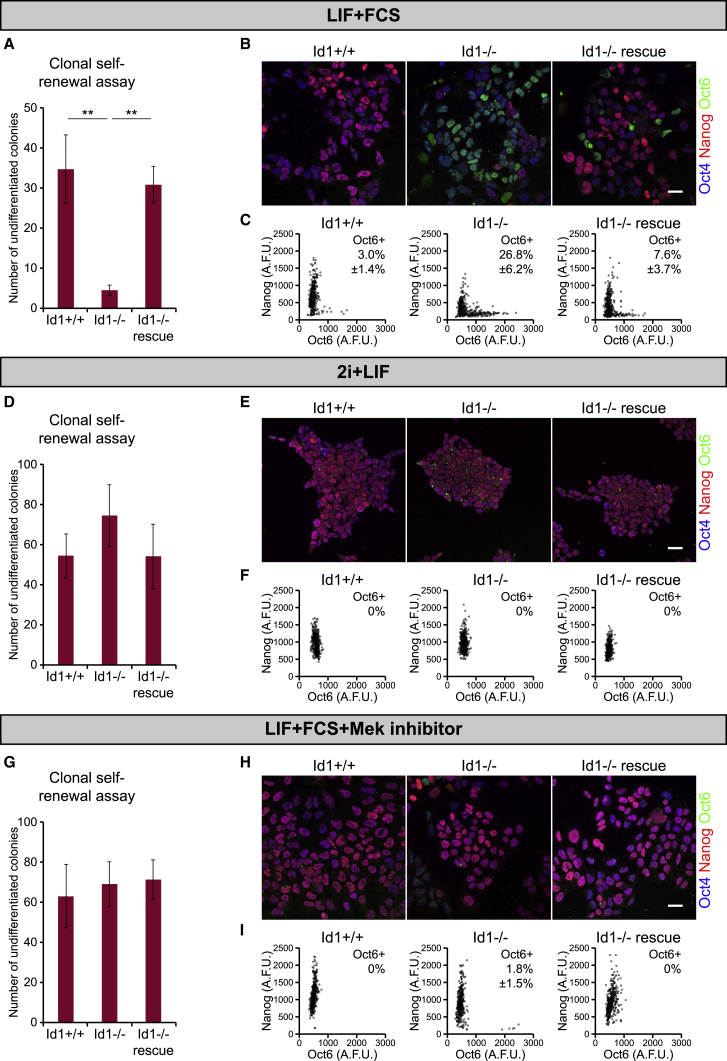
Id1 Is Responsible for Suppressing Differentiation within the Nanog-Low Compartment (A) Clonal self-renewal assays of wild-type, Id1-null, and Id1-rescue (Id1-null cells stably expressing an Id1 transgene) ESCs cultured in LIF + FCS. (B) Immunofluorescent staining of wild-type, Id1-null, and Id1-rescue ESCs cultured in LIF + FCS for Nanog, Oct4, and Oct6. (C) Quantification of the IF data in (B). (D) Clonal self-renewal assays of wild-type, Id1-null and Id1-rescue ESCs cultured in 2i + LIF. (E) Immunofluorescent staining of wild-type, Id1-null, and Id1-rescue ESCs cultured in 2i + LIF for Nanog, Oct4, and Oct6. (F) Quantification of the IF data in (E). (G) Clonal self-renewal assays of wild-type, Id1-null, and Id1-rescue ESCs cultured in LIF + FCS + 1 μM PD0325901 (a Mek inhibitor). (H) Immunofluorescent staining of wild-type, Id1-null, and Id1-rescue ESCs cultured in LIF + FCS + 1 μM PD0325901 for Nanog, Oct4, and Oct6. (I) Quantification of the IF data in (H). All data are represented as mean ± standard deviation of three independent experiments. Statistical analyses were performed using a one-way ANOVA followed by Tukey’s multiple comparison test. ^∗∗^p < 0.01. Plating density was 100 cells/well of a 6-well plate for clonal assays. Note that the height of the y axes differs between (A), (D), and (G). Immunofluorescence scale bars: 30 μm. See also Figure S4.

Id1-null ESCs have impaired clonogenic potential and display reduced levels of Nanog and increased levels of the primed pluripotency marker Oct6 (Figures 4A–4C). These phenotypes can be rescued by placing cells into 2i + LIF culture conditions in order to maintain uniform high levels of Nanog (Figures 4D–4F) or by addition of a Mek inhibitor (PD0325901) to LIF + FCS cultures in order to suppress FGF activity (Figures 4G–4I).

These data suggest that Id1 is dispensable within naive (Nanog-high) pluripotent cells but that it protects cells from differentiating after downregulation of Nanog.

### Id1 Dampens FGF Responsiveness

**Figure 5:**
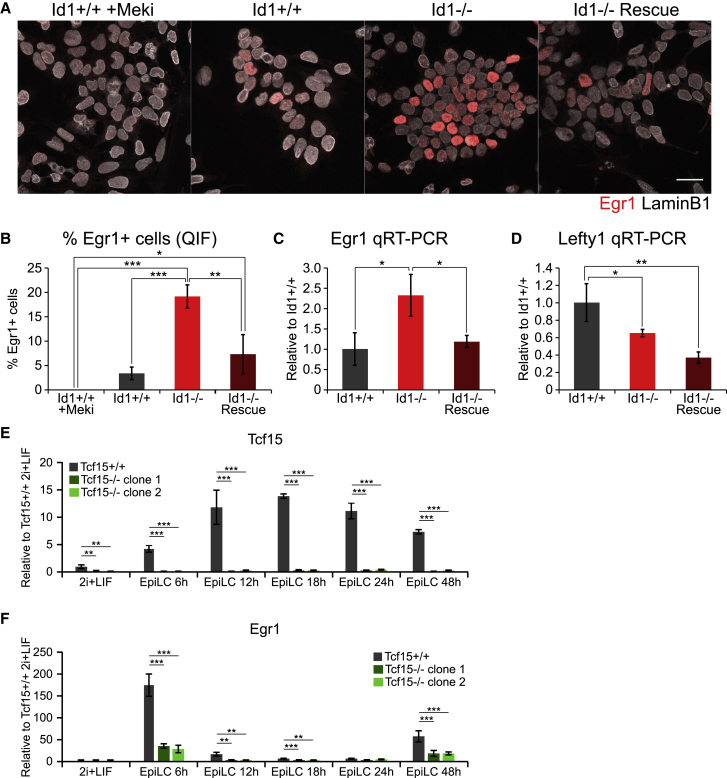
Id1 Dampens FGF Responsiveness by Modulating the Activity of Tcf15 (A) Immunofluorescent staining for Egr1 and LaminB1 in wild-type, Id1-null, Id1-rescue ESCs cultured in LIF + FCS. Wild-type cells cultured in LIF + FCS + 1 μM PD0325901 (+Meki) are included as a negative control. Scale bar, 30 μm. (B) Quantification of the IF data in (A). (C) qRT-PCR for the Fgf target *Egr1* in wild-type, Id1-null, and Id1-rescue ESCs cultured in LIF + FCS. (D) qRT-PCR for the Nodal target *Lefty1* in wild-type, Id1-null, and Id1-rescue ESCs cultured in LIF + FCS. (E) qRT-PCR for *Tcf15* in wild-type and two Tcf15-null clonal cell lines during 2i + LIF to EpiLC differentiation. (F) qRT-PCR for *Egr1* in the samples described in (E). Data are presented as mean ± standard deviation of three independent experiments. Statistical analyses were performed using a one-way ANOVA followed by Tukey’s multiple comparison test. ^∗^p < 0.05, ^∗∗^p < 0.01, ^∗∗∗^p < 0.001. See also Figure S5.

We next asked whether Id1 is responsible for suppressing FGF activity. Id1-null cells display increased expression of the FGF target gene Egr1, and this can be reversed by restoring Id1 expression (Figures 5A–5C). Nodal activity is also dampened in Id1-high cells (Figures 3B and 3C), but Id1-null cells do not have increased expression of the Nodal target gene *Lefty1* (Figure 5D), suggesting that Nodal signaling may regulate, rather than be regulated by, Id1 expression.

Egr1 is not only a passive readout of FGF activity; it also mediates the effects of FGF on pluripotent cells (Galonska et al., 2015). Egr1 is correlated with and controlled by Id1 in our experiments (Figures 3B–3D, 5A–5C, and S3B), so we asked how Id1 regulates Egr1.

E2A homodimers directly regulate Egr1 in pro-B cells (Lin et al., 2010), and E2A activity is repressed by Id1 (Massari and Murre, 2000). We therefore first considered E2A as a likely candidate for mediating the effects of Id1 on Egr1. However, this does not seem to be the case: *Egr1* does not respond to experimental activation of E2A homodimers in ESCs (Figures S5A and S5B).

We have previously identified Tcf15 as an Id-regulated pro-differentiation factor in ESCs (Davies et al., 2013). Transcriptome analysis of Tcf15-responsive genes indicates that Tcf15 upregulates *Egr1* (Davies et al., 2013). Using Tcf15-null cells, we find that Tcf15 is required for maximal *Egr1* expression after downregulation of Nanog (Figures 5E and 5F). These data are consistent with the idea that Id1 suppresses Egr1 expression through suppression of Tcf15 activity.

Taken together, our data suggest that Id1 orchestrates a coordinated shift in growth factor responsiveness and differentiation.

### Id1 Acts As a “Sensor” of Nodal Activity

We report above that there is a transient peak of Id1 protein expression during the transition from naive to primed epiblast states resulting from changes in “responsiveness” to BMP rather than changes in “exposure” to BMP (Figures 1C–1F, 1H, and 1I). We asked what is responsible for suppressing Id1 in naive cells.

**Figure 6:**
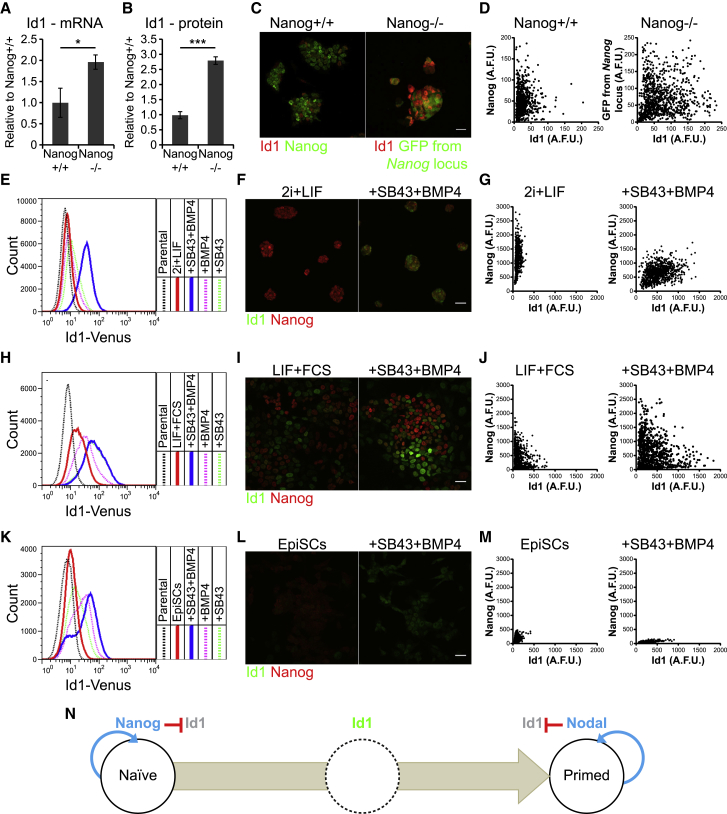
Dynamic Regulation of Id1 Expression during the Transition from Naive to Primed States (A) qRT-PCR for *Id1* in wild-type and Nanog-null ESCs cultured in LIF + FCS. Data are represented as mean ± standard deviation of three independent experiments. (B) Median Id1 protein expression following immunofluorescence quantification of Id1 staining in wild-type and Nanog-null ESCs cultured in LIF + FCS. Data are represented as mean ± standard deviation of three independent experiments. (C) Immunofluorescence for Id1 and Nanog or GFP in wild-type ESCs and in Nanog-null ESCs harboring a *GFP* transgene under the control of the *Nanog* promoter. (D) Quantification of Id1 and Nanog or GFP immunofluorescent signal in single wild-type or Nanog-null ESCs cultured in LIF + FCS. (E) Flow cytometry analysis of Id1-Venus ESCs cultured in 2i + LIF with or without stimulation with 10 ng/mL BMP4 and/or 10 μM of the Nodal inhibitor SB431542 for 48 h. (F) Immunofluorescence for Id1 and Nanog in wild-type ESCs cultured in 2i + LIF with or without 48 -h stimulation with 10 ng/mL BMP4 and 10 μM SB431542. (G) Quantification of immunofluorescence signal for the cells in (F). (H) Flow cytometry analysis of Id1-Venus ESCs cultured in LIF + FCS with or without 48-h stimulation with 10 ng/mL BMP4 and/or 10 μM SB431542. (I) Immunofluorescence for Id1 and Nanog in wild-type ESCs cultured in LIF + FCS with or without 48-h stimulation with 10 ng/mL BMP4 and 10 μM SB431542. (J) Quantification of immunofluorescence signal for the cells in (I). Id1 is enriched in Nanog-low cells. (K) Flow cytometry analysis of Id1-Venus EpiSCs with or without 48-h stimulation with 10 ng/mL BMP4 and/or 10 μM SB431542. (L) Immunofluorescence for Id1 and Nanog in wild-type EpiSCs with or without 48-h stimulation with 10 ng/mL BMP4 and 10 μM SB431542. (M) Quantification of immunofluorescence signal for the cells in (L). (N) Diagram illustrating the negative inputs of Nanog and Nodal on the expression of Id1. The results in Id1 being expressed only after Nanog is downregulated and before Nodal becomes active. Scale bars, 30 μm. Statistical analyses were performed using a two-tailed unpaired Student’s t test. ^∗^p < 0.05, ^∗∗^p < 0.01, ^∗∗∗^p < 0.001. See also Figure S6.

Nanog is able to repress Id1 expression (Figures S6A and S6B; Festuccia et al., 2012, Suzuki et al., 2006) and Id1 is derepressed in Nanog-null cells (Figures 6A and 6B). We examined Nanog-null ESCs in which the naive subpopulation can be identified via a fluorescent reporter targeted to the Nanog locus (Chambers et al., 2007). This confirmed that there is an overall increase in Id1 expression, although Id1 remains repressed in a subset of Nanog-null cells (Figures 6C and 6D). We conclude that Nanog contributes to, but is not solely responsible for, repression of Id1.

Nodal signaling is also able to repress Id1 expression (Galvin et al., 2010, Galvin-Burgess et al., 2013). The Nodal target gene *Lefty1* is enriched in Id1-low cells in LIF + FCS (Figures 3B and 3C) yet is not affected in Id1-null cells (Figure 5D), supporting the idea that Nodal signaling acts upstream rather than downstream of Id1.

In 2i + LIF, BMP4 is usually unable to upregulate Id1 (Figures 1C and 1D), but after addition of the Nodal inhibitor SB431542, almost all cells switch on Id1 in response to BMP4 (Figures 6E–6G). Similarly, in LIF + FCS cultures, SB431542 derepresses Id1, with the strongest increase observed within the Nanog-low subpopulation (Figures 6H–6J). SB431542 also permits BMP4-induced Id1 expression in EpiSCs (Figures 6K–6M). In keeping with these observations, treatment of 2i + LIF and LIF + FCS cultures with the Nodal agonist Activin A inhibits Id1 induction by BMP4 (Figures S6C and S6D). These data suggest that Nodal is the primary factor responsible for dampening Id1 expression in primed cells.

We conclude that Nanog and Nodal repress Id1 within naive cells and that Nodal also dampens Id1 expression within primed cells. This explains how Id1 can act as a “sensor” of Nodal activity after downregulation of Nanog (Figure 6N).

### Id1 Is Required for a Robust Transition from a Naive to a Primed Epiblast State *In Vivo*

Our findings suggest that Id1 protects epiblast cells from pro-differentiation cues from the time they lose Nanog expression through to the time that Nodal signaling begins to sustain them in a primed state.

**Figure 7:**
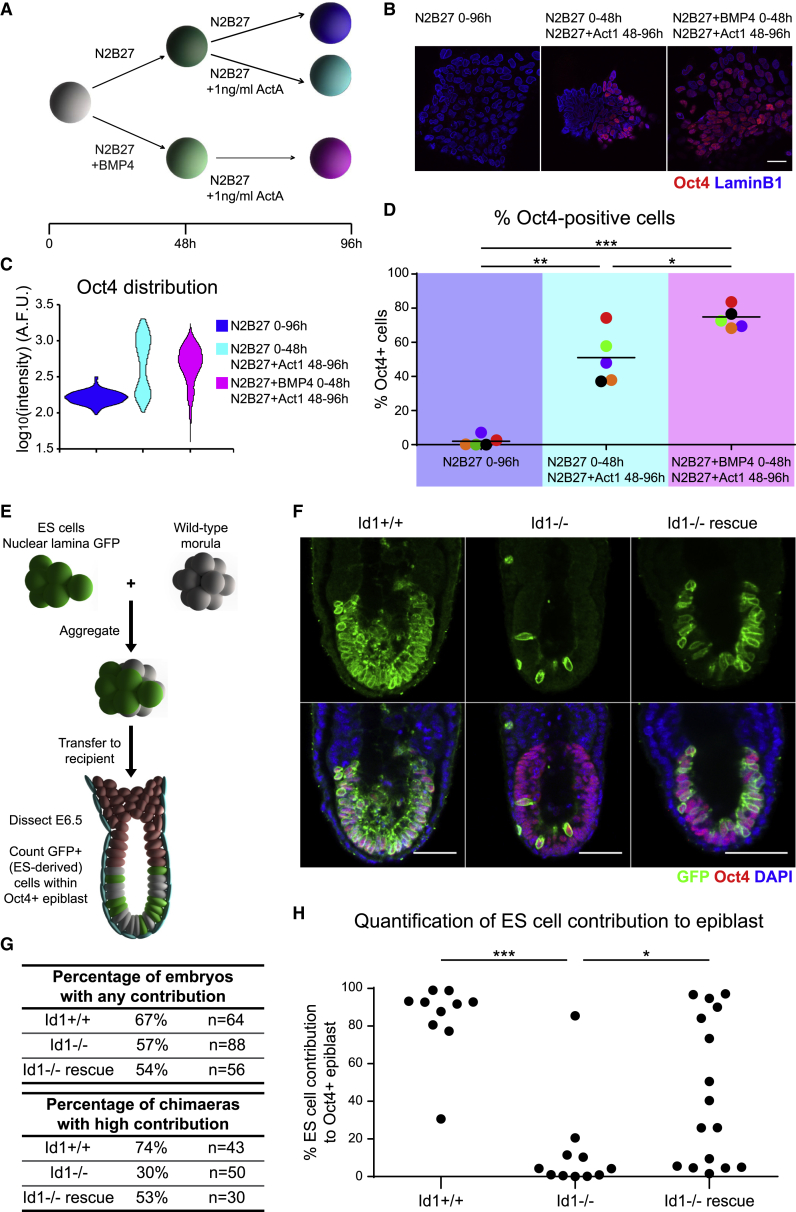
Id1 Enables a Robust Transition from a Naive to a Primed Pluripotent State (A) BMP4 enables a robust transition from naive to primed pluripotency *in vitro*. Experimental strategy: 2i + LIF cells were cultured in N2B27 in the presence or absence of 10 ng/mL BMP4 for 48 h. The cells were then exposed to low levels (1 ng/mL) of Activin A (ActA) for a further 48 h in the absence of BMP4 and assayed for their ability to retain Oct4 expression. Cells cultured in N2B27 throughout the experiment were used as a control for Oct4 downregulation. (B) Representative images of the samples described in (A). Act1: 1 ng/mL Activin A. Scale bar, 30 μm. (C) Distribution of Oct4 expression in the samples imaged in (B), calculated by immunofluorescence quantification. A.F.U.: Arbitrary fluorescence units. (D) Percentage of Oct4-positive cells observed in the samples imaged in (B), over 5 independent experiments (color-coded dots). Horizontal bars represent the mean of the 5 experiments. Statistical analysis was performed using a one-way ANOVA followed by Tukey’s multiple comparison test. ^∗^p < 0.05, ^∗∗^p < 0.01, ^∗∗∗^p < 0.001. (E) Id1 enables a robust transition from naive to primed pluripotency *in vivo*. Experimental strategy: labeled wild-type, Id1-null, and Id1-rescue ESCs were aggregated to wild-type morulae, then transferred to pseudopregnant females. The embryos were recovered at E6.5 and assessed for chimaerism. (F) Representative images of Id1^+/+^, Id1^−⁄−^, and Id1^−⁄−^ rescue chimeras, stained for GFP, Oct4, and DAPI. Scale bar, 30 μm. (G) Quantification of ESC contribution to recovered embryos and percentage of chimeras with high ESC contribution to the epiblast. Analysis performed by microscopy prior to fixation. (H) Quantification of ESC contribution to the Oct4-positive epiblast of recovered embryos. Analysis performed by nuclear segmentation and quantitative image analysis following immunofluorescence. Statistical analysis was performed using a Kruskal-Wallis test followed by Dunn’s multiple comparison test. ^∗^p < 0.05, ^∗∗∗^p < 0.001. See also Figure S7.

In keeping with this model, Id1 is dispensable under optimized differentiation conditions *in vitro*, where inappropriate pro-differentiation signals are eliminated (Figure S7A). We predict that Id1 should become important under sup-optimal signaling conditions such as those in the peri-implantation embryo where, for example, Nodal becomes activated in only a subset of Nanog-low cells (Granier et al., 2011). We devised an *in vitro* assay to mimic these conditions. We cultured pluripotent cells in basal media (N2B27) in order to allow cells to initiate exit from naive pluripotency in the absence of exogenous cues. After 48 h, we provided cells with low levels (1 ng/mL) of the Nodal agonist Activin A to approximate the incomplete activation of Nodal *in vivo* (Figure 7A). We used Oct4 (Pou5f1) to indicate the ability of these cells to retain an epiblast identity.

When this assay is carried out in the absence of BMP, only around half of cells retained Oct4 expression (Figures 7B–7D and S7B: note bimodal distribution of Oct4 in Figures 7C and S7B). Exposing cells to BMP in order to activate Id1 during the first 48 h increased the robustness with which cells progress through this transition, with the majority of cells maintaining Oct4 expression (Figures 7B–7D and S7B: note unimodal distribution of Oct4 in Figures 7C and S7B).

These results go some way toward supporting the hypothesis that BMP-Id1 helps to protect pluripotent cells from suboptimal signaling conditions. However, our *in vitro* assay falls far short of capturing the complexities of the dynamic signaling environment of the peri-implantation embryo. We therefore turned to an *in vivo* assay system.

We examined the efficiency with which Id1-null cells can persist throughout implantation and contribute to the post-implantation epiblast in aggregation chimeras (Figure 7E). Cells, which differentiate aberrantly or activate FGF prematurely during this process, are eliminated by cell competition (Clavería et al., 2013, Díaz-Díaz et al., 2017, Sancho et al., 2013).

Id1-null cells are able to contribute to the post-implantation epiblast: 57% of embryos contained at least some Id1-null ESCs, which is comparable to results from wild-type cells (67% of embryos) and Id1-rescue cells (54% of embryos) (Figures 7F and 7G). However, the degree of contribution was lower for Id1-null cells (30% high contribution) than wild-type (74% high contribution) or Id1-rescue cells (53% high contribution) (Figures 7F and 7G). Quantifying the number of ESCs that contribute to the post-implantation epiblast confirmed that Id1-null cells contribute to the epiblast less robustly than wild-type or Id1-rescue cells (Figures 7F–7H).

In contrast, Id1-null ESCs contribute efficiently to the pre-implantation pluripotent epiblast, displaying no sign of premature differentiation or cell death (Figures S7C–S7E). This confirms that Id1 is not required in naive pluripotent cells but becomes important during peri-implantation development.

We conclude that Id1 is required for a robust transition from the pre-implantation to the post-implantation epiblast *in vivo*.

## Discussion

### Coordinating Signaling with Differentiation during Transitions between Cell States

There has been much progress in understanding the signals and transcription factors that maintain naive and primed pluripotent cell states (Betschinger et al., 2013, Buecker et al., 2014, Galonska et al., 2015, Leeb et al., 2014, Yang et al., 2012). Much less is known about how pluripotency is protected during the transition between these states. We propose that cells regulate changes in signal responsiveness in order to protect pluripotency during this transition and that Id1 coordinates this process. In the absence of Id1, cells fail to transit robustly from pre-implantation to post-implantation stages of development.

Cells modulate signal responsiveness as they exit pluripotency (Kalkan et al., 2017, Zhou et al., 2013) and it has been proposed that prior to differentiation pluripotent cells enter a “transition state” or “formative” state in which they become more responsive to prevailing cues (Rué and Martinez Arias, 2015, Smith, 2017), an idea that is supported by our findings.

### How Are Cells Protected from Differentiation after the Collapse of the Naive GRN?

In culture, ESCs do not commit to a primed state immediately after downregulating Nanog, but rather can reassemble a naive GRN and revert to naive pluripotency (Chambers et al., 2007, MacArthur et al., 2012). A proportion of Nanog-low cells are nevertheless spontaneously lost to differentiation (Chambers et al., 2007). Several lines of evidence suggest that the decision of Nanog-low cells to regain Nanog or to differentiate is stochastic (Abranches et al., 2014, Kalmar et al., 2009, MacArthur et al., 2012). This might prompt the assumption that no particular factor is brought into play to determine the ability of cells to retain pluripotency and return to a Nanog-high state.

However, the following observations from peri-implantation embryos call this assumption into question: in peri-implantation embryos, in contrast to the situation in culture, cells that lose Nanog after E4.5 neither return to a Nanog-high naive state (Xenopoulos et al., 2015) nor differentiate into extraembryonic endoderm (Grabarek et al., 2012). Rather, they are efficiently captured into a post-implantation epiblast state that is dependent on Nodal (Camus et al., 2006, Mesnard et al., 2006). The existence of cells in the embryo that lack both Nanog and Nodal activity points to the existence of another factor that protects these cells from differentiation. We propose that this factor is Id1.

Recent findings indicate that extraembryonic endoderm potency is not irreversibly lost but rather remains latent in epiblast cells during implantation (Nowotschin et al., 2019). This implies the existence of mechanisms that protect the epiblast from differentiating into extraembryonic endoderm throughout the course of pregastrulation development. Nanog performs this role in the E3.5 embryo (Mitsui et al., 2003), and we now propose that Id1 takes over this role immediately after downregulation of Nanog. In support of this idea, we find that Id1 can protect Nanog-null cells from differentiating into primitive endoderm.

### BMP Maintains Epiblast Identity Specifically during the Transition between Naive and Primed States

*Id1* is a target of BMP signaling (Hollnagel et al., 1999) that contributes to maintenance of pluripotency in ESCs cultured in LIF + BMP4 or LIF + FCS (Ying et al., 2003, Ying and Smith, 2003). It has been proposed that Id1 maintains Nanog expression (Galvin-Burgess et al., 2013, Romero-Lanman et al., 2012), but this seems inconsistent with the observation that Id1 is not co-expressed with Nanog *in vitro* or *in vivo*. We reconcile our findings with these reports by proposing that Id1 does not directly maintain Nanog expression, but rather increases the probability that Nanog-low cells will return to a Nanog-high state.

Our findings also explain the previously puzzling observation that BMP is required for maintaining pluripotency in ESCs cultured as a mixture of naive and primed states in LIF + FCS (Malaguti et al., 2013, Ying et al., 2003, Zhang et al., 2010) yet is not required for maintaining pluripotency in homogenous populations of naive cells (Graham et al., 2014, Morikawa et al., 2016, Zhao, 2003) nor primed cells (Brons et al., 2007, Tesar et al., 2007). Our model is also consistent with the observation that BMP is not required for pre-implantation development (Graham et al., 2014, Zhao, 2003) but is required to maintain pluripotency subsequently (Di-Gregorio et al., 2007).

Much is known about the transcriptional regulators required to escape naive pluripotency and establish a primed state (Betschinger et al., 2013, Buecker et al., 2014, Galonska et al., 2015, Leeb et al., 2014, Yang et al., 2012). For instance, the FGF target gene *Egr1* drives reorganization of enhancer binding as cells proceed to a primed state (Galonska et al., 2015; Kumar and Ivanova, 2015). Our data place Id1 upstream of these factors, operating to suppress *Egr1* and thus help to transiently stabilize the naive state in the absence of Nanog. We cannot, however, exclude the possibility that factors other than Egr1 also act downstream of Id1.

### Id1 Confers Robustness to Early Development

Id1-null embryos progress through early development (Lyden et al., 1999), and Id1-null cells can differentiate *in vitro* (Romero-Lanman et al., 2012), so it is clear that Id1 is not absolutely required for early developmental transitions. Rather, we propose that Id1 makes early development more robust by shielding epiblast cells from pro-differentiation cues and ensuring that cells exit naive pluripotency only once signals to sustain the primed state are present. We confirm that Id1-null cells can proceed through early development in chimeric embryos but do so less robustly than their wild-type neighbors.

In summary, we propose that Id1 protects epiblast identity specifically during the transition from naive to primed states. As embryos progress through implantation, a build-up of Nodal simultaneously provides the environment that supports a primed epiblast state and suppresses expression of Id1 to permit the transition to this state.

Our findings support the idea that in order for changes in cell fate to occur at the correct time and place, mechanisms must exist to ensure that differentiation is coordinated with changes in responsiveness to extrinsic cues. Such mechanisms ensure canalization during early development (Waddington, 1959) and also help to explain why it is not straightforward to control differentiation of pluripotent cells *in vitro* simply by controlling exposure to extrinsic signals.

## STAR★Methods

### Key Resources Table

**Table:** 

REAGENT or RESOURCE	SOURCE	IDENTIFIER
**Antibodies**		

Rat Monoclonal Anti-Cdh1	Sigma-Aldrich	Cat#U3254; RRID: AB_477600
Rabbit Polyclonal Anti-Cleaved Caspase-3 (Asp175)	Cell Signaling Technology	Cat#9661; RRID: AB_2341188
Rabbit Monoclonal Anti-Egr1	Thermo Fisher	Cat#MA5-15009; RRID: AB_10982091
Goat Polyclonal Anti-Gata6	R&D	Cat#AF1700; RRID: AB_2108901
Chicken Polyclonal Anti-GFP	Abcam	Cat#ab3970; RRID: AB_300798
Goat Polyclonal Anti-Klf4	R&D	Cat#AF3158; RRID: AB_2130245
Rabbit Monoclonal Anti-Id1	Biocheck	Cat#BCH-1/37-2; RRID: AB_2713996
Rabbit Polyclonal Anti-LaminB1	Abcam	Cat#ab16048; RRID: AB_443298
Rat Monoclonal Anti-Nanog	Thermo Fisher	Cat#14-5761-80; RRID: AB_763613
Mouse Monoclonal Anti-Oct4	Santa Cruz	Cat#sc-5279; RRID: AB_628051
Goat Polyclonal Anti-Oct6	Santa Cruz	Cat#sc-11661; RRID: AB_2268536
Rabbit Monoclonal Anti-Phospho-p44/42 MAPK (Erk1/2) (Thr202/Tyr204)	Cell Signaling Technology	Cat#4370; RRID: AB_2315112
Rabbit Polyclonal Anti-tRFP	Evrogen	Cat#AB233; RRID: AB_2571743

**Chemicals, Peptides, and Recombinant Proteins**		

Benzyl alcohol	Alfa Aesar	Cat#100-51-6
Benzyl benzoate	Sigma	Cat#B9550
Tyrode’s solution, acidic	Sigma	Cat#1788
DAPI	Biotium	Cat#40043
Prolong Gold Antifade Mountant	Thermo Fisher	Cat#P36930
Doxycyline Hyclate	Sigma-Aldrich	Cat#D9891
Recombinant Human/Murine/Rat Activin A (E. coli derived)	Peprotech	Cat#120-14E
Recombinant Human BMP4	R&D	Cat#314-BP
Recombinant Human FGF basic	R&D	Cat#233-FB
CHIR 99021	Axon Medchem	Cat#1386
LDN 193189	Axon Medchem	Cat#1509
PD 0325901	Axon Medchem	Cat#1408
SB431542	Abcam	Cat#ab146590
B-27 Supplement	Gibco	Cat#17504044
N-2 Supplement	Gibco	Cat#17502048
KnockOut Serum Replacement (KSR)	Gibco	Cat#10828028
Fibronectin from bovine plasma solution	Sigma	Cat#F1141
Laminin	Sigma	Cat#L2020
Poly-L-ornithine solution 0.1%	Sigma	Cat#P4957
Lipofectamine 3000 Transfection Reagent	Life Technologies	Cat#L3000008
PerfectHyb Plus Hybridization Buffer	Sigma	Cat#H7033

**Critical Commercial Assays**		

Illumina TotalPrep RNA Amplification Kit	Ambion	Cat#AMIL1791

**Deposited Data**		

Raw and normalysed microarray data	This study	GEO: GSE108226

**Experimental Models: Cell Lines**		

*Mus musculus*: E14Ju09 mouse ESCs (129/Ola, male)	Hamilton and Brickman, 2014	N/A
*Mus musculus*: Id1V mouse ESCs (129/Ola, male)	Malaguti et al., 2013	N/A
*Mus musculus*: IVNR mouse ESCs (129/Ola, male)	This study	N/A
*Mus musculus*: 111B (*Id1+/+*) and 139 D (*Id1-/-*) mouse ESCs (129/Sv, sex unknown)	Romero-Lanman et al., 2012	N/A
*Mus musculus*: Id1-rescue mouse ESCs (129/Sv, sex unknown)	This study	N/A
*Mus musculus*: NLS-GFP-EmdTM labelled 111B, 139 D, Id1-rescue mouse ESCs (129/Sv, sex unknown)	This study	N/A
*Mus musculus*: TβC44cre6 (*Nanog-/-*) ESCs (129/Ola, male)	Chambers et al., 2007	N/A
*Mus musculus*: Nanog-rescue mouse ESCs (129/Ola, male)	This study	N/A
*Mus musculus*: *Tcf15-/-* ESCs (129/Ola, male)	This study	N/A
*Mus musculus*: 3xFlag-Id1 inducible mouse ESCs (129/Ola, male)	Malaguti et al. 2013	N/A
*Mus musculus*: A2lox.Cre ESCs (129/Ola, male)	Iacovino et al., 2011	N/A
*Mus musculus*: 3xFlag-E47-E47 inducible mouse ESCs (129/Ola, male)	This study	N/A

**Experimental Models: Organisms/Strains**		

*Mus musculus*: MF1: outbred	OLAC	N/A
*Mus musculus*: CD-1: outbred	Charles River	CD-1
*Mus musculus*: F1: B6CBAF1	Charles River	B6CBAF1

**Oligonucleotides**		

See Table S2 for list of primers used in this study	N/A	N/A

**Recombinant DNA**		

pPyCAG-NLS-GFP-EmdTM-Ires-Pac	This study	N/A
pPyCAG-tagRFP-IP backbone for subcloning of gene of interest	This study	N/A
p2loxCre	Iacovino et al., 2011	Addgene Plasmid #34635

**Software and Algorithms**		

R	R Core Team	http://www.R-project.org
Bioconductor	Gentleman et al., 2004	http://bioconductor.org
Beadarray R package	Dunning et al., 2007	http://bioconductor.org/packages/release/bioc/html/beadarray.html
Limma R package	Wettenhall and Smyth, 2004	https://bioconductor.org/packages/release/bioc/html/limma.html
NesSys	Blin et al., 2018	https://framagit.org/pickcellslab/nessys

### Lead Contact and Materials Availability

Further information and requests for resources and reagents should be directed to and will be fulfilled by the Lead Contact, Sally Lowell (sally.lowell@ed.ac.uk).

### Experimental Model and Subject Details

#### Animal Care and Use

Animal experiments were performed under the UK Home Office project license PEEC9E359, approved by the Animal Welfare and Ethical Review Panel of the University of Edinburgh and within the conditions of the Animals (Scientific Procedures) Act 1986.

#### Cell Lines

E14Ju09 ESCs are a male wild-type clonal cell line derived in-house from E14tg2a ESCs, with a 129/Ola genetic background (Hamilton and Brickman, 2014, Hooper et al., 1987). Id1V ESCs (male) were generated by targeting E14Ju09 ESCs with an Id1-Venus targeting construct (Malaguti et al., 2013, Nam and Benezra, 2009). IVNR ESCs (male) were generated by targeting Id1V ESCs with a Nanog-tagRFP targeting construct, which was obtained from Dr. Nicola Festuccia in Dr. Ian Chambers’ laboratory. Id1-null ESCs and control wild-type cells (129sv genetic background, sex unknown) were obtained from Dr. Robert Benezra (Romero-Lanman et al., 2012). Nuclear envelope GFP-labelled Id1-null and control wild-type clonal ESC lines were obtained by random integration of a *pPyCAG-NLS-GFP-EmdTM-IRES-Pac* construct. “Id1-rescue” clonal ESC lines were generated by random integration of a *pPyCAG-3xFlag-Id1-IRES-Pac* into unlabelled Id1-null ESCs, and of a *pPyCAG-3xFlag-Id1-IRES-HygroR* into labelled Id1-null ESCs. Nanog-null ESCs (TβC44cre6, male) were derived from E14tg2a ESCs and were obtained from Dr. Ian Chambers (Chambers et al., 2007). “Nanog-rescue” clonal ESC lines were generated by random integration of *pPyCAG-3xFlag-Nanog-IRES-Pac*, *pPyCAG-3xFlag-Id1-IRES-Pac* or *pPyCAG-3xFlag-GFP-IRES-Pac* constructs into Nanog-null ESCs. Tcf15-null ESCs (male) were derived from E14Ju09 ESCs by replacing *Tcf15* Exon 1 with a *Venus-polyA* transgene (CYL, SL in preparation). Inducible 3xFlag-Id1 ESCs (male) were generated by random integration of a *CAG-rtTA-IRES-Bls* construct and of a *tetO-3xFlag-Id1-Pgk-HygroR* construct into E14Ju09 ESCs (Malaguti et al., 2013). Inducible 3xFlag-E47-E47 ESCs (male) were generated making use of the A2lox inducible cassette exchange cell line (Iacovino et al., 2014).

#### Cell Culture

Mouse embryonic stem cells were routinely maintained on gelatinised culture vessels in Glasgow Minimum Essential Medium (GMEM, Sigma) supplemented with 10% foetal calf serum (FCS, APS), 100U/ml LIF (produced in-house), 100nM 2-mercaptoethanol (Gibco), 1X non-essential amino acids (Gibco), 2mM L-Glutamine (Gibco), 1mM Sodium Pyruvate (Gibco) (“LIF+FCS culture”). 2i+LIF culture was performed as previously described (Ying et al., 2008): cells were cultured in N2B27 medium supplemented with 1μM PD0325901 (Axon Medchem), 3μM CHIR99021 (Axon Medchem) and 100U/ml LIF (produced in-house) on culture vessels coated sequentially with poly-L-ornithine (Sigma) and 5μg/ml laminin (Sigma). N2B27 medium was prepared as previously described (Pollard et al., 2006). Its composition is a 1:1 mixture of DMEM/F12 (Gibco) and Neurobasal Medium (Gibco), supplemented with 0.5X N2 Supplement (Gibco), 0.5X B27 Supplement (Gibco), 2mM L-Glutamine (Gibco) and 100nM 2-mercaptoethanol (Gibco). Epiblast stem cells were derived from embryonic stem cells *in vitro* as previously described (Guo et al., 2009), by transferring ESCs to EpiSC culture medium on cell culture vessels coated with 7.5μg/ml fibronectin (Sigma), and passaging them every 1-2 days. EpiSCs were used for experimentation between passages 10 and 20. EpiSC culture medium composition is as previously described (Osorno et al., 2012): N2B27 medium supplemented with 10ng/ml Fgf2 (R&D) and 20ng/ml Activin A (R&D). EpiLC differentiation was performed as previously described (Hayashi et al., 2011). Briefly, 2i+LIF cells were plated on cell culture vessels coated with 7.5μg/ml fibronectin (Sigma) in EpiLC medium at a density of 2.5x10^4^ cells/cm^2^. EpiLC medium consists of N2B27 medium supplemented with 10ng/ml Fgf2 (R&D), 20ng/ml Activin A (R&D) and 1% KSR (Gibco). Medium was changed 24h after plating.Cells were cultured at 37°C in 5% CO_2_.

### Method Details

#### Plasmid Preparation

pPyCAG overexpression plasmids and p2lox cassette exchange plasmids were generated through conventional restriction enzyme-mediated ligation of DNA fragments flanked by convenient restriction sites. The DNA sequences of genes of interest were amplified from mouse ESC cDNA. The nuclear envelope GFP overexpression construct encodes a fusion protein comprising an N-terminal NLS, followed by GFP, and a C-terminal sequence consisting of the transmembrane domain of the inner nuclear membrane protein emerin (structure: *pPyCAG-NLS-GFP-EmdTM-IRES-Pac*).

#### Transfection

Overexpression plasmids were lipofected into cells using Lipofectamine 3000 reagent (Invitrogen), following the manufacturer’s instructions. p2lox cassette exchange were nucleofected into A2loxCre parental cells as previously described (Iacovino et al., 2014).

#### Embryo Collection

Pre- and peri-implantation embryos were obtained by flushing uteri with a large-bore blunted needle in M2 medium (Sigma). Post-implantation embryos were dissected at 5.5 and 6.5 d.p.c. in M2 medium. The sex of embryos used in this study was not determined.

#### Chimaera Generation

F1 female mice were superovulated (100 IU/ml PMSG, ProSpec, and 100 IU/ml HCG, Intervet, intraperitoneal injections 48h apart) and crossed with wild-type stud male mice. Pregnant mice were culled at 2.5 d.p.c. by cervical dislocation, ovaries with oviducts were dissected and collected in pre-warmed M2 medium. Oviducts were flushed using PBS and a 20-gauge needle attached to a 1ml syringe and filled with PB1. 2.5 d.p.c. embryos were collected and washed in PB1, the zona pellucida was removed using acidic Tyrode’s solution (Sigma), and transferred to a plate with incisions where one clump of 8-15 cells were added to each embryo. Embryos were then incubated at 37°C in 5% CO_2_ for 24h prior to transfer to pseudopregnant recipients, or for up to 72h for assessment of pre-implantation chimaerism. Blastocysts were selected and collected to be transferred into the uterus of a pseudopregnant CD-1 female. Embryos were dissected at 6.5 d.p.c. in M2 medium and observed for chimeric ESC contribution under an Olympus IX51 microscope, prior to fixation and immunostaining.

#### Embryo Immunofluorescence and Confocal Microscopy

Embryos were fixed with 4% formaldehyde/PBS/0.1% Triton X-100 (Sigma) for 10 (pre-implantation), 20 (peri-implantation) or 30 (post-implantation) minutes and quenched with 50mM ammonium chloride. Cellular permeabilization was carried out for 10 min in PBS/0.1% Triton X-100. The embryos were incubated in primary antibody in 3% donkey serum/PBS/0.1% Triton X-100 overnight, and subjected to 3 washes in PBS/0.1% Triton X-100. Secondary antibodies were applied subsequently for 2h to overnight, followed by 3 washes in PBS/0.1% Triton X-100. Embryos were then stained with DAPI (Biotium), mounted in PBS droplets covered with mineral oil in “microscope rings”, and imaged on a Leica SP8 confocal microscope. Alternatively, following staining, chimaeric embryos requiring immunostaining quantification were dehydrated in methanol series in PBS/0.1% Triton X-100, clarified in 50% methanol/50% BABB (benzyl alcohol:benzyl benzoate 1:2 ratio, Alfa Aesar and Sigma), transferred into 100% BABB in glass capillaries and imaged on a Leica SP8 confocal microscope.

#### Cell Immunofluorescence and Confocal Microscopy

Cells for immunofluorescence were cultured on flamed glass coverslips coated with 7.5μg/ml fibronectin (for adherent culture), or cytospun onto polysine adhesion slides (Thermo Fisher) using a Shandon Cytospin 3 centrifuge (for sorted samples in suspension). They were fixed in 4% formaldehyde/PBS, quenched with 50mM ammonium chloride, blocked in 3% donkey serum and 0.1% Triton X-100. Cells were then incubated with primary antibody for 3 h at room temperature, washed 3 times with PBS, incubated with secondary antibody and/or 100ng/ml DAPI for 1h at room temperature, washed 3 times with PBS, mounted in ProLong Gold Antifade Mountant (Molecular Probes), and imaged on a Leica SP8 confocal microscope. Where recommended by antibody manufacturers, a methanol permeabilisation step was included prior to blocking.

#### Immunofluorescence Quantification

Nuclear immunofluorescence signal was quantified using nuclear segmentation based on nuclear envelope staining or DAPI, as well as manual editing of segmentation results, making use of the NesSys software described in (Blin et al., 2018).

#### Gene Expression Analysis

Total RNA was extracted from cells making use of the Absolutely RNA Miniprep Kit (Stratagene). 300ng total RNA were reverse transcribed into cDNA making use of M-MLV Reverse Transcriptase (Invitrogen). qRT-PCR was performed using the Universal ProbeLibrary system (Roche) with a Lightcycler 480 II instrument. Expression data are presented relative to the geometric mean of the housekeeping genes *Sdha*, *Tbp* and *Ywhaz*. The sequences of the primers used in this study are listed in Table S2.

#### Flow Cytometry

Cells were dissociated into single cell suspensions in ice-cold PBS+10% FCS, in the presence of either 100ng/ml DAPI or 1μg/ml propidium iodide to stain dead cells. Analysis of fluorescence was performed on a BD FACSCalibur. Cell sorting was performed on a BD FACSAria.

#### Clonal Self-renewal Assays and Alkaline Phosphatase Staining

Cells were plated 10-30 cells/cm^2^ in media as indicated in figure legends, and media were changed every other day. After 7 days, alkaline phosphatase staining was performed using the Leukocyte Alkaline Phosphatase Kit (Sigma).

#### Transcriptome (Microarray) Analyses

Sample preparation for microarrays was performed as previously described (Davies et al., 2013). 100 ng of total RNA were reverse transcribed into double-stranded cDNA and transcribed/amplified into biotin labelled cRNA using an Illumina TotalPrep RNA Amplification Kit (Ambion). Labelled RNA was submitted to the WTCRF MRC Human Genetics Unit (University of Edinburgh) for further processing. cRNA quality was checked using an Agilent 2100 Bioanalyser and hybridisation performed on an MouseWG-6 v2 BeadChip (Illumina). Raw data were processed in R using the beadarray (Dunning et al., 2007) and limma (Wettenhall and Smyth, 2004) packages from the Bioconductor suite (Gentleman et al., 2004). Briefly, expression data were quantile-normalised and log_2_-transformed before assessing differential expression with the limma algorithms. Principal component analysis was performed using the prcomp() function in the core R stats package. Quantile-normalised microarray data are available in Table S1.

#### Western Blotting

Cells were lysed in RIPA buffer + 1X PMSF (Alpha Diagnostics). 20μg protein lysates were run on 4%-12% NuPage Bis-Tris Gel (Novex) and transferred onto Amersham Hybond ECL Nitrocellulose Membrane (GE Healthcare). Membranes were blocked in 5% Amersham ECL Prime Blocking Agent (GE Healthcare) + 0.1% Tween 20 (Sigma) in PBS. Membranes were incubated in primary antibody overnight at 4°C, washed 3 times in PBS + 0.1% Tween 20, incubated in HRP-conjugated secondary antibody for 1 h at room temperature and washed 3 times in PBS + 0.1% Tween 20. The membrane was incubated in Amersham ECL Western Blotting Detection Reagent (GE Healthcare) or Amersham ECL Prime Western Blotting Detection Reagent (GE Healthcare), depending on the expected strength of signal. The membranes were used to expose Amersham Hyperfilm ECL (GE Healthcare), and films were developed using a Konica SRX-101A Medical Film Processor.

#### Southern Blotting

Genomic DNA was extracted from mouse ESCs using the DNeasy Blood and Tissue kit (Qiagen). Southern blotting was performed as previously described (Southern, 1975). Briefly, 5μg genomic DNA was digested with 100U BamHI-HF (NEB) overnight at 37°C, in the presence of 2.5mM spermidine (Sigma). The DNA was ethanol-precipitated, resuspended in 20μl dH_2_O, and run on a 0.8% w/v agarose/TAE gel. λ DNA-HindIII digest was loaded as a size marker. The gel was placed in denaturing solution (aqueous solution of 86.77g/l sodium chloride + 20g/l sodium hydroxide) for 40 minutes at room temperature, and neutralising solution (aqueous solution of 116.8g/l sodium chloride, 121.1g/l Tris base, pH8.0) for 40 minutes at room temperature. DNA was transferred onto a positively charged nylon membrane (Roche) by capillary transfer of 20X SSC buffer (aqueous solution of 175.2g/l sodium chloride + 88.2g/l Tris base, pH7.4) for 48 h at room temperature. The membrane was then baked for 1 h at 120°C, rinsed in 2X SSC, and placed in a glass hybridisation bottle with PerfectHyb Plus (Sigma) hybridisation buffer at 65°C for 1 h. Probes were generated by PCR amplification of sequences of interest (Table S2), and labelled with [α-^32^P]dCTP using the Amersham Rediprime II DNA Labelling System (GE Healthcare), alongside the control λ HindIII DNA to detect the size marker. 500μl sonicated herring sperm DNA (Sigma) and the probes were added to the hybridisation bottle overnight at 65°C. The hybridisation solution was removed, and the membrane was washed twice for 15 minutes in 2X SSC+0.1% SDS, and once for 30 minutes in 0.5X SSC+0.1% SDS. The membrane was used to expose Amersham Hyperfilm ECL (GE Healthcare), and films were developed using a Konica SRX-101A Medical Film Processor.

### Quantification and Statistical Analysis

Definition of statistical significance and size of n is indicated in Figure Legends. Statistical analysis methods include two-tailed Student’s t-test for comparison of two samples, one-way ANOVA followed by Tukey’s multiple comparison test for comparison of more than two samples with normal distributions, Kruskal-Wallis test followed by Dunn’s multiple comparison test for comparison of more than two samples with non-normal distributions, and empirical Bayes moderated t-statistics for linear model fit contrasts for microarray data, with p-values adjusted for multiple testing correction using the Benjamini & Hochberg method.

### Data and Code Availability

The accession number for the raw and normalized microarray data reported in this paper is GEO: GSE108226.

## Supplemental Information

Document S1. Figures S1–S7 and Table S2

Table S1. Quantile-Normalized Microarray Data, Related to Figures 2 and 3

Document S2. Article plus Supplemental Information
