# Supplementary material for: Id1 Stabilizes Epiblast Identity by Sensing Delays in Nodal Activation and Adjusting the Timing of Differentiation
Source: Dev Cell. 2019 Aug 19;50(4):462–477.e5. doi: 10.1016/j.devcel.2019.05.032 (PMC6706657; doi:10.1016/j.devcel.2019.05.032)
Supplement: Document S1. Figures S1–S7 and Table S2 [file mmc1.pdf]

Developmental Cell, Volume *50*

Supplemental Information

Id1 Stabilizes Epiblast Identity by Sensing Delays in Nodal Activation and Adjusting the Timing of Differentiation

Mattias Malaguti, Rosa Portero Migueles, Guillaume Blin, Chia-Yi Lin, and Sally Lowell

FIGURE S1

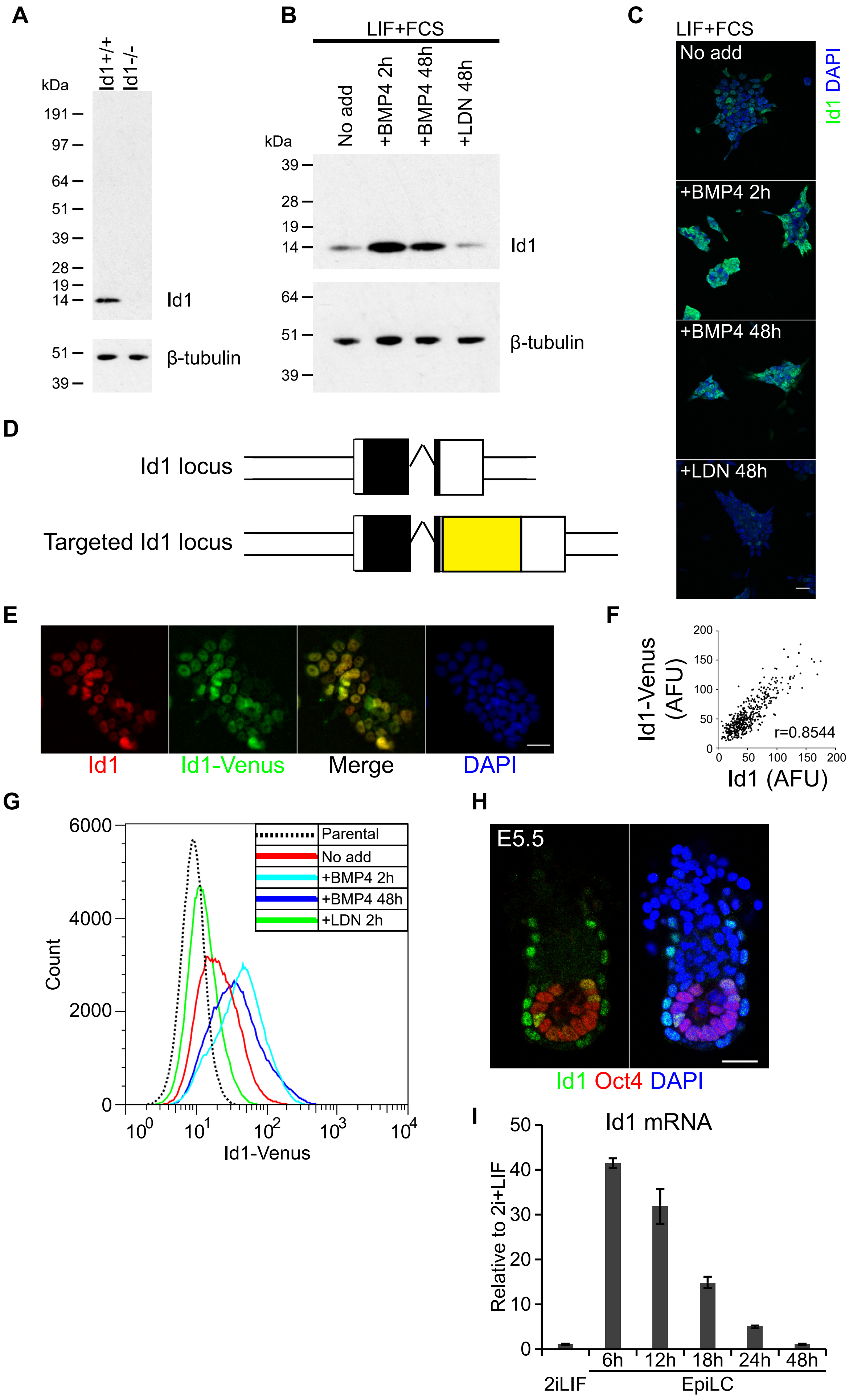
A
Id1+/+
Id1-/-
kDa
191
97
64
51
39
28
19
14
Id1
51
β-tubulin
39
B
LIF+FCS
No add
+BMP4 2h
+BMP4 48h
+LDN 48h
kDa
39
28
19
14
Id1
64
51
β-tubulin
39
C
LIF+FCS
No add
Id1 DAPI
+BMP4 2h
+BMP4 48h
+LDN 48h
D
Id1 locus
Targeted Id1 locus
E
Id1
Id1-Venus
Merge
DAPI
F
Id1-Venus (AFU)
200
150
100
50
0
r=0.8544
0 50 100 150 200
Id1 (AFU)
G
6000
4000
2000
0
Count
Parental
No add
+BMP4 2h
+BMP4 48h
+LDN 2h
10^0 10^1 10^2 10^3 10^4
Id1-Venus
H
E5.5
Id1 Oct4 DAPI
I
Id1 mRNA
50
40
30
20
10
0
Relative to 2i+LIF
6h
12h
18h
24h
48h
2iLIF
EpiLC

Figure S1. Id1 immunodetection and the Id1-Venus reporter both provide faithful readouts of BMP responsiveness, Related to Figure 1.

(A) Western blot for Id1 in wild-type and Id1-null ES cells illustrates the high specificity of the Id1 antibody. B-tubulin is included as a loading control.

(B) Western blot for Id1 in wild-type ES cells cultured in LIF+FCS +/- 10ng/ml BMP4 for 2h or 48h, or with the addition of 100nM of the BMP inhibitor LDN193189 (LDN) for 48h. As expected, Id1 is upregulated upon BMP4 stimulation and downregulated upon BMP inhibition with LDN. B-tubulin is included as a loading control.

(C) Immunofluorescence staining of wild-type ES cells cultured in LIF+FCS with no addition, with the addition of 10ng/ml BMP4 for 2h or 48h, or with the addition of 100nM of the BMP inhibitor LDN193189 (LDN) for 48h. As expected, Id1 is upregulated upon BMP4 stimulation and downregulated upon BMP inhibition with LDN. Scale bar: 30μm.

(D) Diagrammatic structure of the Id1 loci in Id1-Venus reporter ES cells. Venus is fused to the C-terminus of Id1 via a flexible linker.

(E) Co-staining for Id1 and Venus in Id1-Venus cells cultured in LIF+FCS. Scale bar: 30μm.

(F) Immunofluorescence quantification of the cells in (E) show high correlation between Id1 and Venus expression (Pearson's r=0.8544). A.F.U.: arbitrary fluorescence units.

(G) Flow cytometry analysis of Id1-Venus cells cultured in LIF+FCS with no addition, with the addition of 10ng/ml BMP4 for 2h or 48h, or with the addition of 100nM of the BMP inhibitor LDN193189 (LDN). Parental non-fluorescent cells are included as a control. As expected, Id1-Venus is upregulated upon BMP4 stimulation and downregulated upon BMP inhibition with LDN.

(H) Co-staining for Id1 and Oct4 in E5.5 mouse embryo. Id1 is co-expressed with Oct4 in a subset of cells in the proximal region of the epiblast, and is expressed in the visceral endoderm.

(I) qRT-PCR for *Id1* in 2i+LIF to EpiLC differentiation of E14Ju09 ES cells. Error bars: standard deviation of 3 biological replicates.

FIGURE S2 - Malaguti et al

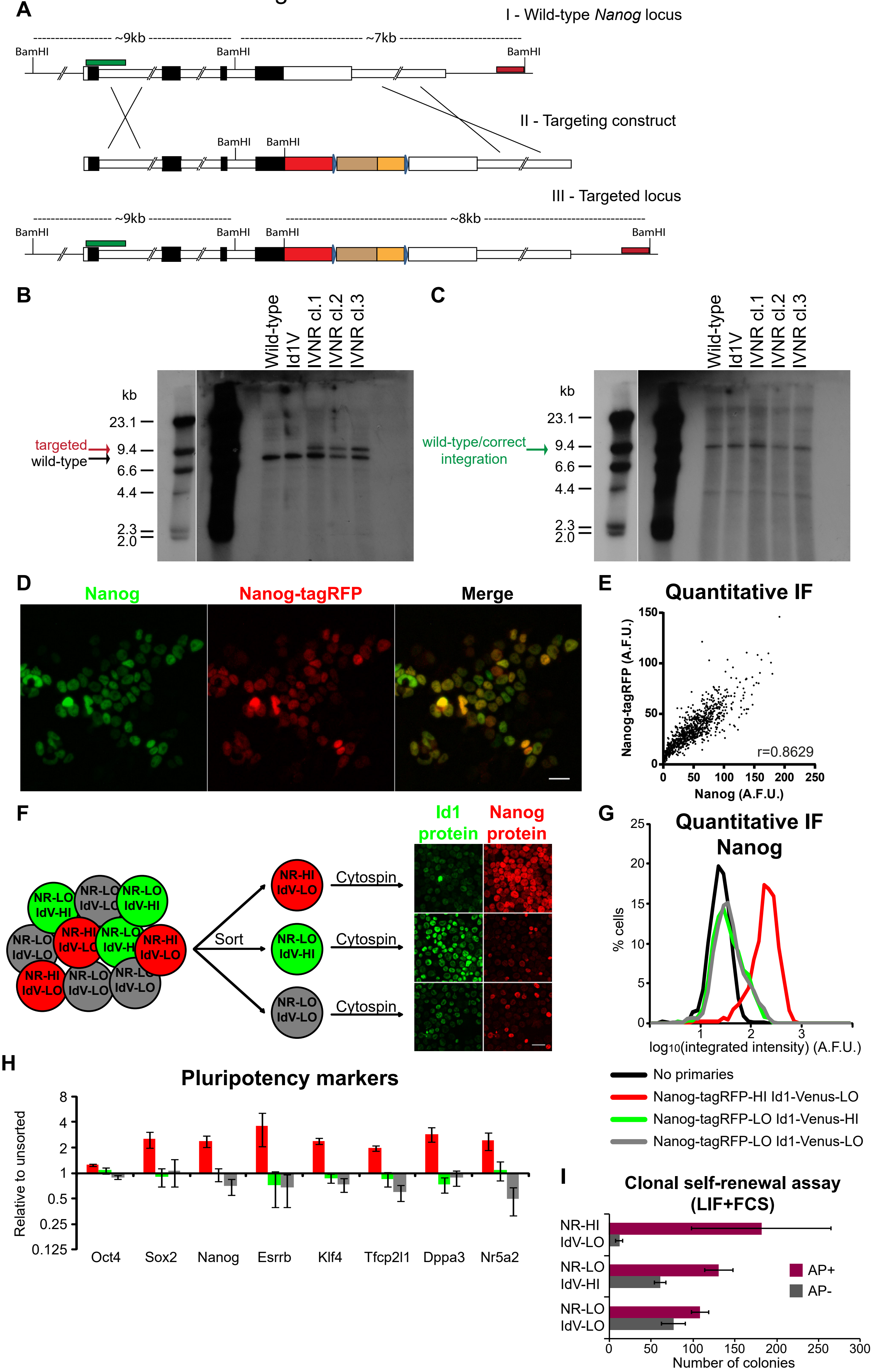

Figure S2. Characterisation of Id1-Venus Nanog-tagRFP reporter ES cells and their sorted subpopulations, Related to Figure 2.

(A) Strategy to target the Nanog locus with a *Nanog-tagRFP* fusion construct. Black boxes: exonic DNA, large white boxes: UTRs, medium white boxes: intronic DNA, single black line: intergenic DNA, large red box: *tagRFP*, brown box: *IRES*, yellow box: *Bls* resistance gene, blue triangles: *loxP* sites, medium red box: Southern blot targeting probe, medium green box: Southern blot multiple integration probe. Sizes of expected bands following BamHI restriction digest are indicated.

(B) Southern blot with targeting probe on BamHI-digested genomic DNA extracted from wild-type ES cells, Id1-Venus parental cells (Id1V) and three different Id1-Venus Nanog-tagRFP (IVNR) clones. All three clones present the expected banding pattern for correctly targeted clones. A lower exposure of the λ DNA-HindIII digest ladder is included to the left of the blot.

(C) Southern blot with multiple integration probe on BamHI-digested genomic DNA extracted from wild-type ES cells, Id1-Venus parental cells (Id1V) and three different Id1-Venus Nanog-tagRFP (IVNR) clones. All three clones present the expected banding pattern for correctly targeted clones. A lower exposure of the λ DNA-HindIII digest ladder is included to the left of the blot.

(D) Co-staining for Nanog and tagRFP in IVNR cells cultured in LIF+FCS. Scale bar: 30μm.

(E) Immunofluorescence quantification of the cells in (D) show high correlation between Nanog and tagRFP expression (Pearson’s r=0.8629). A.F.U.: arbitrary fluorescence units.

(F) Sorting of IVNR ES cells cultured in LIF+FCS into 3 subpopulations based on Id1-Venus and Nanog-tagRFP expression: Nanog-tagRFP-high Id1-Venus-low (NR-HI IdV-LO), Nanog-tagRFP-low Id1-Venus-high (NR-LO IdV-HI) and Nanog-tagRFP-low Id1-Venus-low (NR-LO IdV-LO). Cytospin of the three subpopulations was performed immediately after sorting, and cells were stained for Id1 and Nanog expression. Scale bar: 30μm.

(G) Immunostaining quantification of Nanog expression in the three sorted subpopulations in (F) reveals the presence of similar low numbers of Nanog-medium cells in the two NR-LO subpopulations.

(H) qRT-PCR analysis of the sorted samples in (F) reveal similar levels of pluripotency marker expression between the two NR-LO subpopulations, with the exception of *Nr5a2*, which is higher in NR-LO IdV-HI than NR-LO IdV-LO ES cells. Data are represented as mean ± SEM of seven independent experiments.

(I) Clonal self-renewal assays for cells sorted in (F) reveal no significant differences in alkaline phosphatase-positive colony-forming ability between NR-LO IdV-HI and NR-LO IdV-LO cells. 300 cells were plated on $10cm^2$ for each sample. Data are represented as mean ± SEM of three independent experiments.

FIGURE S3

A - LIF+FCS - No significant enrichment in NR-LO IdV-LO vs NR-LO IdV-HI

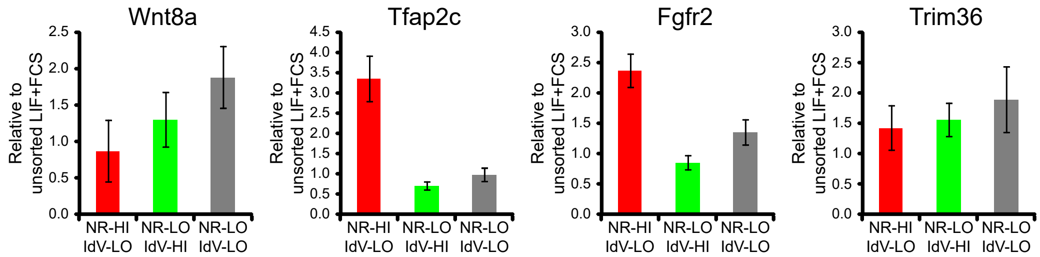

B - LIF+FCS Nanog-/-

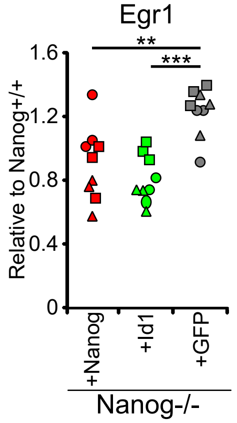

C - 6h N2B27 - Naïve pluripotency

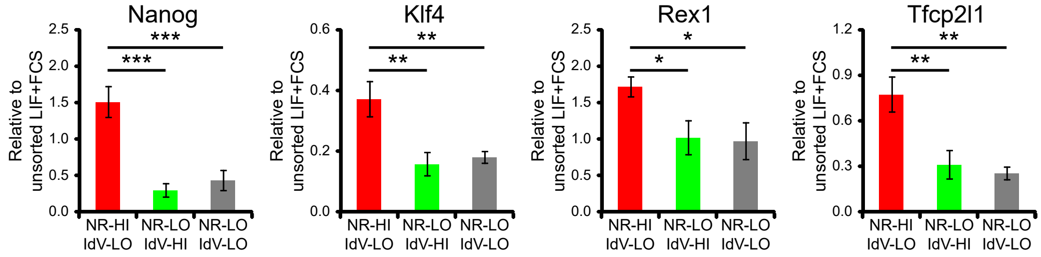

D - 6h N2B27 - General and primed pluripotency

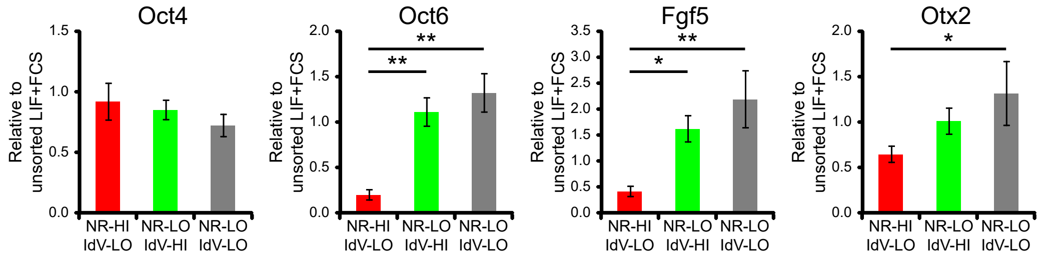

Figure S3. Comparison of Nanog-low Id1-low and Nanog-low Id1-high cells, Related to Figure 3.

(A) qRT-PCR experiments showing lack of statistically significant (one-way ANOVA followed by unpaired t-test, $p<0.05$) differential expression between NR-LO IdV-LO and NR-LO IdV-HI subpopulations for transcripts in Figure 3B. Data are presented as mean ± SEM of seven independent experiments. Validation of transcripts that showed statistically significant expression (*Egr1*, *Lefty1*) is shown in Figure 3C.

(B) qRT-PCR analysis for *Egr1* of Nanog-rescue cells cultured in LIF+FCS. Each shape represents a different clonal line.

(C) qRT-PCR analysis of naïve pluripotency marker expression in sorted subpopulations after the 6h N2B27 differentiation challenge.

(D) qRT-PCR analysis of general and primed pluripotency marker expression in sorted subpopulations after the 6h N2B27 differentiation challenge.

Data in (C) and (D) are represented as mean ± SEM of three independent experiments. Statistical analyses (unless specified): one-way ANOVA followed by Tukey's multiple comparison test. *$p<0.05$, **$p<0.01$, ***$p<0.001$.

FIGURE S4

A - Transcriptome data

Transcipts enriched in NR-LO IdV-HI vs NR-LO IdV-LO

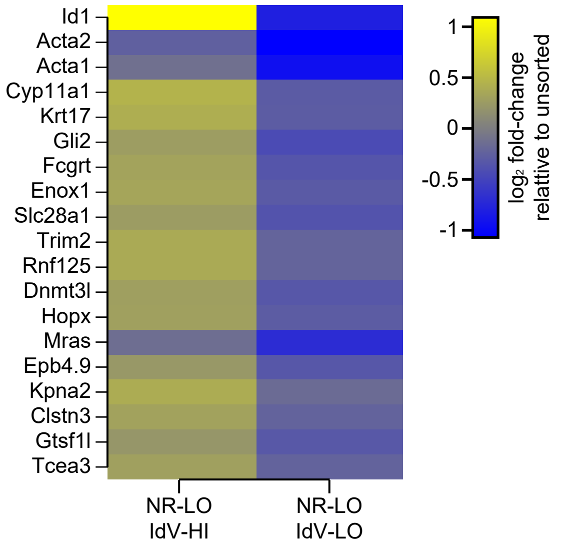

B - qRT-PCR - Significant enrichment in NR-LO IdV-HI vs NR-LO IdV-LO LIF+FCS

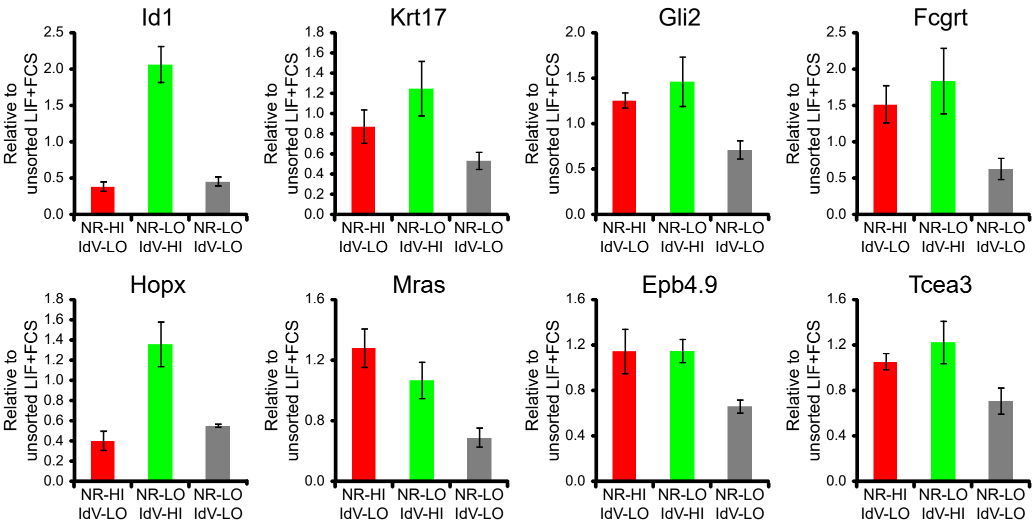

C - qRT-PCR - No significant enrichment in NR-LO IdV-HI vs NR-LO IdV-LO LIF+FCS

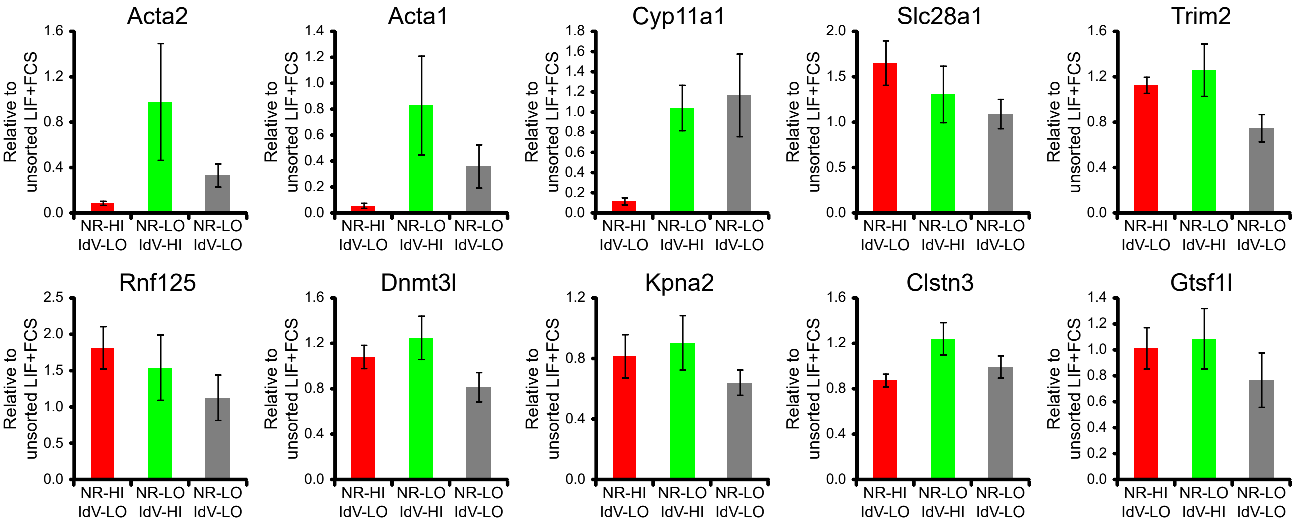

Figure S4. Transcripts enriched in Nanog-low Id1-high vs Nanog-low Id1-low cells, Related to Figure 4.

(A) Heatmap of transcripts significantly enriched in NR-LO IdV-HI relative to NR-LO IdV-LO subpopulations. Significance was defined as $\log_2$(fold-change)>0.5, p-value adjusted for multiple testing correction <0.5. Methods for statistical analysis of transcriptomic data are described in the STAR Methods section.

(B) qRT-PCR experiments showing statistically significant (ANOVA followed by unpaired t-test, $p<0.05$) differential expression between NR-LO IdV-HI and NR-LO IdV-LO subpopulations for transcripts in (A).

(C) qRT-PCR experiments showing lack of statistically significant (ANOVA followed by unpaired t-test, $p<0.05$) differential expression between NR-LO IdV-HI and NR-LO IdV-LO subpopulations for transcripts in (A).

qRT-PCR data are represented as mean ± SEM of seven independent experiments.

FIGURE S5

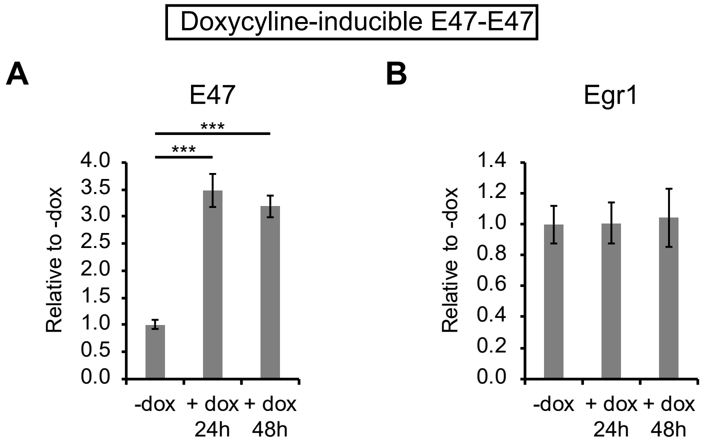

Figure S5. E47 homodimers do not induce Egr1 expression in ES cells, Related to Figure 5.

Mouse ES cells harbouring a doxycycline-inducible 3xFlag-E47-E47 transgene were cultured in LIF+FCS for 48h in the absence ("-dox") or the presence ("+dox") of 1µg/ml doxycycline (dox) for 24h or 48h. qRT-PCRs were performed to assess the expression of (A) *E47* and (B) *Egr1*. Data are presented as mean ± SEM of six independent experiments.

FIGURE S6

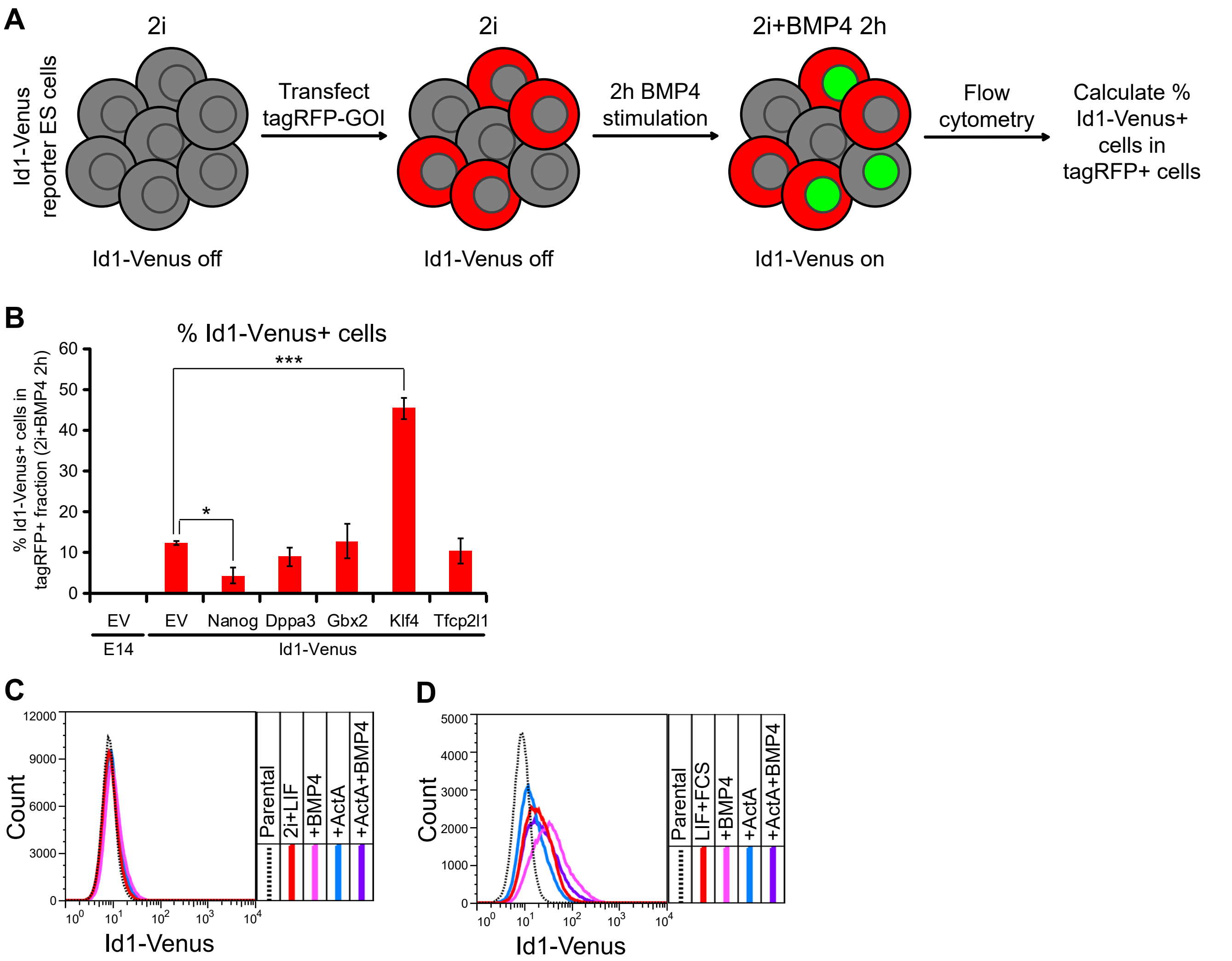

Figure S6. Nanog and Activin A repress Id1-Venus expression, Related to Figure 6.

(A) Experimental strategy. Id1-Venus reporter ES cells were cultured in 2i, a condition in which Id1-Venus is not expressed. Overexpression plasmids encoding tagRFP fused to genes of interest were lipofected into the cultures. After 48h, the cells were treated with 10ng/ml BMP4 for 2h to enable Id1-Venus expression (unlike 2i+LIF, Id1 is expressed in response to BMP4 in 2i), then subject to flow cytometry analysis. The percentage of cells upregulating Id1-Venus within the tagRFP-positive compartment was calculated.

(B) Quantification of the experiment described in (A). Five pluripotency transcription factors were tested, and of these only Nanog was able to repress Id1-Venus expression. EV: tagRFP-empty vector.

Data are presented as mean ± SEM of three independent experiments. Statistical analyses were performed using a one-way ANOVA, followed by Dunnett's multiple comparison test of all genes of interest with the empty vector control. $^{*}p<0.05$, $^{***}p<0.001$.

(C) Flow cytometry analysis of Id1-Venus ES cells cultured in 2i+LIF with or without 48h stimulation with 10ng/ml BMP4 and/or 20ng/ml Activin A.

(D) Flow cytometry analysis of Id1-Venus ES cells cultured in LIF+FCS with or without 48h stimulation with 10ng/ml BMP4 and/or 20ng/ml Activin A.

FIGURE S7

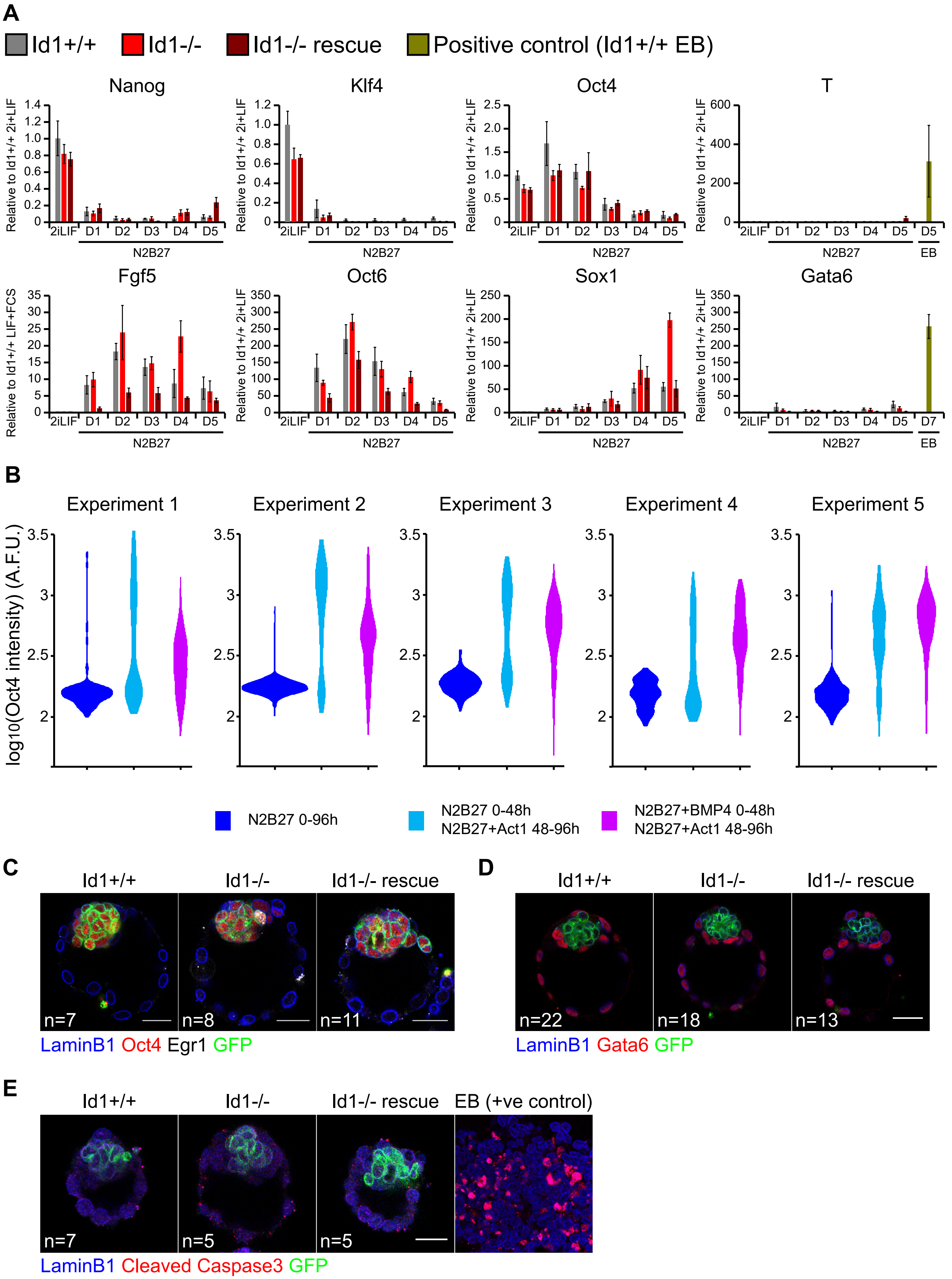

A
Id1+/+
Id1-/-
Id1-/- rescue
Positive control (Id1+/+ EB)
Nanog
Klf4
Oct4
T
Relative to Id1+/+ 2i+LIF
2iLIF
D1
D2
D3
D4
D5
N2B27
EB
Fgf5
Oct6
Sox1
Gata6
Relative to Id1+/+ LIF+FCS
D7
B
Experiment 1
Experiment 2
Experiment 3
Experiment 4
Experiment 5
log10(Oct4 intensity) (A.F.U.)
N2B27 0-96h
N2B27 0-48h
N2B27+Act1 48-96h
N2B27+BMP4 0-48h
N2B27+Act1 48-96h
C
Id1+/+
Id1-/-
Id1-/- rescue
n=7
n=8
n=11
LaminB1 Oct4 Egr1 GFP
D
n=22
n=18
n=13
LaminB1 Gata6 GFP
E
EB (+ve control)
n=7
n=5
n=5
LaminB1 Cleaved Caspase3 GFP

Fig S7. Id1 enables a robust transition from naïve to primed pluripotency in the face of conflicting signals, Related to Figure 7.

(A) qRT-PCR of wild-type, Id1-null and Id1-rescue ES cells undergoing 2i+LIF --> N2B27 differentiation. All cell lines are able to exit pluripotency, with Id1-null cells displaying a pro-neural phenotype. Positive control samples from wild-type embryoid bodies (EB) are included for *T* and *Gata6*. Data are presented as mean ± standard deviation of at least three biological replicates.

(B) Distribution of Oct4 expression in 5 replicate experiments described in Figure 7A-D. Experiment 3 is also shown in Figure 7C. Act1: 1ng/ml Activin A. A.F.U.: Arbitrary fluorescence units.

(C) Representative images of Id1+/+, Id1-/- and Id1-/- rescue chimaeras, cultured to the blastocyst stage and stained for LaminB1, GFP, Oct4 and Egr1. Egr1 expression was detected in a single cell of an Id1-/- chimaera (shown in Figure) across all stained blastocysts. Scale bars: 30μm.

(D) Representative images of Id1+/+, Id1-/- and Id1-/- rescue chimaeras, cultured to the blastocyst stage and stained for LaminB1, GFP and Gata6. Scale bar: 30μm.

(E) Representative images of Id1+/+, Id1-/- and Id1-/- rescue chimaeras, cultured to the blastocyst stage and stained for LaminB1, GFP and Cleaved Caspase3. An embryoid body (EB) section is included as a positive control. Scale bar: 30μm.

Table S2. List of primers used in this study, Related to STAR Methods.

qRT-PCR primer sequences		
**Description**	**Sequence**	**UPL Probe #**
Acta1 Forward	TGAAGCCTCACTTCCTACCC	81
Acta1 Reverse	CGTCGCACATGGTGTCTAGT	81
Acta2 Forward	CTCTCTTCCAGCCATCTTTCAT	58
Acta2 Reverse	TATAGGTGGTTTCGTGGATGC	58
Clstn3 Forward	TTGAGAGTGCTCGTCCTGTC	6
Clstn3 Reverse	GCCATTCATCATCACACACC	6
Cyp11a1 Forward	AAGTATGGCCCCATTTACAGG	104
Cyp11a1 Reverse	TGGGGTCCACGATGTAAACT	104
Dnmt3l Forward	AACCGACGGAGCATTGAA	34
Dnmt3l Reverse	AAACAAGGGGTGCCGAGT	34
Dppa3 Forward	GATAGGATGCACAACGATCCA	73
Dppa3 Reverse	AAAAATGCTTTTATTACAAATTTCTGG	73
E47 Forward	GTGGGCTCTGACAAGGAACT	79
E47 Reverse	ACAGGTAGCGGGAACATCAT	79
Epb4.9 Forward	TCATGATCTATGAGCCCCACT	79
Epb4.9 Reverse	GTGGATTTGGGTGACAGTGA	79
Egr1 Forward	CCTATGAGCACCTGACCACA	22
Egr1 Reverse	TCGTTTGGCTGGGATAACTC	22
Egr2 Forward	CTACCCGGTGGAAGACCTC	60
Egr2 Reverse	GTCAATGTTGATCATGCCATCT	60
Esrrb Forward	CGATTCATGAAATGCCTCAA	89
Esrrb Reverse	CCTCCTCGAACTCGGTCA	89
Fcgrt Forward	CCTCAAGACCCTGGAGAAGAT	40
Fcgrt Reverse	TATCCGAGGCCAGTTCACA	40
Fgf5 Forward	AAAACCTGGTGCACCCTAGA	29
Fgf5 Reverse	CATCACATTCCCGAATTAAGC	29
Fgfr2 Forward	CCTGCGGAGACAGGTAACAG	17
Fgfr2 Reverse	CGGGGTGTTGGAGTTCAT	17
Gata4 Forward	CCCCTCATTAAGCCTCAGC	77
Gata4 Reverse	CACCCTCGGCATTACGAC	77
Gata6 Forward	GGTCTCTACAGCAAGATGAATGG	40
Gata6 Reverse	TGGCACAGGACAGTCCAAG	40
Gli2 Forward	CCAGTGGCTCTTATGGACATC	26
Gli2 Reverse	GTAGGCCACAGGATTGATGG	26
Gtsf1l Forward	GGCTTTTCCTTTCGAGGTGT	18
Gtsf1l Reverse	TATGGATTCTGGCTCCATCC	18
Hopx Forward	ACCACGCTGTGCCTCATC	68
Hopx Reverse	GCGCTGCTTAAACCATTTCT	68
Id1 Forward	TCCTGCAGCATGTAATCGAC	78
Id1 Reverse	GGTCCCGACTTCAGACTCC	78

Klf4 Forward	CGGGAAGGGAGAAGACACT	62
Klf4 Reverse	GAGTTCCTCACGCCAACG	62
Kpna2 Forward	CTGCCCGACTTAACAGGTTC	56
Kpna2 Reverse	TTAGCTTTCCTGAGTTCCACATT	56
Krt17 Forward	AGGAGCTGGCCTACCTGAA	63
Krt17 Reverse	ACCTGGCCTCTCAGAGCAT	63
Lefty1 Forward	CTCAGTATGTGGCCCTGCTAC	76
Lefty1 Reverse	AACCTGCCTGCCACCTCT	76
Lefty2 Forward	GCCCTCATCGACTCTAGGC	97
Lefty2 Reverse	AGCTGCTGCCAGAAGTTCAC	97
Mras Forward	TGGGCCATCTTGGATGTT	51
Mras Reverse	CTGTGCGCATGTATTGTTCC	51
Nanog Forward	CCTCCAGCAGATGCAAGAA	25
Nanog Reverse	GCTTGCACTTCATCCTTTGG	25
Nr5a2 Forward	ATGGGAAGGAAGGGACAATC	22
Nr5a2 Reverse	TTGTTGAACGCGACTTCTGT	22
Oct4 Forward	GTTGGAGAAGGTGGAACCAA	95
Oct4 Reverse	CTCCTTCTGCAGGGCTTTC	95
Oct6 Forward	CTCAAGCCGCTGCTCAAC	25
Oct6 Reverse	CGCGATCTTGTCCAGGTT	25
Otx2 Forward	GACCCGGTACCCAGACATC	103
Otx2 Reverse	GCTCTTCGATTCTTAAACCATACC	103
Rex1 Forward	CAGCTCCTGCACACAGAAGA	16
Rex1 Reverse	ACTGATCCGCAAACACCTG	16
Rnf125 Forward	GAGGACTGAGATTTAAGGTGGTG	103
Rnf125 Reverse	GCAAGATCGGCAGAACCA	103
Sdha Forward	CAGTTCCACCCCACAGGTA	71
Sdha Reverse	TCTCCACGACACCCTTCTGT	71
Slc28a1 Forward	GCTGTTTGAGTGGATCAGCA	108
Slc28a1 Reverse	GACGATCAAGAAGCCGATGT	108
Sox1 Forward	GTGACATCTGCCCCCATC	60
Sox1 Reverse	GAGGCCAGTCTGGTGTCAG	60
Sox2 Forward	GTGTTTGCAAAAAGGGAAAAGT	34
Sox2 Reverse	TCTTTCTCCCAGCCCTAGTCT	34
Sox17 Forward	CACAACGCAGAGCTAAGCAA	97
Sox17 Reverse	CGCTTCTCTGCCAAGGTC	97
T Forward	ACTGGTCTAGCCTCGGAGTG	27
T Reverse	TTGCTCACAGACCAGAGACTG	27
Tbp Forward	GGGGAGCTGTGATGTGAAGT	97
Tbp Reverse	CCAGGAAATAATTCTGGCTCA	97
Tcea3 Forward	AGCTGAACAGTTGCCAGATG	42
Tcea3 Reverse	ACTGCCACTCCGATCCTG	42
Tcf15 Forward	GTGTAAGGACCGGAGGACAA	104
Tcf15 Reverse	GATGGCTAGATGGGTCCTTG	104
Tfcp2l1 Forward	GGGGACTACTCGGAGCATCT	53

Tfcp2l1 Reverse	TTCCGATCAGCTCCCTTG	53
Tfap2c Forward	CGCGGAAGAGTATGTTGTTG	62
Tfap2c Reverse	CGATCTTGATGGAGAAGGTCA	62
Trim2 Forward	GTGGACTCAAATGGGAACATC	5
Trim2 Reverse	TGATCCACTCCCATCAAAAAC	5
Trim36 Forward	CACCTCCCCTAGCATGGATA	64
Trim36 Reverse	TCGGGGTCAGTGAATTACG	64
Wnt8a Forward	ACTGCGGCTGTGACGAGT	75
Wnt8a Reverse	CCCGAACTCCACGTTGTC	75
Ywhaz Forward	TTACTTGGCCGAGGTTGCT	9
Ywhaz Reverse	TGCTGTGACTGGTCCACAAT	9

Southern blot probe amplification primer sequences

**Description**	**Sequence**
Nanog targeting probe forward	AAATTTATTTCTGGGCATGTGGTGAC
Nanog targeting probe reverse	GGATCCAGATTTCAGGATTGGAGG
Nanog multiple integration probe forward	TTCAGAAATCCCTTCCCTCG
Nanog multiple integration probe reverse	ATGCTAACTGCTTCTGCTGG
