# Supplementary material for: Id1 Stabilizes Epiblast Identity by Sensing Delays in Nodal Activation and Adjusting the Timing of Differentiation
Source: Dev Cell. 2019 Aug 19;50(4):462–477.e5. doi: 10.1016/j.devcel.2019.05.032 (PMC6706657; doi:10.1016/j.devcel.2019.05.032)
Supplement: Document S2. Article plus Supplemental Information [file mmc3.pdf]

Developmental Cell

Article

Id1 Stabilizes Epiblast Identity by Sensing Delays in Nodal Activation and Adjusting the Timing of Differentiation

Graphical Abstract

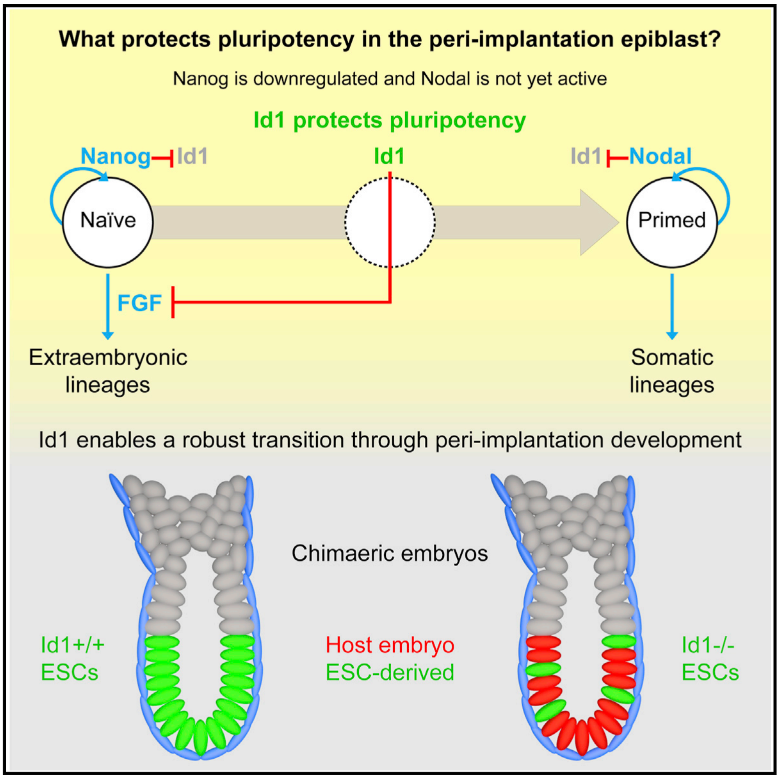

Authors

Mattias Malaguti, Rosa Portero Migueles, Guillaume Blin, Chia-Yi Lin, Sally Lowell

Correspondence

sally.lowell@ed.ac.uk

In Brief

Signals are often re-purposed as development proceeds. For example, FGF drives extraembryonic differentiation of pluripotent cells before the embryo implants, yet maintains somatic potency in their post-implantation descendants. Malaguti et al. show how pluripotent cells sense changes in their environment to ensure that FGF does not become activated prematurely.

Highlights

- Id1 acts as a “sensor” to detect when cells lack both Nanog and Nodal activity
- Id1 suppresses FGF in order to protect cells from aberrant differentiation
- Id1 protects pluripotency during transitions between naive and primed cell states
- Id1 is required for a robust transition through peri-implantation development.

Malaguti et al., 2019, Developmental Cell *50*, 462–477
August 19, 2019 © 2019 The Author(s). Published by Elsevier Inc.
https://doi.org/10.1016/j.devcel.2019.05.032

CellPress

Developmental Cell
Article

Id1 Stabilizes Epiblast Identity by Sensing Delays in Nodal Activation and Adjusting the Timing of Differentiation

Mattias Malaguti,[1] Rosa Portero Migueles,[1] Guillaume Blin,[1] Chia-Yi Lin,[1] and Sally Lowell[1,2,*]
[1]MRC Centre for Regenerative Medicine, Institute for Stem Cell Research, School of Biological Sciences, the University of Edinburgh, 5 Little France Drive, Edinburgh EH16 4UU, UK
[2]Lead Contact
*Correspondence: sally.lowell@ed.ac.uk
https://doi.org/10.1016/j.devcel.2019.05.032

SUMMARY

Controlling responsiveness to prevailing signals is critical for robust transitions between cell states during development. For example, fibroblast growth factor (FGF) drives naive pluripotent cells into extra-embryonic lineages before implantation but sustains pluripotency in primed cells of the post-implantation epiblast. Nanog supports pluripotency in naive cells, while Nodal supports pluripotency in primed cells, but the handover from Nanog to Nodal does not proceed seamlessly, opening up the risk of aberrant differentiation if FGF is activated before Nodal. Here, we report that Id1 acts as a sensor to detect delays in Nodal activation after the downregulation of Nanog. Id1 then suppresses FGF activity to delay differentiation. Accordingly, Id1 is not required for naive or primed pluripotency but rather stabilizes epiblast identity during the transition between these states. These findings help explain how development proceeds robustly in the face of imprecise signals and highlight the importance of mechanisms that stabilize cell identity during developmental transitions.

INTRODUCTION

Pluripotent cells in the early embryo choose their fate according to the signals they receive from their local environment (Arnold and Robertson, 2009). However, pluripotent cells are unlikely to respond passively to prevailing signals. Rather, the ability to respond to or ignore particular signals must be tightly coordinated with changes in differentiation potential in order to ensure that cell fate decisions are not misdirected by premature fluctuations in pro-differentiation cues.

Control over signal responsiveness becomes particularly important where the same signal is re-deployed to regulate successive cell fate restrictions. For example, fibroblast growth factor (FGF) drives naive pluripotent cells in the early embryo to differentiate into extraembryonic cell types (Chazaud et al., 2006; Hamilton and Brickman, 2014; Nichols et al., 2009; Yamanaka et al., 2010), whereas FGF helps to sustain pluripotency once pluripotent cells have transitioned into a "primed" state (Arnold and Robertson, 2009; Brons et al., 2007; Tesar et al., 2007). Therefore, for this transition to proceed successfully, the shift in FGF activity must somehow be timed to occur only after cells irreversibly commit to the primed epiblast state.

Nodal protects pluripotency in the primed epiblast of the post-implantation embryo (Camus et al., 2006; Mesnard et al., 2006) while Nanog protects pluripotency in the naive epiblast of the pre-implantation embryo (Mitsui et al., 2003). The handover between these two factors does not, however, appear to proceed seamlessly: some Nanog-negative epiblast cells lack Nodal activity in the late pre-implantation embryo (Granier et al., 2011). With neither Nanog nor Nodal available to sustain epiblast identity, these transiting epiblast cells would be in a precarious state, unless some other factor comes into play to protect them against the pro-endoderm effects of autocrine FGF (Chazaud et al., 2006; Hamilton and Brickman, 2014; Nichols et al., 2009; Yamanaka et al., 2010). This putative factor should have three key properties: the ability to sense low levels of Nodal activity, the ability to dampen FGF responsiveness, and the ability to protect pluripotent cells from differentiation.

A likely candidate is the BMP (Bone Morphogenic Protein) target gene *Id1*. *Id1* is sensitive to Nodal activity (Galvin et al., 2010) and is able to prevent differentiation of pluripotent cells (Ying et al., 2003; Zhang et al., 2010), but the details of when and how it operates remain unclear. It has been proposed that Id1 supports naive pluripotency by maintaining high levels of Nanog (Galvin-Burgess et al., 2013; Romero-Lanman et al., 2012; Ying et al., 2003). However, surprisingly, we report here that Id1 protein is absent from the embryonic day (E) 3.5 embryo and is only expressed in cells that have lost Nanog expression during peri-implantation development. This seems incompatible with the idea that BMP-Id1 maintains naive pluripotency but is consistent with idea that Id1 comes into play to protect epiblast identity after downregulation of Nanog.

Here, we report that Id1 stabilizes an epiblast identity specifically during the transition between naive and primed states. Id1 acts as a "sensor" to detect when cells have lost Nanog expression but have not yet acquired Nodal activity. Id1 then suppresses FGF in order to protect these cells from aberrant differentiation. Once a Nodal-responsive post-implantation epiblast state has been achieved, Nodal suppresses Id1 expression and so permits FGF activity to rise to help sustain pluripotency in newly configured primed epiblast cells.

 © 2019 The Author(s). Published by Elsevier Inc.
This is an open access article under the CC BY license (http://creativecommons.org/licenses/by/4.0/).

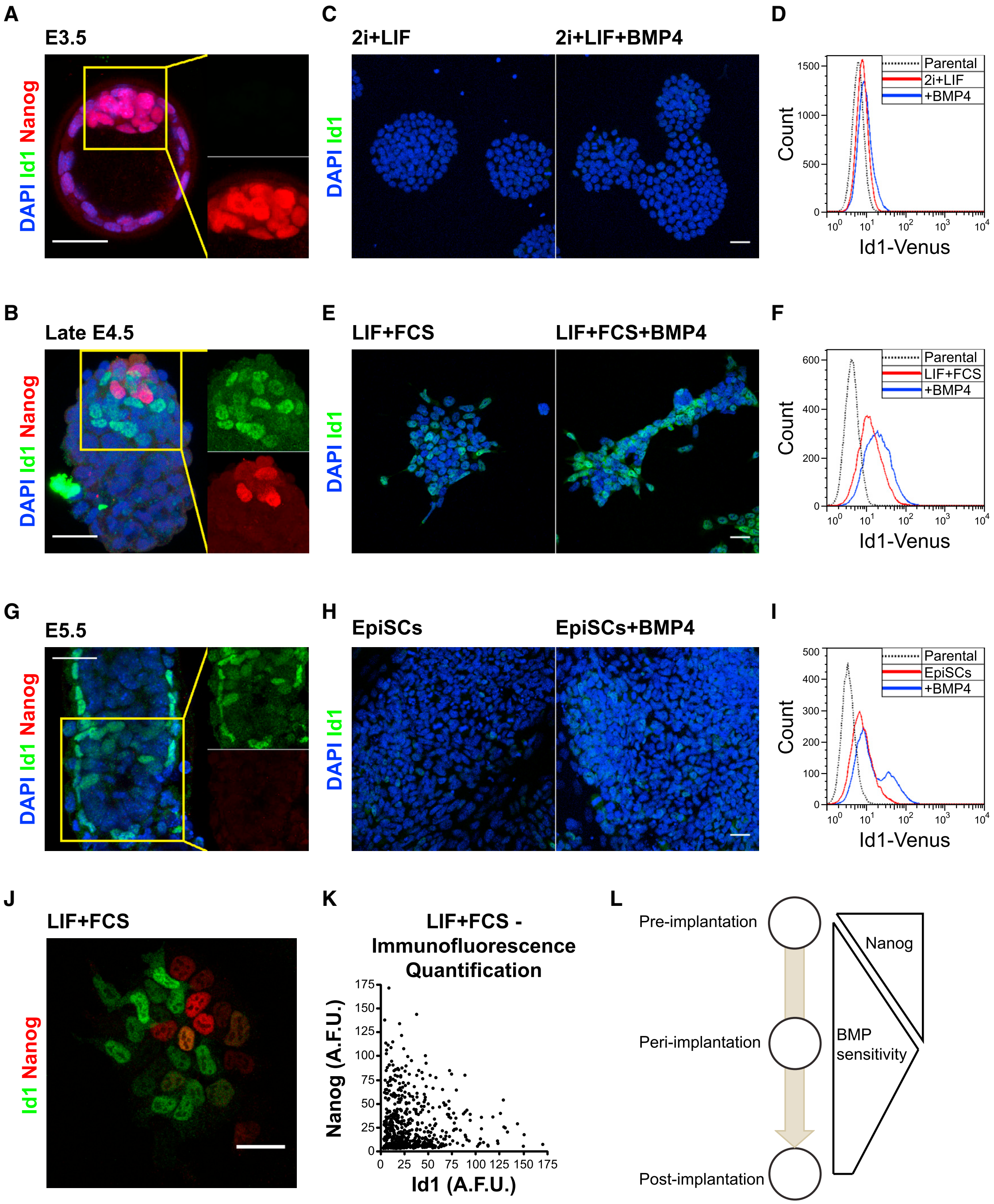

Figure 1. Pluripotent Cells Remain Resistant to BMP Signaling until Peri-implantation Stages of Development

(A) Immunofluorescent staining of E3.5 blastocyst for Nanog and the BMP target Id1.
(B) Immunofluorescent staining of late E4.5 blastocyst for Id1 and Nanog.
(C) Immunofluorescent Id1 staining of ESCs cultured in 2i + LIF, unstimulated or stimulated with 10 ng/mL BMP4 for 48 h.
(D) Flow cytometry analysis of Id1-Venus reporter ESCs cultured in 2i + LIF, unstimulated or stimulated with 10 ng/mL of BMP4 for 48 h.
(E) Immunofluorescent Id1 staining of ESCs cultured in LIF + FCS, unstimulated or stimulated with 10 ng/mL of BMP4 for 48 h.
(F) Flow cytometry analysis of Id1-Venus reporter ESCs cultured in LIF + FCS, unstimulated or stimulated with 10 ng/mL of BMP4 for 48 h.
(G) Immunofluorescent staining of E5.5 embryo for Id1 and Nanog.
(H) Immunofluorescent Id1 staining of EpiSCs, unstimulated or stimulated with 10 ng/mL of BMP4 for 48 h.
(I) Flow cytometry analysis of Id1-Venus reporter EpiSCs, unstimulated or stimulated with 10 ng/mL of BMP4 for 48 h.

(legend continued on next page)

We propose that this mechanism helps to coordinate changes in extrinsic and intrinsic information to ensure a robust transition through peri-implantation development.

RESULTS

Pluripotent Cells Remain Resistant to BMP Signaling until Peri-implantation Development

We examined whether pluripotent cells modulate responsiveness to prevailing signals as they proceed toward differentiation. We focused on BMP signaling because BMP suppresses differentiation of pluripotent cells in culture (Ying et al., 2003) and *in vivo* (Di-Gregorio et al., 2007). The BMP target gene *Id1* (Hollnagel et al., 1999) recapitulates the effects of BMP on pluripotent cells (Malaguti et al., 2013; Ying et al., 2003; Zhang et al., 2010) and provides a biologically relevant readout of BMP activity (Figures S1A–S1C).

Bmp4/7 and pSmad1 are readily detectable in pre-implantation embryos at E3.5 (Coucouvanis and Martin, 1999; Graham et al., 2014). However, to our surprise, we were unable to detect the product of the direct BMP target gene Id1 in E3.5 embryos (Figure 1A) or in early E4.5 embryos (data not shown). We then examined embryos after E4.5, at the latest stage obtainable before the embryo implants. These embryos contain a subpopulation of Id1+ cells scattered throughout the epiblast in a salt-and-pepper distribution (Figure 1B). This suggests that patterning of Id1 is unlikely to be explained only by exposure to exogenous BMP ligands (because these ligands are diffusible and so unlikely to adopt a salt-and-pepper distribution) and instead might reflect cell-cell variability in BMP responsiveness.

In order to test this, we examined pluripotent cells in culture, where we could stimulate cells with BMP4. We first examined cells in 2i + LIF culture, which supports a stage of pluripotency equivalent to that of the early E4.5 blastocyst (Boroviak et al., 2015). We were unable to detect Id1 protein even after stimulating 2i + LIF cells with high doses (10 ng/mL) of BMP4 (Figure 1C). These findings were confirmed using cells in which an Id1-Venus fusion was expressed from the *Id1* locus (Figures 1D and S1D–S1G) (Malaguti et al., 2013; Nam and Benezra, 2009).

We then examined embryonic stem cells (ESCs) in LIF (leukemia inhibitory factor) + fetal calf serum (FCS), a culture condition that supports a mixture of naive and primed cells (Nichols and Smith, 2009). We could detect Id1 protein in some cells, although a subpopulation remained Id1 negative even when stimulated with BMP4 (Figures 1E and 1F), in keeping with reports that naive cells do not activate Id1 in response to BMP (Gomes Fernandes et al., 2016).

We then examined post-implantation embryos and epiblast stem cells (EpiSCs) (Brons et al., 2007; Tesar et al., 2007). As expected, the E5.5 epiblast expresses Id1 in cells close to the extraembryonic ectoderm, a source of BMP signals (Arnold and Robertson, 2009) (Figures 1G and S1H). EpiSC stimulated with BMP4 express moderate levels of Id1 in a minor subset of cells (Figures 1H and 1I), indicating that BMP responsiveness decreases as pluripotent cells reach a primed state. This transient window of Id1 expression at the onset of the transition between naive and primed pluripotency can be recapitulated *in vitro* in epiblast-like cell (EpiLC) differentiation (Figure S1J).

Some epiblast cells in the late E4.5 embryo downregulate the naive determinant Nanog to prepare for the transition to a primed state (Xenopoulos et al., 2015). We detect Id1 exclusively within these Nanog-low cells (Figure 1B). Similarly, Id1 is expressed predominantly in Nanog-low cells in LIF + FCS (Figures 1J and 1K).

We conclude that pluripotent cells modulate responsiveness to BMP4 over time. They become most responsive as they enter a transition phase between naive and primed pluripotency, corresponding to a stage of peri-implantation development after downregulation of Nanog but before establishment of a primed pluripotent state (Figure 1L).

Id1 Predicts the Probability of Differentiating after Downregulation of Nanog

It is surprising that Id1, which maintains pluripotency of ESCs (Ying and Smith, 2003; Zhang et al., 2010), is not expressed in Nanog-high cells *in vivo* or in culture (Figures 1B, 1J, and 1K). Could it instead be protecting epiblast identity during the transition from naive to primed states?

The transition to a primed state is initiated by downregulation of Nanog in concert with other components of the naive gene regulatory network (GRN) (Kalkan et al., 2017). However, loss of Nanog does not commit cells to undergo this transition: some Nanog-low cells resist differentiation and revert back to a Nanog-high state (Chambers et al., 2007; Kalmar et al., 2009). We asked whether Id1 identifies those cells that resist differentiation after loss of Nanog.

We generated a dual-reporter ESC line, which expresses an Id1-Venus fusion protein from the endogenous *Id1* locus (Malaguti et al., 2013; Nam and Benezra, 2009), and a Nanog-tagRFP fusion protein from the endogenous *Nanog* locus (Figures 2A and S2A–S2E). We first confirmed that Nanog and Id1 tend to mark different subpopulations in LIF + FCS (Figure 2B). We then sorted three populations of cells from LIF + FCS: Nanog-high (NR-HI IdV-LO), Id1-high Nanog-low (IdV-HI NR-LO), and Id1-low Nanog-low cells (IdV-LO NR-LO) (Figures 2C and S2F).

As expected (Festuccia et al., 2012), transcriptomes differed between Nanog-high cells and Nanog-low cells. In contrast, within the Nanog-low compartment, transcriptomes of Id1-high and Id1-low cells were almost indistinguishable (Figures 2D and 2E; Table S1).

Id1 is not a transcriptional regulator: it acts by controlling the activity of a range of proteins (Norton, 2000; Roberts et al., 2001; Yates et al., 1999), so it seemed plausible that Id1-high cells may be more resistant to differentiation than Id1-low cells despite their similar transcriptomes.

(J) Immunofluorescent staining of ESCs cultured in LIF + FCS for Id1 and Nanog.

(K) Quantification of Id1 and Nanog immunofluorescent signal in single ESCs cultured in LIF + FCS.

(L) Diagram illustrating how BMP sensitivity increases around the time of implantation, as Nanog is being lost, and decreases following implantation.

Scale bars, 30 μm.

See also Figure S1.

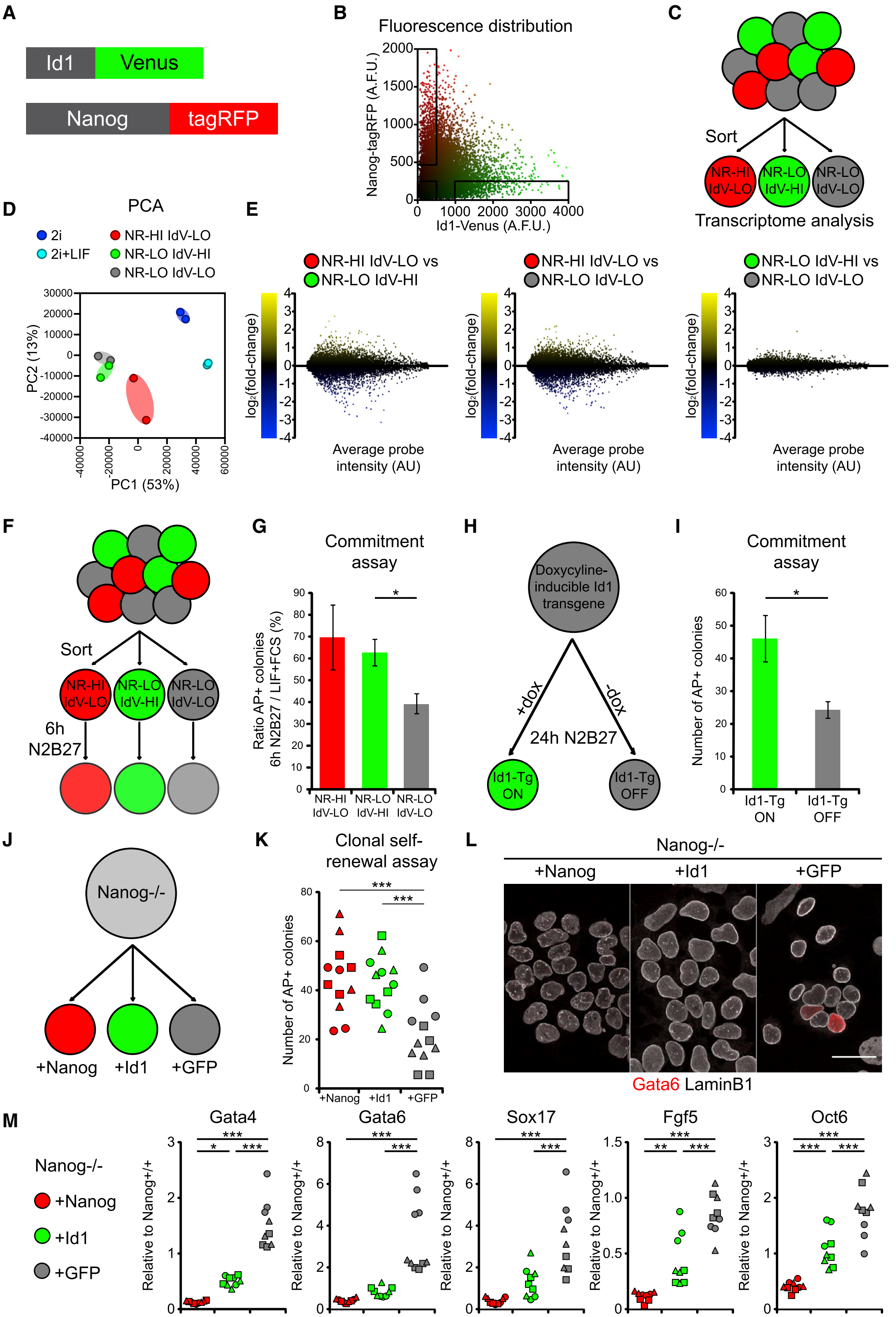

(legend on next page)

We tested the sorted subpopulations for resistance to differentiation. We challenged cells with differentiation medium (N2B27) for 6 h and then returned them to self-renewal conditions at clonal density to assess how many cells remained undifferentiated. We also plated sorted cells directly into self-renewal conditions at clonal density to measure the number of undifferentiated cells in each starting population. We combined these data to establish the proportion of undifferentiated cells that resist differentiation during the 6 h challenge (Figure 2F).

This reveals that Id1-high cells resist differentiation more effectively than Id1-low cells: the majority (62% ± 6%) of IdV-HI NR-LO cells resisted differentiation, as did the majority (69% ± 15%) of NR-HI IdV-LO cells. Only a minority (39% ± 5%) of IdV-LO NR-LO cells were able to resist differentiation (Figure 2G).

Although there is low residual expression of Nanog and other naive pluripotency transcription factors within our "Nanog-low" sorted subpopulations (Figures S2F and S2G), this cannot explain our findings because there was no difference in expression of these factors between Id1-high and Id1-low cells (Figures S2G and S2H), nor was there any difference in the number of colony-forming cells prior to the differentiation challenge (Figure S2I), indicating that there are no functional differences in naive transcription factor activity between the two populations.

We conclude that Id1 identifies a subpopulation of Nanog-low cells that resist differentiation independently of the activity of the naive pluripotency GRN.

Id1 Protects Pluripotent Cells from Differentiation in the Absence of Nanog

Having seen that Id1 correlates with resistance to differentiation after downregulation of Nanog, we asked whether Id1 is capable of suppressing differentiation after downregulation of Nanog. We made use of an ESC line containing a doxycycline-inducible *Id1* transgene (Malaguti et al., 2013). We placed these cells in N2B27 for 24 h, a time frame that is sufficient to downregulate members of the naive GRN (Kalkan et al., 2017), in the presence or absence of doxycycline. We then replated the cells clonally in self-renewal conditions (Figure 2H). We find that forced expression of Id1 during this time window in which the naive GRN is dismantled increases the number of cells that resist differentiation (Figure 2I).

If Id1 protects pluripotent cells from differentiation in the absence of Nanog, then it should be able to rescue the spontaneous differentiation phenotype of Nanog-null cells in LIF + FCS (Chambers et al., 2007). Forced expression of Id1 restores the colony-forming ability of Nanog-null cells to a similar extent to forced expression of Nanog itself (Figures 2J and 2K) and reduces the expression of markers of primitive endoderm (*Gata4*, *Gata6*, and *Sox17*) and primed epiblast (*Fgf5* and *Oct6*, also known as *Pou3f1*) (Figures 2L and 2M).

These data suggest that Id1 is responsible for protecting pluripotent cells from differentiation after downregulation of Nanog.

A Coordinated Shift in BMP and FGF and Nodal Responsiveness after Downregulation of Nanog

We looked for transcriptional changes that might explain why IdV-LO NR-LO cells are more susceptible to differentiation than IdV-HI NR-LO cells.

Compared with IdV-HI NR-LO cells, only six protein-coding genes were enriched in IdV-LO NR-LO cells (Figures 3A and 3B). Only two of these, *Egr1* and *Lefty1*, were confirmed by qRT-PCR to be differentially expressed (Figures 3C and S3A). *Egr1* and *Lefty1* are readouts of the FGF and Nodal signaling pathways, respectively, and these are the two pathways that sustain pluripotency in primed pluripotent cells (Brons et al., 2007; Camus et al., 2006; Tesar et al., 2007). We confirmed

Figure 2. Id1 Protects Pluripotent Cells from Differentiation in the Absence of Nanog

(A) Diagrammatic structure of Id1-Venus Nanog-tagRFP double reporter ESCs.

(B) Flow cytometry of Id1-Venus Nanog-tagRFP cells cultured in LIF + FCS confirm that high levels of Id1 expression are observed predominantly in Nanog-low cells. Gates used for sorting experiments are displayed.

(C) Sorting strategy for downstream transcriptome analysis of LIF + FCS cultures.

(D) PCA of the sorted subpopulations, 2i and 2i + LIF cultures.

(E) Pairwise transcriptomic comparisons of the three sorted subpopulations.

(F) Experimental strategy. Id1-Venus Nanog-tagRFP ESCs cultured in LIF + FCS were sorted into three subpopulations then assayed for their ability to form AP+ colonies when plated at clonal density immediately after sorting and after 6-h N2B27 culture.

(G) Proportion of cells capable of resisting differentiation after 6-h N2B27 culture. Number of AP+ colonies obtained after replating 6-h N2B27 cultures in LIF + FCS, divided by number of AP+ colonies obtained after replating cells in LIF + FCS immediately after sorting. Plating density: 1,000 cells/9 cm dish. Data are represented as mean ± SEM of three independent experiments.

(H) Experimental strategy for Id1 gain-of-function experiment. ESCs carrying a doxycycline-inducible *Id1* transgene were transferred from LIF + FCS culture to N2B27 for 24 h, in the presence or absence of 1 μg/mL doxycycline. The cells were assayed for their ability to form AP+ colonies when replated at clonal density in LIF + FCS.

(I) Number of AP+ colonies obtained after replating cells as described in (H). Plating density: 100 cells/well of a 6-well plate. Data are represented as mean ± SEM of five independent experiments.

(J) Diagram of rescue of Nanog$^{-/-}$ cells. Clonal cell lines were generated to stably express *Nanog* (positive control), *Id1,* or *GFP* (negative control).

(K) Number of undifferentiated AP+ colonies obtained upon plating Nanog-rescue cells in LIF + FCS. Each shape represents a different clonal line. Plating density: 100 cells/well of a 6-well plate.

(L) Immunofluorescent staining of Nanog-rescue cells cultured in LIF + FCS for Gata6 and LaminB1. Scale bar, 30 μm.

(M) qRT-PCR analysis of Nanog-rescue cells cultured in LIF + FCS. Each shape represents a different clonal line.

Statistical analyses: for comparison of two samples: two-tailed unpaired Student's t test; for comparison of three samples: one-way ANOVA followed by Tukey's multiple comparison test. *$p < 0.05$, **$p < 0.01$, ***$p < 0.001$.

NR, Nanog-tagRFP; IdV, Id1-Venus; HI, high; LO, low; AP, alkaline phosphatase; dox, doxycycline; Tg, transgene.

See also Figure S2 and Table S1.

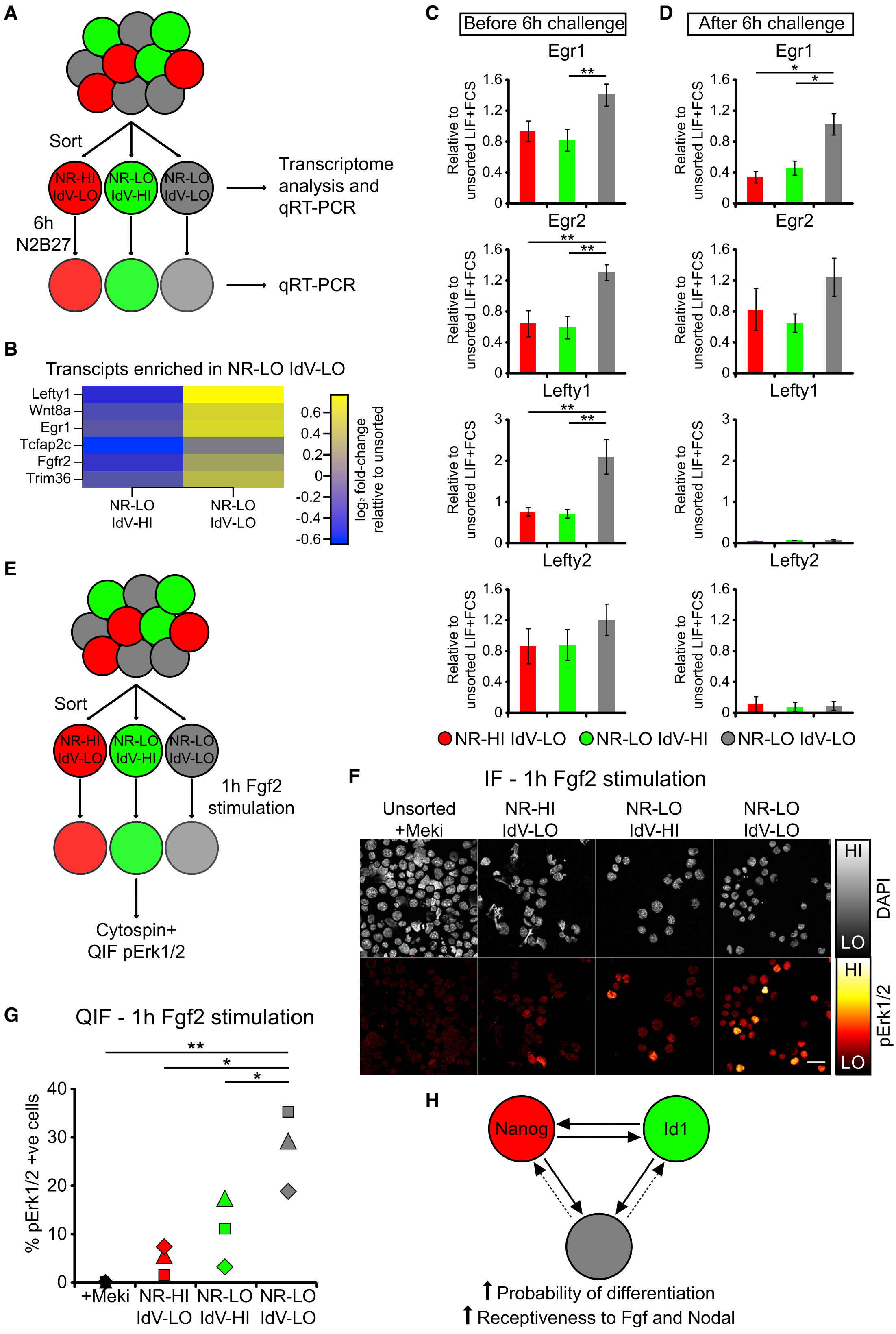

(legend on next page)

that the related FGF target gene *Egr2*, although not the related Nodal target gene *Lefty2*, is also enriched in IdV-LO NR-LO cells (Figure 3C). Id1 rescue of Nanog-null cells also correlates with reduced expression of *Egr1* (Figure S3B).

The FGF target gene *Egr1* (although not the Nodal targets genes *Lefty1* or *Lefty2*) remained enriched in Id1-low cells after a 6 h challenge with differentiation media, which is the time at which Id1-high cells display their relative resistance to differentiation (Figure 3D). Intriguingly, we do not observe differences in expression of naive or primed pluripotency markers between IdV-LO NR-LO and IdV-HI NR-LO cells at this time point (Figures S3C and S3D), suggesting that differences in FGF sensitivity and response can predict resistance to differentiation prior to overt changes in pluripotency marker expression.

Having observed an increase in FGF target gene expression in IdV-LO NR-LO cells, we next asked whether these cells are more responsive to acute stimulation with exogenous Fgf2 than Id1-high cells (Figure 3E). We find that a higher proportion of IdV-LO NR-LO cells respond to Fgf2 stimulation by phosphorylating Erk1/2 (a direct readout of FGF activity) than Id1-high or Nanog-high cells (Figures 3F and 3G).

These data suggest that there is a coordinated shift in signal responsiveness within the Nanog-low compartment, with cells becoming more responsive to FGF and Nodal signaling, as they lose Id1 expression. It is the increase in FGF responsiveness that best correlates with a higher probability of differentiating (model shown in Figure 3H).

Id1 Is Responsible for Suppressing Differentiation within the Nanog-Low Compartment

We next asked how differentiation is suppressed within Id1-high cells. Eight genes are enriched in Id1-high cells (Figure S4), including Id1 itself. Id1 has previously been reported to block differentiation of naive and primed pluripotent cells (Aloia et al., 2015; Malaguti et al., 2013; Romero-Lanman et al., 2012; Ying et al., 2003; Zhang et al., 2010), but a role during the transition between these two states has not been explored.

Id1-null ESCs have impaired clonogenic potential and display reduced levels of Nanog and increased levels of the primed pluripotency marker Oct6 (Figures 4A–4C). These phenotypes can be rescued by placing cells into 2i + LIF culture conditions in order to maintain uniform high levels of Nanog (Figures 4D–4F) or by addition of a Mek inhibitor (PD0325901) to LIF + FCS cultures in order to suppress FGF activity (Figures 4G–4I).

These data suggest that Id1 is dispensable within naive (Nanog-high) pluripotent cells but that it protects cells from differentiating after downregulation of Nanog.

Id1 Dampens FGF Responsiveness

We next asked whether Id1 is responsible for suppressing FGF activity. Id1-null cells display increased expression of the FGF target gene Egr1, and this can be reversed by restoring Id1 expression (Figures 5A–5C). Nodal activity is also dampened in Id1-high cells (Figures 3B and 3C), but Id1-null cells do not have increased expression of the Nodal target gene *Lefty1* (Figure 5D), suggesting that Nodal signaling may regulate, rather than be regulated by, Id1 expression.

Egr1 is not only a passive readout of FGF activity; it also mediates the effects of FGF on pluripotent cells (Galonska et al., 2015). Egr1 is correlated with and controlled by Id1 in our experiments (Figures 3B–3D, 5A–5C, and S3B), so we asked how Id1 regulates Egr1.

E2A homodimers directly regulate Egr1 in pro-B cells (Lin et al., 2010), and E2A activity is repressed by Id1 (Massari and Murre, 2000). We therefore first considered E2A as a likely candidate for mediating the effects of Id1 on Egr1. However, this does not seem to be the case: *Egr1* does not respond to experimental activation of E2A homodimers in ESCs (Figures S5A and S5B).

We have previously identified Tcf15 as an Id-regulated pro-differentiation factor in ESCs (Davies et al., 2013). Transcriptome analysis of Tcf15-responsive genes indicates that Tcf15 upregulates *Egr1* (Davies et al., 2013). Using Tcf15-null cells, we find that Tcf15 is required for maximal *Egr1* expression after downregulation of Nanog (Figures 5E and 5F). These data are consistent with the idea that Id1 suppresses Egr1 expression through suppression of Tcf15 activity.

Taken together, our data suggest that Id1 orchestrates a coordinated shift in growth factor responsiveness and differentiation.

Figure 3. A Coordinated Shift in BMP, FGF, and Nodal Responsiveness after Downregulation of Nanog

(A) Experimental strategy. ESCs cultured in LIF + FCS were sorted into three subpopulations based on Id1-Venus and Nanog-tagRFP. Samples were taken for gene expression analysis immediately after sorting and after 6 h culture in N2B27 differentiation medium.

(B) Heatmap of transcripts significantly enriched in NR-LO IdV-LO relative to NR-LO IdV-HI subpopulations. Significance was defined as $\log_2$(fold-change) > 0.5, p value adjusted for multiple testing correction < 0.5.

(C) qRT-PCR analysis of sorted subpopulations before the 6-h N2B27 differentiation challenge. Data are represented as mean ± SEM of seven independent experiments.

(D) qRT-PCR analysis of sorted subpopulations after the 6-h N2B27 differentiation challenge. Data are represented as mean ± SEM of three independent experiments.

(E) Experimental strategy for acute Fgf stimulation of sorted subpopulations. Following sorting, cells were cultured in N2B27+10 ng/mL Fgf2 in suspension, then cytospun and stained for pErk1/2 expression.

(F) Immunofluorescent staining of sorted subpopulations for pErk1/2. Scale bar, 30 μm.

(G) Percentage of pErk1/2-positive cells following 1 h Fgf2 stimulation of sorted subpopulations, calculated by quantitative immunofluorescence of cells shown in (F). Samples from the same sort are indicated with the same shape.

(H) Model: once Nanog is lost from pluripotent cells, Id1 expression is associated with resistance to differentiation and lower expression of Fgf and Nodal signaling targets.

Statistical analyses of qRT-PCR and QIF data were performed using a one-way ANOVA followed by Tukey's multiple comparison test. *p < 0.05, **p < 0.01, ***p < 0.001. Methods for statistical analysis of transcriptomic data are described in the STAR Methods section.

NR, Nanog-tagRFP; IdV, Id1-Venus; HI, high; LO, low, Meki: Mek inhibitor (1 μM PD0325901); QIF, quantitative immunofluorescence; +ve, positive. See also Figure S3 and Table S1.

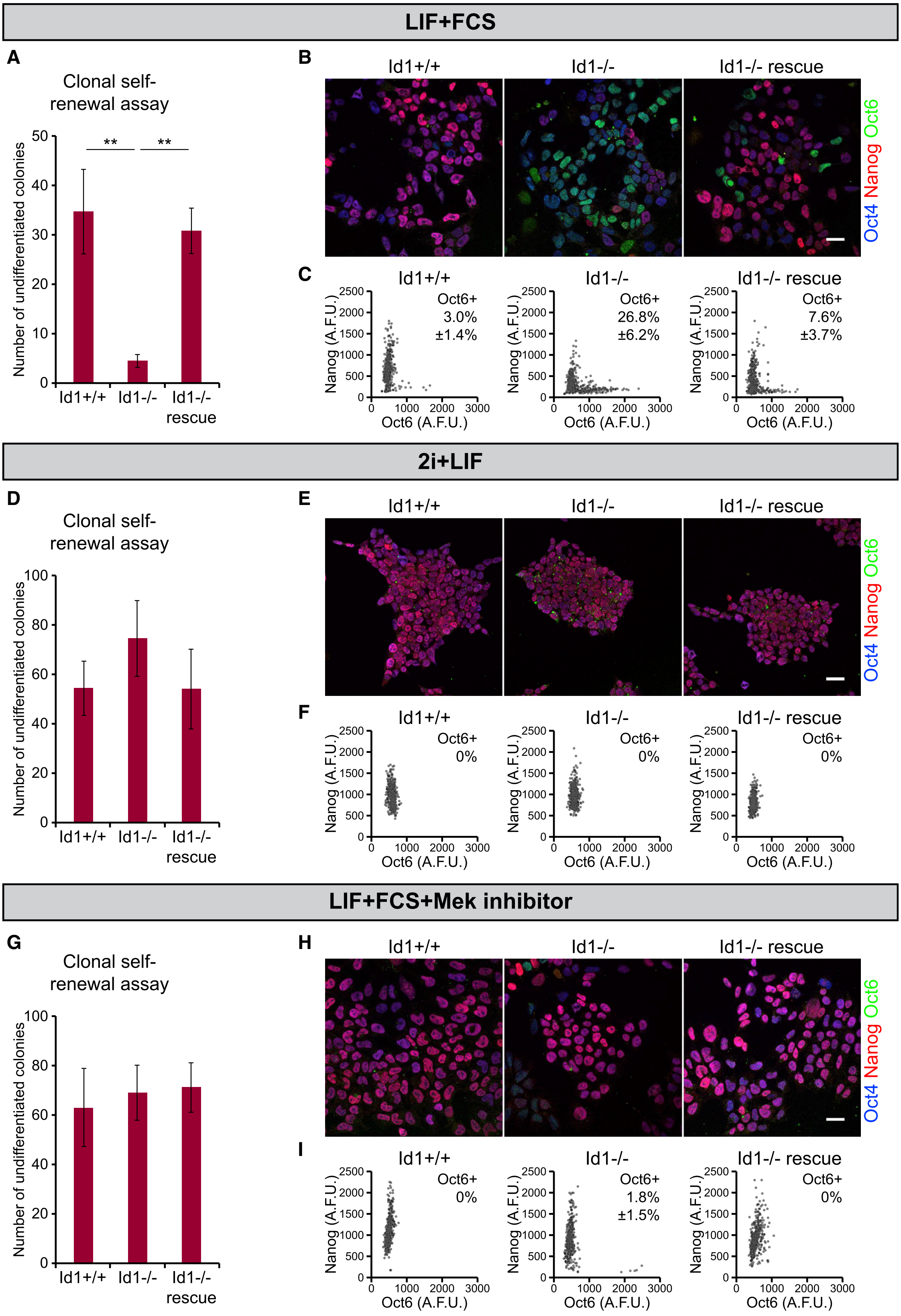

(legend on next page)

Id1 Acts As a "Sensor" of Nodal Activity

We report above that there is a transient peak of Id1 protein expression during the transition from naive to primed epiblast states resulting from changes in "responsiveness" to BMP rather than changes in "exposure" to BMP (Figures 1C–1F, 1H, and 1I). We asked what is responsible for suppressing Id1 in naive cells.

Nanog is able to repress Id1 expression (Figures S6A and S6B; Festuccia et al., 2012; Suzuki et al., 2006) and Id1 is derepressed in Nanog-null cells (Figures 6A and 6B). We examined Nanog-null ESCs in which the naive subpopulation can be identified via a fluorescent reporter targeted to the Nanog locus (Chambers et al., 2007). This confirmed that there is an overall increase in Id1 expression, although Id1 remains repressed in a subset of Nanog-null cells (Figures 6C and 6D). We conclude that Nanog contributes to, but is not solely responsible for, repression of Id1.

Nodal signaling is also able to repress Id1 expression (Galvin et al., 2010; Galvin-Burgess et al., 2013). The Nodal target gene *Lefty1* is enriched in Id1-low cells in LIF + FCS (Figures 3B and 3C) yet is not affected in Id1-null cells (Figure 5D), supporting the idea that Nodal signaling acts upstream rather than downstream of Id1.

In 2i + LIF, BMP4 is usually unable to upregulate Id1 (Figures 1C and 1D), but after addition of the Nodal inhibitor SB431542, almost all cells switch on Id1 in response to BMP4 (Figures 6E–6G). Similarly, in LIF + FCS cultures, SB431542 derepresses Id1, with the strongest increase observed within the Nanog-low subpopulation (Figures 6H–6J). SB431542 also permits BMP4-induced Id1 expression in EpiSCs (Figures 6K–6M). In keeping with these observations, treatment of 2i + LIF and LIF + FCS cultures with the Nodal agonist Activin A inhibits Id1 induction by BMP4 (Figures S6C and S6D). These data suggest that Nodal is the primary factor responsible for dampening Id1 expression in primed cells.

We conclude that Nanog and Nodal repress Id1 within naive cells and that Nodal also dampens Id1 expression within primed cells. This explains how Id1 can act as a "sensor" of Nodal activity after downregulation of Nanog (Figure 6N).

Id1 Is Required for a Robust Transition from a Naive to a Primed Epiblast State *In Vivo*

Our findings suggest that Id1 protects epiblast cells from pro-differentiation cues from the time they lose Nanog expression through to the time that Nodal signaling begins to sustain them in a primed state.

In keeping with this model, Id1 is dispensable under optimized differentiation conditions *in vitro*, where inappropriate pro-differentiation signals are eliminated (Figure S7A). We predict that Id1 should become important under sup-optimal signaling conditions such as those in the peri-implantation embryo where, for example, Nodal becomes activated in only a subset of Nanog-low cells (Granier et al., 2011). We devised an *in vitro* assay to mimic these conditions. We cultured pluripotent cells in basal media (N2B27) in order to allow cells to initiate exit from naive pluripotency in the absence of exogenous cues. After 48 h, we provided cells with low levels (1 ng/mL) of the Nodal agonist Activin A to approximate the incomplete activation of Nodal *in vivo* (Figure 7A). We used Oct4 (Pou5f1) to indicate the ability of these cells to retain an epiblast identity.

When this assay is carried out in the absence of BMP, only around half of cells retained Oct4 expression (Figures 7B–7D and S7B: note bimodal distribution of Oct4 in Figures 7C and S7B). Exposing cells to BMP in order to activate Id1 during the first 48 h increased the robustness with which cells progress through this transition, with the majority of cells maintaining Oct4 expression (Figures 7B–7D and S7B: note unimodal distribution of Oct4 in Figures 7C and S7B).

These results go some way toward supporting the hypothesis that BMP-Id1 helps to protect pluripotent cells from suboptimal signaling conditions. However, our *in vitro* assay falls far short of capturing the complexities of the dynamic signaling environment of the peri-implantation embryo. We therefore turned to an *in vivo* assay system.

We examined the efficiency with which Id1-null cells can persist throughout implantation and contribute to the post-implantation epiblast in aggregation chimeras (Figure 7E). Cells, which differentiate aberrantly or activate FGF prematurely during this process, are eliminated by cell competition (Clavería et al., 2013; Díaz-Díaz et al., 2017; Sancho et al., 2013).

Id1-null cells are able to contribute to the post-implantation epiblast: 57% of embryos contained at least some Id1-null ESCs, which is comparable to results from wild-type cells (67% of embryos) and Id1-rescue cells (54% of embryos) (Figures 7F and 7G). However, the degree of contribution was lower for Id1-null cells (30% high contribution) than wild-type (74% high contribution) or Id1-rescue cells (53% high contribution) (Figures 7F and 7G). Quantifying the number of ESCs that contribute to the post-implantation epiblast confirmed that Id1-null cells contribute to the epiblast less robustly than wild-type or Id1-rescue cells (Figures 7F–7H).

Figure 4. Id1 Is Responsible for Suppressing Differentiation within the Nanog-Low Compartment

(A) Clonal self-renewal assays of wild-type, Id1-null, and Id1-rescue (Id1-null cells stably expressing an Id1 transgene) ESCs cultured in LIF + FCS.

(B) Immunofluorescent staining of wild-type, Id1-null, and Id1-rescue ESCs cultured in LIF + FCS for Nanog, Oct4, and Oct6.

(C) Quantification of the IF data in (B).

(D) Clonal self-renewal assays of wild-type, Id1-null and Id1-rescue ESCs cultured in 2i + LIF.

(E) Immunofluorescent staining of wild-type, Id1-null, and Id1-rescue ESCs cultured in 2i + LIF for Nanog, Oct4, and Oct6.

(F) Quantification of the IF data in (E).

(G) Clonal self-renewal assays of wild-type, Id1-null, and Id1-rescue ESCs cultured in LIF + FCS + 1 μM PD0325901 (a Mek inhibitor).

(H) Immunofluorescent staining of wild-type, Id1-null, and Id1-rescue ESCs cultured in LIF + FCS + 1 μM PD0325901 for Nanog, Oct4, and Oct6.

(I) Quantification of the IF data in (H).

All data are represented as mean ± standard deviation of three independent experiments. Statistical analyses were performed using a one-way ANOVA followed by Tukey's multiple comparison test. **$p < 0.01$. Plating density was 100 cells/well of a 6-well plate for clonal assays. Note that the height of the y axes differs between (A), (D), and (G). Immunofluorescence scale bars: 30 μm. See also Figure S4.

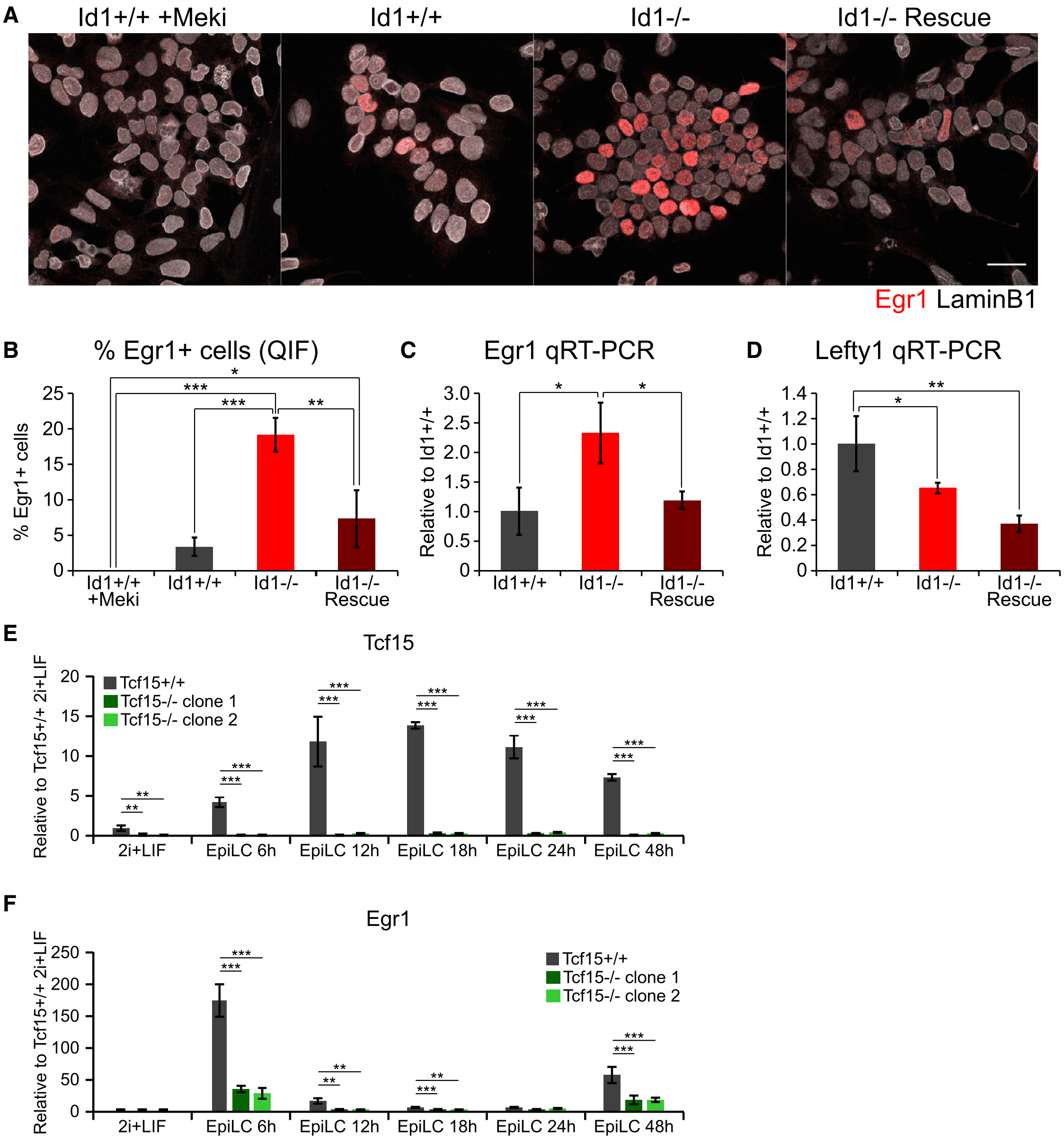

Figure 5. Id1 Dampens FGF Responsiveness by Modulating the Activity of Tcf15

(A) Immunofluorescent staining for Egr1 and LaminB1 in wild-type, Id1-null, Id1-rescue ESCs cultured in LIF + FCS. Wild-type cells cultured in LIF + FCS + 1 μM PD0325901 (+Meki) are included as a negative control. Scale bar, 30 μm.

(B) Quantification of the IF data in (A).

(C) qRT-PCR for the Fgf target *Egr1* in wild-type, Id1-null, and Id1-rescue ESCs cultured in LIF + FCS.

(D) qRT-PCR for the Nodal target *Lefty1* in wild-type, Id1-null, and Id1-rescue ESCs cultured in LIF + FCS.

(E) qRT-PCR for *Tcf15* in wild-type and two Tcf15-null clonal cell lines during 2i + LIF to EpiLC differentiation.

(F) qRT-PCR for *Egr1* in the samples described in (E).

Data are presented as mean ± standard deviation of three independent experiments. Statistical analyses were performed using a one-way ANOVA followed by Tukey's multiple comparison test. *p < 0.05, **p < 0.01, ***p < 0.001. See also Figure S5.

In contrast, Id1-null ESCs contribute efficiently to the pre-implantation pluripotent epiblast, displaying no sign of premature differentiation or cell death (Figures S7C–S7E). This confirms that Id1 is not required in naive pluripotent cells but becomes important during peri-implantation development.

We conclude that Id1 is required for a robust transition from the pre-implantation to the post-implantation epiblast *in vivo*.

DISCUSSION

Coordinating Signaling with Differentiation during Transitions between Cell States

There has been much progress in understanding the signals and transcription factors that maintain naive and primed pluripotent cell states (Betschinger et al., 2013; Buecker et al., 2014;

CellPress

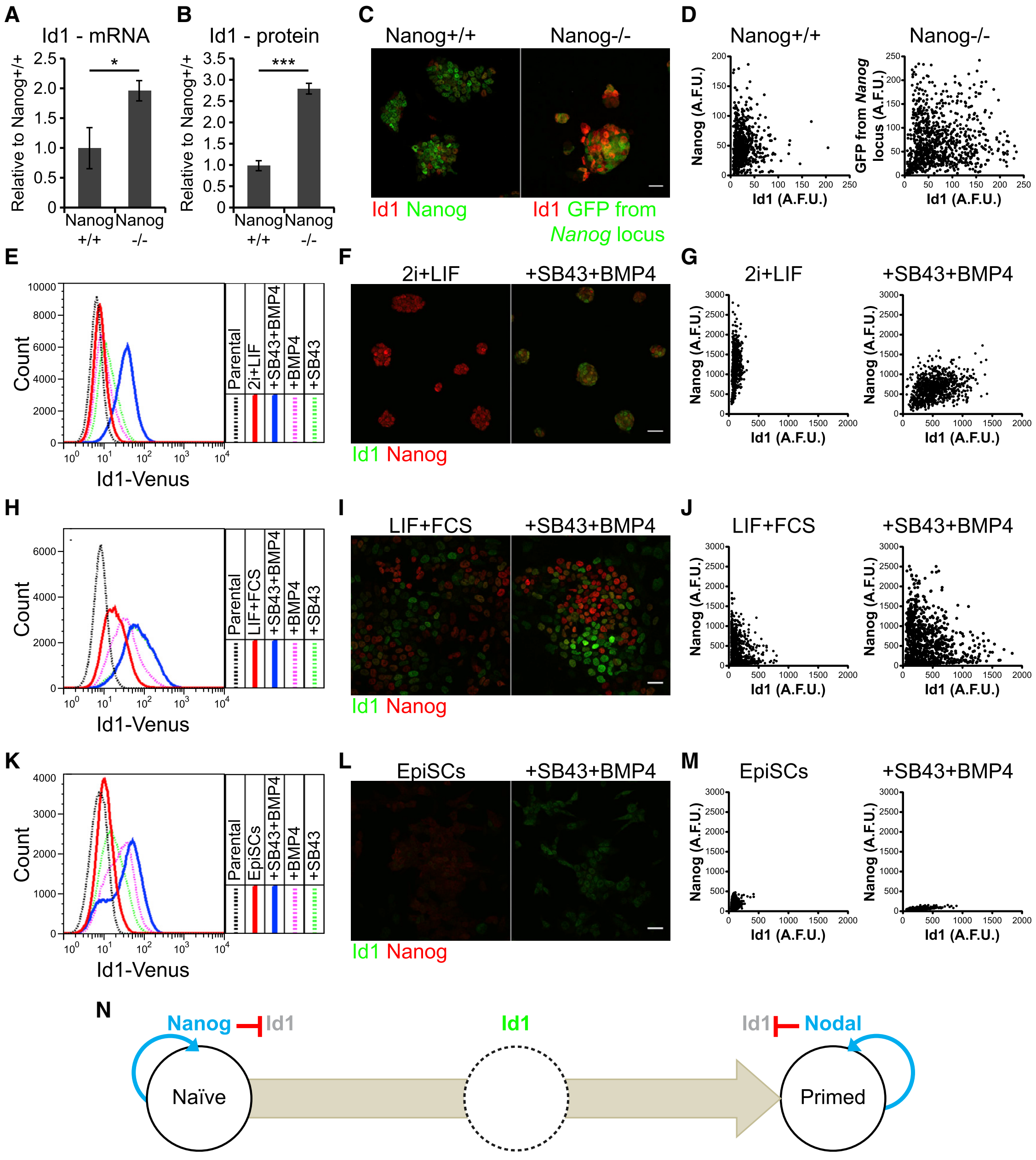

Figure 6. Dynamic Regulation of Id1 Expression during the Transition from Naive to Primed States

(A) qRT-PCR for *Id1* in wild-type and Nanog-null ESCs cultured in LIF + FCS. Data are represented as mean ± standard deviation of three independent experiments.

(B) Median Id1 protein expression following immunofluorescence quantification of Id1 staining in wild-type and Nanog-null ESCs cultured in LIF + FCS. Data are represented as mean ± standard deviation of three independent experiments.

(C) Immunofluorescence for Id1 and Nanog or GFP in wild-type ESCs and in Nanog-null ESCs harboring a *GFP* transgene under the control of the *Nanog* promoter.

(D) Quantification of Id1 and Nanog or GFP immunofluorescent signal in single wild-type or Nanog-null ESCs cultured in LIF + FCS.

(E) Flow cytometry analysis of Id1-Venus ESCs cultured in 2i + LIF with or without stimulation with 10 ng/mL BMP4 and/or 10 μM of the Nodal inhibitor SB431542 for 48 h.

(F) Immunofluorescence for Id1 and Nanog in wild-type ESCs cultured in 2i + LIF with or without 48 -h stimulation with 10 ng/mL BMP4 and 10 μM SB431542.

(G) Quantification of immunofluorescence signal for the cells in (F).

(H) Flow cytometry analysis of Id1-Venus ESCs cultured in LIF + FCS with or without 48-h stimulation with 10 ng/mL BMP4 and/or 10 μM SB431542.

(I) Immunofluorescence for Id1 and Nanog in wild-type ESCs cultured in LIF + FCS with or without 48-h stimulation with 10 ng/mL BMP4 and 10 μM SB431542.

(J) Quantification of immunofluorescence signal for the cells in (I). Id1 is enriched in Nanog-low cells.

(legend continued on next page)

Galonska et al., 2015; Leeb et al., 2014; Yang et al., 2012). Much less is known about how pluripotency is protected during the transition between these states. We propose that cells regulate changes in signal responsiveness in order to protect pluripotency during this transition and that Id1 coordinates this process. In the absence of Id1, cells fail to transit robustly from pre-implantation to post-implantation stages of development.

Cells modulate signal responsiveness as they exit pluripotency (Kalkan et al., 2017; Zhou et al., 2013) and it has been proposed that prior to differentiation pluripotent cells enter a ‘‘transition state’’ or ‘‘formative’’ state in which they become more responsive to prevailing cues (Rué and Martinez Arias, 2015; Smith, 2017), an idea that is supported by our findings.

How Are Cells Protected from Differentiation after the Collapse of the Naive GRN?

In culture, ESCs do not commit to a primed state immediately after downregulating Nanog, but rather can reassemble a naive GRN and revert to naive pluripotency (Chambers et al., 2007; MacArthur et al., 2012). A proportion of Nanog-low cells are nevertheless spontaneously lost to differentiation (Chambers et al., 2007). Several lines of evidence suggest that the decision of Nanog-low cells to regain Nanog or to differentiate is stochastic (Abranches et al., 2014; Kalmar et al., 2009; MacArthur et al., 2012). This might prompt the assumption that no particular factor is brought into play to determine the ability of cells to retain pluripotency and return to a Nanog-high state.

However, the following observations from peri-implantation embryos call this assumption into question: in peri-implantation embryos, in contrast to the situation in culture, cells that lose Nanog after E4.5 neither return to a Nanog-high naive state (Xenopoulos et al., 2015) nor differentiate into extraembryonic endoderm (Grabarek et al., 2012). Rather, they are efficiently captured into a post-implantation epiblast state that is dependent on Nodal (Camus et al., 2006; Mesnard et al., 2006). The existence of cells in the embryo that lack both Nanog and Nodal activity points to the existence of another factor that protects these cells from differentiation. We propose that this factor is Id1.

Recent findings indicate that extraembryonic endoderm potency is not irreversibly lost but rather remains latent in epiblast cells during implantation (Nowotschin et al., 2019). This implies the existence of mechanisms that protect the epiblast from differentiating into extraembryonic endoderm throughout the course of pregastrulation development. Nanog performs this role in the E3.5 embryo (Mitsui et al., 2003), and we now propose that Id1 takes over this role immediately after downregulation of Nanog. In support of this idea, we find that Id1 can protect Nanog-null cells from differentiating into primitive endoderm.

BMP Maintains Epiblast Identity Specifically during the Transition between Naive and Primed States

Id1 is a target of BMP signaling (Hollnagel et al., 1999) that contributes to maintenance of pluripotency in ESCs cultured in LIF + BMP4 or LIF + FCS (Ying et al., 2003; Ying and Smith, 2003). It has been proposed that Id1 maintains Nanog expression (Galvin-Burgess et al., 2013; Romero-Lanman et al., 2012), but this seems inconsistent with the observation that Id1 is not co-expressed with Nanog *in vitro* or *in vivo*. We reconcile our findings with these reports by proposing that Id1 does not directly maintain Nanog expression, but rather increases the probability that Nanog-low cells will return to a Nanog-high state.

Our findings also explain the previously puzzling observation that BMP is required for maintaining pluripotency in ESCs cultured as a mixture of naive and primed states in LIF + FCS (Malaguti et al., 2013; Ying et al., 2003; Zhang et al., 2010) yet is not required for maintaining pluripotency in homogenous populations of naive cells (Graham et al., 2014; Morikawa et al., 2016; Zhao, 2003) nor primed cells (Brons et al., 2007; Tesar et al., 2007). Our model is also consistent with the observation that BMP is not required for pre-implantation development (Graham et al., 2014; Zhao, 2003) but is required to maintain pluripotency subsequently (Di-Gregorio et al., 2007).

Much is known about the transcriptional regulators required to escape naive pluripotency and establish a primed state (Betschinger et al., 2013; Buecker et al., 2014; Galonska et al., 2015; Leeb et al., 2014; Yang et al., 2012). For instance, the FGF target gene *Egr1* drives reorganization of enhancer binding as cells proceed to a primed state (Galonska et al., 2015; Kumar and Ivanova, 2015). Our data place Id1 upstream of these factors, operating to suppress *Egr1* and thus help to transiently stabilize the naive state in the absence of Nanog. We cannot, however, exclude the possibility that factors other than Egr1 also act downstream of Id1.

Id1 Confers Robustness to Early Development

Id1-null embryos progress through early development (Lyden et al., 1999), and Id1-null cells can differentiate *in vitro* (Romero-Lanman et al., 2012), so it is clear that Id1 is not absolutely required for early developmental transitions. Rather, we propose that Id1 makes early development more robust by shielding epiblast cells from pro-differentiation cues and ensuring that cells exit naive pluripotency only once signals to sustain the primed state are present. We confirm that Id1-null cells can proceed through early development in chimeric embryos but do so less robustly than their wild-type neighbors.

In summary, we propose that Id1 protects epiblast identity specifically during the transition from naive to primed states. As embryos progress through implantation, a build-up of Nodal simultaneously provides the environment that supports a primed epiblast state and suppresses expression of Id1 to permit the transition to this state.

(K) Flow cytometry analysis of Id1-Venus EpiSCs with or without 48-h stimulation with 10 ng/mL BMP4 and/or 10 μM SB431542.

(L) Immunofluorescence for Id1 and Nanog in wild-type EpiSCs with or without 48-h stimulation with 10 ng/mL BMP4 and 10 μM SB431542.

(M) Quantification of immunofluorescence signal for the cells in (L).

(N) Diagram illustrating the negative inputs of Nanog and Nodal on the expression of Id1. The results in Id1 being expressed only after Nanog is downregulated and before Nodal becomes active.

Scale bars, 30 μm. Statistical analyses were performed using a two-tailed unpaired Student’s t test. *$p < 0.05$, **$p < 0.01$, ***$p < 0.001$. See also Figure S6.

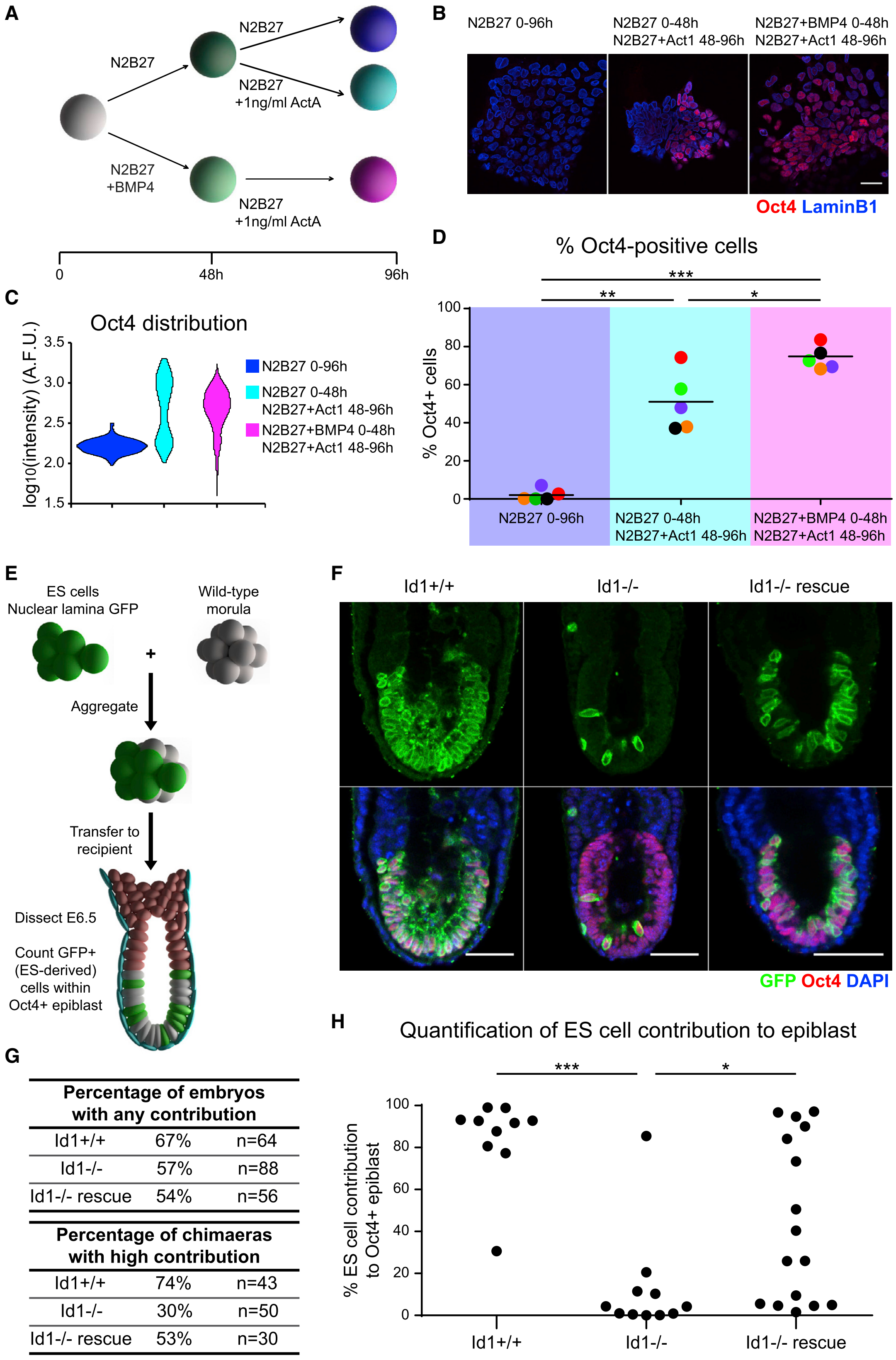

Percentage of embryos with any contribution		
Id1+/+	67%	n=64
Id1-/-	57%	n=88
Id1-/- rescue	54%	n=56

Percentage of chimaeras with high contribution		
Id1+/+	74%	n=43
Id1-/-	30%	n=50
Id1-/- rescue	53%	n=30

(legend on next page)

Our findings support the idea that in order for changes in cell fate to occur at the correct time and place, mechanisms must exist to ensure that differentiation is coordinated with changes in responsiveness to extrinsic cues. Such mechanisms ensure canalization during early development (Waddington, 1959) and also help to explain why it is not straightforward to control differentiation of pluripotent cells *in vitro* simply by controlling exposure to extrinsic signals.

STAR★METHODS

Detailed methods are provided in the online version of this paper and include the following:

- KEY RESOURCES TABLE
- LEAD CONTACT AND MATERIALS AVAILABILITY
- EXPERIMENTAL MODEL AND SUBJECT DETAILS
  - Animal Care and Use
  - Cell Lines
  - Cell Culture
- METHOD DETAILS
  - Plasmid Preparation
  - Transfection
  - Embryo Collection
  - Chimaera Generation
  - Embryo Immunofluorescence and Confocal Microscopy
  - Cell Immunofluorescence and Confocal Microscopy
  - Immunofluorescence Quantification
  - Gene Expression Analysis
  - Flow Cytometry
  - Clonal Self-renewal Assays and Alkaline Phosphatase Staining
  - Transcriptome (Microarray) Analyses
  - Western Blotting
  - Southern Blotting
- QUANTIFICATION AND STATISTICAL ANALYSIS
- DATA AND CODE AVAILABILITY

SUPPLEMENTAL INFORMATION

Supplemental Information can be found online at https://doi.org/10.1016/j.devcel.2019.05.032.

ACKNOWLEDGMENTS

We thank Robert Benezra for Id1-null cells and the Id1-Venus construct; Nicola Festuccia and Ian Chambers for the Nanog-tagRFP construct and Nanog-null cells; Chandrika Rao, Tulin Tatar, and Hongyu Shao for samples for pilot experiments; and Frederick Wong for help with embryo analysis. We thank Dónal O'Carroll, Val Wilson, Ian Chambers, Austin Smith, and members of the Lowell lab for helpful comments. S.L. is funded by Wellcome Trust Senior fellowship WT103789AIA, and G.B. was funded by Wellcome Trust Sir Henry Wellcome fellowship WT100133.

AUTHOR CONTRIBUTIONS

M.M. conceived and performed experiments and wrote the manuscript. R.P.M. performed experiments. G.B. performed experiments. C.-Y.L. provided reagents. S.L. conceived experiments, wrote the manuscript, and secured funding.

DECLARATION OF INTERESTS

The authors declare no competing interests.

Received: December 15, 2017
Revised: February 4, 2019
Accepted: May 13, 2019
Published: June 13, 2019

REFERENCES

Abranches, E., Guedes, A.M.V., Moravec, M., Maamar, H., Svoboda, P., Raj, A., and Henrique, D. (2014). Stochastic NANOG fluctuations allow mouse embryonic stem cells to explore pluripotency. Development *141*, 2770–2779.

Aloia, L., Gutierrez, A., Caballero, J.M., and Di Croce, L. (2015). Direct interaction between Id1 and Zrf1 controls neural differentiation of embryonic stem cells. EMBO Rep *16*, 63–70.

Arnold, S.J., and Robertson, E.J. (2009). Making a commitment: cell lineage allocation and axis patterning in the early mouse embryo. Nat. Rev. Mol. Cell Biol. *10*, 91–103.

Betschinger, J., Nichols, J., Dietmann, S., Corrin, P.D., Paddison, P.J., and Smith, A. (2013). Exit from pluripotency is gated by intracellular redistribution of the bHLH transcription factor Tfe3. Cell *153*, 335–347.

Blin, G., Sadurska, D., Migueles, R.P., Chen, N., Watson, J.A., and Lowell, S. (2018). Nessys: a novel method for accurate nuclear segmentation in 3D. bioRxiv, 502872.

Boroviak, T., Loos, R., Lombard, P., Okahara, J., Behr, R., Sasaki, E., Nichols, J., Smith, A., and Bertone, P. (2015). Lineage-specific profiling delineates the emergence and progression of naive pluripotency in mammalian embryogenesis. Dev. Cell *35*, 366–382.

Figure 7. Id1 Enables a Robust Transition from a Naive to a Primed Pluripotent State

(A) BMP4 enables a robust transition from naive to primed pluripotency *in vitro*. Experimental strategy: 2i + LIF cells were cultured in N2B27 in the presence or absence of 10 ng/mL BMP4 for 48 h. The cells were then exposed to low levels (1 ng/mL) of Activin A (ActA) for a further 48 h in the absence of BMP4 and assayed for their ability to retain Oct4 expression. Cells cultured in N2B27 throughout the experiment were used as a control for Oct4 downregulation.

(B) Representative images of the samples described in (A). Act1: 1 ng/mL Activin A. Scale bar, 30 μm.

(C) Distribution of Oct4 expression in the samples imaged in (B), calculated by immunofluorescence quantification. A.F.U.: Arbitrary fluorescence units.

(D) Percentage of Oct4-positive cells observed in the samples imaged in (B), over 5 independent experiments (color-coded dots). Horizontal bars represent the mean of the 5 experiments. Statistical analysis was performed using a one-way ANOVA followed by Tukey's multiple comparison test. $^{*}p < 0.05$, $^{**}p < 0.01$, $^{***}p < 0.001$.

(E) Id1 enables a robust transition from naive to primed pluripotency *in vivo*. Experimental strategy: labeled wild-type, Id1-null, and Id1-rescue ESCs were aggregated to wild-type morulae, then transferred to pseudopregnant females. The embryos were recovered at E6.5 and assessed for chimaerism.

(F) Representative images of $Id1^{+/+}$, $Id1^{-/-}$, and $Id1^{-/-}$ rescue chimeras, stained for GFP, Oct4, and DAPI. Scale bar, 30 μm.

(G) Quantification of ESC contribution to recovered embryos and percentage of chimeras with high ESC contribution to the epiblast. Analysis performed by microscopy prior to fixation.

(H) Quantification of ESC contribution to the Oct4-positive epiblast of recovered embryos. Analysis performed by nuclear segmentation and quantitative image analysis following immunofluorescence. Statistical analysis was performed using a Kruskal-Wallis test followed by Dunn's multiple comparison test. $^{*}p < 0.05$, $^{***}p < 0.001$.

See also Figure S7.

Brons, I.G., Smithers, L.E., Trotter, M.W., Rugg-Gunn, P., Sun, B., Chuva de Sousa Lopes, S.M., Howlett, S.K., Clarkson, A., Ahrlund-Richter, L., Pedersen, R.A., et al. (2007). Derivation of pluripotent epiblast stem cells from mammalian embryos. Nature *448*, 191–195.

Buecker, C., Srinivasan, R., Wu, Z., Calo, E., Acampora, D., Faial, T., Simeone, A., Tan, M., Swigut, T., and Wysocka, J. (2014). Reorganization of enhancer patterns in transition from naive to primed pluripotency. Cell Stem Cell *14*, 838–853.

Camus, A., Perea-Gomez, A., Moreau, A., and Collignon, J. (2006). Absence of Nodal signaling promotes precocious neural differentiation in the mouse embryo. Dev. Biol. *295*, 743–755.

Chambers, I., Silva, J., Colby, D., Nichols, J., Nijmeijer, B., Robertson, M., Vrana, J., Jones, K., Grotewold, L., and Smith, A. (2007). Nanog safeguards pluripotency and mediates germline development. Nature *450*, 1230–1234.

Chazaud, C., Yamanaka, Y., Pawson, T., and Rossant, J. (2006). Early lineage segregation between epiblast and primitive endoderm in mouse blastocysts through the Grb2-MAPK pathway. Dev. Cell *10*, 615–624.

Clavería, C., Giovinazzo, G., Sierra, R., and Torres, M. (2013). Myc-driven endogenous cell competition in the early mammalian embryo. Nature *500*, 39–44.

Coucouvanis, E., and Martin, G.R. (1999). BMP signaling plays a role in visceral endoderm differentiation and cavitation in the early mouse embryo. Development *126*, 535–546.

Davies, O.R., Lin, C.Y., Radzisheuskaya, A., Zhou, X., Taube, J., Blin, G., Waterhouse, A., Smith, A.J.H., and Lowell, S. (2013). Tcf15 primes pluripotent cells for differentiation. Cell Rep *3*, 472–484.

Díaz-Díaz, C., Fernandez de Manuel, L., Jimenez-Carretero, D., Montoya, M.C., Clavería, C., and Torres, M. (2017). Pluripotency surveillance by Myc-driven competitive elimination of differentiating cells. Dev. Cell *42*, 585–599.e4.

Di-Gregorio, A., Sancho, M., Stuckey, D.W., Crompton, L.A., Godwin, J., Mishina, Y., and Rodriguez, T.A. (2007). BMP signalling inhibits premature neural differentiation in the mouse embryo. Development *134*, 3359–3369.

Dunning, M.J., Smith, M.L., Ritchie, M.E., and Tavaré, S. (2007). Beadarray: R classes and methods for Illumina bead-based data. Bioinformatics *23*, 2183–2184.

Festuccia, N., Osorno, R., Halbritter, F., Karwacki-Neisius, V., Navarro, P., Colby, D., Wong, F., Yates, A., Tomlinson, S.R., and Chambers, I. (2012). Esrrb is a direct Nanog target gene that can substitute for Nanog function in pluripotent cells. Cell Stem Cell *11*, 477–490.

Galonska, C., Ziller, M.J., Karnik, R., and Meissner, A. (2015). Ground state conditions induce rapid reorganization of core pluripotency factor binding before global epigenetic reprogramming. Cell Stem Cell *17*, 462–470.

Galvin, K.E., Travis, E.D., Yee, D., Magnuson, T., and Vivian, J.L. (2010). Nodal signaling regulates the bone morphogenic protein pluripotency pathway in mouse embryonic stem cells. J. Biol. Chem. *285*, 19747–19756.

Galvin-Burgess, K.E., Travis, E.D., Pierson, K.E., and Vivian, J.L. (2013). TGF-β-superfamily signaling regulates embryonic stem cell heterogeneity: self-renewal as a dynamic and regulated equilibrium. Stem Cells *31*, 48–58.

Gentleman, R.C., Carey, V.J., Bates, D.M., Bolstad, B., Dettling, M., Dudoit, S., Ellis, B., Gautier, L., Ge, Y., Gentry, J., et al. (2004). Bioconductor: open software development for computational biology and bioinformatics. Genome Biol. *5*, R80.

Gomes Fernandes, M., Dries, R., Roost, M.S., Semrau, S., de Melo Bernardo, A., Davis, R.P., Ramakrishnan, R., Szuhai, K., Maas, E., Umans, L., et al. (2016). BMP-SMAD signaling regulates lineage priming, but is dispensable for self-renewal in mouse embryonic stem cells. Stem Cell Rep *6*, 85–94.

Grabarek, J.B., Zyzyńska, K., Saiz, N., Piliszek, A., Frankenberg, S., Nichols, J., Hadjantonakis, A.K., and Plusa, B. (2012). Differential plasticity of epiblast and primitive endoderm precursors within the ICM of the early mouse embryo. Development *139*, 129–139.

Graham, S.J.L., Wicher, K.B., Jedrusik, A., Guo, G., Herath, W., Robson, P., and Zernicka-Goetz, M. (2014). BMP signalling regulates the pre-implantation development of extra-embryonic cell lineages in the mouse embryo. Nat. Commun *5*, 5667.

Granier, C., Gurchenkov, V., Perea-Gomez, A., Camus, A., Ott, S., Papanayotou, C., Iranzo, J., Moreau, A., Reid, J., Koentges, G., et al. (2011). Nodal cis-regulatory elements reveal epiblast and primitive endoderm heterogeneity in the periimplantation mouse embryo. Dev. Biol. *349*, 350–362.

Guo, G., Yang, J., Nichols, J., Hall, J.S., Eyres, I., Mansfield, W., and Smith, A. (2009). Klf4 reverts developmentally programmed restriction of ground state pluripotency. Development *136*, 1063–1069.

Hamilton, W.B., and Brickman, J.M. (2014). Erk signaling suppresses embryonic stem cell self-renewal to specify endoderm. Cell Rep *9*, 2056–2070.

Hayashi, K., Ohta, H., Kurimoto, K., Aramaki, S., and Saitou, M. (2011). Reconstitution of the mouse germ cell specification pathway in culture by pluripotent stem cells. Cell *146*, 519–532.

Hollnagel, A., Oehlmann, V., Heymer, J., Rüther, U., and Nordheim, A. (1999). Id genes are direct targets of bone morphogenetic protein induction in embryonic stem cells. J. Biol. Chem. *274*, 19838–19845.

Hooper, M., Hardy, K., Handyside, A., Hunter, S., and Monk, M. (1987). HPRT-deficient (Lesch-Nyhan) mouse embryos derived from germline colonization by cultured cells. Nature *326*, 292–295.

Iacovino, M., Bosnakovski, D., Fey, H., Rux, D., Bajwa, G., Mahen, E., Mitanoska, A., Xu, Z., and Kyba, M. (2011). Inducible cassette exchange: a rapid and efficient system enabling conditional gene expression in embryonic and primary cells. Stem Cells *29*, 1580–1588.

Iacovino, M., Roth, M.E., and Kyba, M. (2014). Rapid genetic modification of mouse embryonic stem cells by inducible cassette exchange recombination. Methods Mol. Biol. *1101*, 339–351.

Kalkan, T., Olova, N., Roode, M., Mulas, C., Lee, H.J., Nett, I., Marks, H., Walker, R., Stunnenberg, H.G., Lilley, K.S., et al. (2017). Tracking the embryonic stem cell transition from ground state pluripotency. Development *144*, 1221–1234.

Kalmar, T., Lim, C., Hayward, P., Muñoz-Descalzo, S., Nichols, J., Garcia-Ojalvo, J., and Martinez Arias, A. (2009). Regulated fluctuations in nanog expression mediate cell fate decisions in embryonic stem cells. PLoS Biol. *7*, e1000149.

Kumar, I., and Ivanova, N. (2015). Moving toward the ground state. Cell Stem Cell *17*, 375–376.

Leeb, M., Dietmann, S., Paramor, M., Niwa, H., and Smith, A. (2014). Genetic exploration of the exit from self-renewal using haploid embryonic stem cells. Cell Stem Cell *14*, 385–393.

Lin, Y.C., Jhunjhunwala, S., Benner, C., Heinz, S., Welinder, E., Mansson, R., Sigvardsson, M., Hagman, J., Espinoza, C.A., Dutkowski, J., et al. (2010). A global network of transcription factors, involving E2A, EBF1 and FoxO1, that orchestrates B cell fate. Nat. Immunol *11*, 635–643.

Lyden, D., Young, A.Z., Zagzag, D., Yan, W., Gerald, W., O'Reilly, R., Bader, B.L., Hynes, R.O., Zhuang, Y., Manova, K., et al. (1999). Id1 and Id3 are required for neurogenesis, angiogenesis and vascularization of tumour xenografts. Nature *401*, 670–677.

MacArthur, B.D., Sevilla, A., Lenz, M., Müller, F.J., Schuldt, B.M., Schuppert, A.A., Ridden, S.J., Stumpf, P.S., Fidalgo, M., Ma'ayan, A., et al. (2012). Nanog-dependent feedback loops regulate murine embryonic stem cell heterogeneity. Nat. Cell Biol. *14*, 1139–1147.

Malaguti, M., Nistor, P.A., Blin, G., Pegg, A., Zhou, X., and Lowell, S. (2013). Bone morphogenic protein signalling suppresses differentiation of pluripotent cells by maintaining expression of E-cadherin. ELife *2*, e01197.

Massari, M.E., and Murre, C. (2000). Helix-loop-helix proteins: regulators of transcription in eucaryotic organisms. Mol. Cell. Biol. *20*, 429–440.

Mesnard, D., Guzman-Ayala, M., and Constam, D.B. (2006). Nodal specifies embryonic visceral endoderm and sustains pluripotent cells in the epiblast before overt axial patterning. Development *133*, 2497–2505.

Mitsui, K., Tokuzawa, Y., Itoh, H., Segawa, K., Murakami, M., Takahashi, K., Maruyama, M., Maeda, M., and Yamanaka, S. (2003). The homeoprotein Nanog is required for maintenance of pluripotency in mouse epiblast and ES cells. Cell *113*, 631–642.

Morikawa, M., Koinuma, D., Mizutani, A., Kawasaki, N., Holmborn, K., Sundqvist, A., Tsutsumi, S., Watabe, T., Aburatani, H., Heldin, C.H., et al.

(2016). BMP sustains embryonic stem cell self-renewal through distinct functions of different Krüppel-like factors. Stem Cell Rep *6*, 64–73.

Nam, H.S., and Benezra, R. (2009). High levels of Id1 expression define B1 type adult neural stem cells. Cell Stem Cell *5*, 515–526.

Nichols, J., Silva, J., Roode, M., and Smith, A. (2009). Suppression of Erk signalling promotes ground state pluripotency in the mouse embryo. Development *136*, 3215–3222.

Nichols, J., and Smith, A. (2009). Naive and primed pluripotent states. Cell Stem Cell *4*, 487–492.

Norton, J.D. (2000). ID helix-loop-helix proteins in cell growth, differentiation and tumorigenesis. J. Cell. Sci. *113*, 3897–3905.

Nowotschin, S., Setty, M., Kuo, Y.-Y., Liu, V., Garg, V., Sharma, R., Simon, C.S., Saiz, N., Gardner, R., Boutet, S.C., et al. (2019). The emergent landscape of the mouse gut endoderm at single-cell resolution. Nature *569*, 361–367.

Osorno, R., Tsakiridis, A., Wong, F., Cambray, N., Economou, C., Wilkie, R., Blin, G., Scotting, P.J., Chambers, I., and Wilson, V. (2012). The developmental dismantling of pluripotency is reversed by ectopic Oct4 expression. Development *139*, 2288–2298.

Pollard, S.M., Benchoua, A., and Lowell, S. (2006). Neural stem cells, neurons, and glia. Methods Enzymol *418*, 151–169.

Roberts, E.C., Deed, R.W., Inoue, T., Norton, J.D., and Sharrocks, A.D. (2001). Id helix-loop-helix proteins antagonize pax transcription factor activity by inhibiting DNA binding. Mol. Cell. Biol. *21*, 524–533.

Romero-Lanman, E.E., Pavlovic, S., Amlani, B., Chin, Y., and Benezra, R. (2012). Id1 maintains embryonic stem cell self-renewal by up-regulation of Nanog and repression of brachyury expression. Stem Cells Dev. *21*, 384–393.

Rué, P., and Martinez Arias, A. (2015). Cell dynamics and gene expression control in tissue homeostasis and development. Mol. Syst. Biol. *11*, 792.

Sancho, M., Di-Gregorio, A., George, N., Pozzi, S., Sánchez, J.M., Pernaute, B., and Rodríguez, T.A. (2013). Competitive interactions eliminate unfit embryonic stem cells at the onset of differentiation. Dev. Cell *26*, 19–30.

Smith, A. (2017). Formative pluripotency: the executive phase in a developmental continuum. Development *144*, 365–373.

Southern, E.M. (1975). Detection of specific sequences among DNA fragments separated by gel electrophoresis. J. Mol. Biol. *98*, 503–517.

Suzuki, A., Raya, Á., Kawakami, Y., Morita, M., Matsui, T., Nakashima, K., Gage, F.H., Rodríguez-Esteban, C., and Izpisúa Belmonte, J.C. (2006). Nanog binds to Smad1 and blocks bone morphogenetic protein-induced differentiation of embryonic stem cells. Proc. Natl. Acad. Sci. USA *103*, 10294–10299.

Tesar, P.J., Chenoweth, J.G., Brook, F.A., Davies, T.J., Evans, E.P., Mack, D.L., Gardner, R.L., and McKay, R.D. (2007). New cell lines from mouse epiblast share defining features with human embryonic stem cells. Nature *448*, 196–199.

Waddington, C.H. (1959). Canalization of development and genetic assimilation of acquired characters. Nature *183*, 1654–1655.

Wettenhall, J.M., and Smyth, G.K. (2004). limmaGUI: a graphical user interface for linear modeling of microarray data. Bioinformatics *20*, 3705–3706.

Xenopoulos, P., Kang, M., Puliafito, A., Di Talia, S., and Hadjantonakis, A.K. (2015). Heterogeneities in Nanog expression drive stable commitment to pluripotency in the mouse blastocyst. Cell Rep *10*, 1508–1520.

Yamanaka, Y., Lanner, F., and Rossant, J. (2010). FGF signal-dependent segregation of primitive endoderm and epiblast in the mouse blastocyst. Development *137*, 715–724.

Yang, S.H., Kalkan, T., Morrisroe, C., Smith, A., and Sharrocks, A.D. (2012). A genome-wide RNAi screen reveals MAP kinase phosphatases as key ERK pathway regulators during embryonic stem cell differentiation. PLoS Genet. *8*, e1003112.

Yates, P.R., Atherton, G.T., Deed, R.W., Norton, J.D., and Sharrocks, A.D. (1999). Id helix-loop-helix proteins inhibit nucleoprotein complex formation by the TCF ETS-domain transcription factors. EMBO J *18*, 968–976.

Ying, Q.L., Nichols, J., Chambers, I., and Smith, A. (2003). BMP induction of Id proteins suppresses differentiation and sustains embryonic stem cell self-renewal in collaboration with STAT3. Cell *115*, 281–292.

Ying, Q.-L., Wray, J., Nichols, J., Batlle-Morera, L., Doble, B., Woodgett, J., Cohen, P., and Smith, A. (2008). The ground state of embryonic stem cell self-renewal. Nature *453*, 519–523.

Ying, Q.L., and Smith, A.G. (2003). Defined conditions for neural commitment and differentiation. Methods Enzymol *365*, 327–341.

Zhang, K., Li, L., Huang, C., Shen, C., Tan, F., Xia, C., Liu, P., Rossant, J., and Jing, N. (2010). Distinct functions of BMP4 during different stages of mouse ES cell neural commitment. Development *137*, 2095–2105.

Zhao, G.Q. (2003). Consequences of knocking out BMP signaling in the mouse. Genesis *35*, 43–56.

Zhou, X., Smith, A.J.H., Waterhouse, A., Blin, G., Malaguti, M., Lin, C.Y., Osorno, R., Chambers, I., and Lowell, S. (2013). Hes1 desynchronizes differentiation of pluripotent cells by modulating STAT3 activity. Stem Cells *31*, 1511–1522.

STAR★METHODS

KEY RESOURCES TABLE

REAGENT or RESOURCE	SOURCE	IDENTIFIER
Antibodies		
Rat Monoclonal Anti-Cdh1	Sigma-Aldrich	Cat#U3254; RRID: AB_477600
Rabbit Polyclonal Anti-Cleaved Caspase-3 (Asp175)	Cell Signaling Technology	Cat#9661; RRID: AB_2341188
Rabbit Monoclonal Anti-Egr1	Thermo Fisher	Cat#MA5-15009; RRID: AB_10982091
Goat Polyclonal Anti-Gata6	R&D	Cat#AF1700; RRID: AB_2108901
Chicken Polyclonal Anti-GFP	Abcam	Cat#ab3970; RRID: AB_300798
Goat Polyclonal Anti-Klf4	R&D	Cat#AF3158; RRID: AB_2130245
Rabbit Monoclonal Anti-Id1	Biocheck	Cat#BCH-1/37-2; RRID: AB_2713996
Rabbit Polyclonal Anti-LaminB1	Abcam	Cat#ab16048; RRID: AB_443298
Rat Monoclonal Anti-Nanog	Thermo Fisher	Cat#14-5761-80; RRID: AB_763613
Mouse Monoclonal Anti-Oct4	Santa Cruz	Cat#sc-5279; RRID: AB_628051
Goat Polyclonal Anti-Oct6	Santa Cruz	Cat#sc-11661; RRID: AB_2268536
Rabbit Monoclonal Anti-Phospho-p44/42 MAPK (Erk1/2) (Thr202/Tyr204)	Cell Signaling Technology	Cat#4370; RRID: AB_2315112
Rabbit Polyclonal Anti-tRFP	Evrogen	Cat#AB233; RRID: AB_2571743
Chemicals, Peptides, and Recombinant Proteins		
Benzyl alcohol	Alfa Aesar	Cat#100-51-6
Benzyl benzoate	Sigma	Cat#B9550
Tyrode's solution, acidic	Sigma	Cat#1788
DAPI	Biotium	Cat#40043
Prolong Gold Antifade Mountant	Thermo Fisher	Cat#P36930
Doxycyline Hyclate	Sigma-Aldrich	Cat#D9891
Recombinant Human/Murine/Rat Activin A (E. coli derived)	Peprotech	Cat#120-14E
Recombinant Human BMP4	R&D	Cat#314-BP
Recombinant Human FGF basic	R&D	Cat#233-FB
CHIR 99021	Axon Medchem	Cat#1386
LDN 193189	Axon Medchem	Cat#1509
PD 0325901	Axon Medchem	Cat#1408
SB431542	Abcam	Cat#ab146590
B-27 Supplement	Gibco	Cat#17504044
N-2 Supplement	Gibco	Cat#17502048
KnockOut Serum Replacement (KSR)	Gibco	Cat#10828028
Fibronectin from bovine plasma solution	Sigma	Cat#F1141
Laminin	Sigma	Cat#L2020
Poly-L-ornithine solution 0.1%	Sigma	Cat#P4957
Lipofectamine 3000 Transfection Reagent	Life Technologies	Cat#L3000008
PerfectHyb Plus Hybridization Buffer	Sigma	Cat#H7033
Critical Commercial Assays		
Illumina TotalPrep RNA Amplification Kit	Ambion	Cat#AMIL1791
Deposited Data		
Raw and normalysed microarray data	This study	GEO: GSE108226
Experimental Models: Cell Lines		
Mus musculus: E14Ju09 mouse ESCs (129/Ola, male)	Hamilton and Brickman, 2014	N/A
Mus musculus: Id1V mouse ESCs (129/Ola, male)	Malaguti et al., 2013	N/A

(*Continued on next page*)

Continued		
REAGENT or RESOURCE	SOURCE	IDENTIFIER
Mus musculus: IVNR mouse ESCs (129/Ola, male)	This study	N/A
Mus musculus: 111B (*Id1*+/+) and 139 D (*Id1*-/-) mouse ESCs (129/Sv, sex unknown)	Romero-Lanman et al., 2012	N/A
Mus musculus: Id1-rescue mouse ESCs (129/Sv, sex unknown)	This study	N/A
Mus musculus: NLS-GFP-EmdTM labelled 111B, 139 D, Id1-rescue mouse ESCs (129/Sv, sex unknown)	This study	N/A
Mus musculus: TβC44cre6 (*Nanog*-/-) ESCs (129/Ola, male)	Chambers et al., 2007	N/A
Mus musculus: Nanog-rescue mouse ESCs (129/Ola, male)	This study	N/A
Mus musculus: *Tcf15*-/- ESCs (129/Ola, male)	This study	N/A
Mus musculus: 3xFlag-Id1 inducible mouse ESCs (129/Ola, male)	Malaguti et al. 2013	N/A
Mus musculus: A2lox.Cre ESCs (129/Ola, male)	Iacovino et al., 2011	N/A
Mus musculus: 3xFlag-E47-E47 inducible mouse ESCs (129/Ola, male)	This study	N/A
Experimental Models: Organisms/Strains		
Mus musculus: MF1: outbred	OLAC	N/A
Mus musculus: CD-1: outbred	Charles River	CD-1
Mus musculus: F1: B6CBAF1	Charles River	B6CBAF1
Oligonucleotides		
See Table S2 for list of primers used in this study	N/A	N/A
Recombinant DNA		
pPyCAG-NLS-GFP-EmdTM-Ires-Pac	This study	N/A
pPyCAG-tagRFP-IP backbone for subcloning of gene of interest	This study	N/A
p2loxCre	Iacovino et al., 2011	Addgene Plasmid #34635
Software and Algorithms		
R	R Core Team	http://www.R-project.org
Bioconductor	Gentleman et al., 2004	http://bioconductor.org
Beadarray R package	Dunning et al., 2007	http://bioconductor.org/packages/release/bioc/html/beadarray.html
Limma R package	Wettenhall and Smyth, 2004	https://bioconductor.org/packages/release/bioc/html/limma.html
NesSys	Blin et al., 2018	https://framagit.org/pickcellslab/nessys

LEAD CONTACT AND MATERIALS AVAILABILITY

Further information and requests for resources and reagents should be directed to and will be fulfilled by the Lead Contact, Sally Lowell (sally.lowell@ed.ac.uk).

EXPERIMENTAL MODEL AND SUBJECT DETAILS

Animal Care and Use

Animal experiments were performed under the UK Home Office project license PEEC9E359, approved by the Animal Welfare and Ethical Review Panel of the University of Edinburgh and within the conditions of the Animals (Scientific Procedures) Act 1986.

Cell Lines

E14Ju09 ESCs are a male wild-type clonal cell line derived in-house from E14tg2a ESCs, with a 129/Ola genetic background (Hamilton and Brickman, 2014; Hooper et al., 1987). Id1V ESCs (male) were generated by targeting E14Ju09 ESCs with an Id1-Venus targeting construct (Malaguti et al., 2013; Nam and Benezra, 2009). IVNR ESCs (male) were generated by targeting Id1V ESCs with a Nanog-tagRFP targeting construct, which was obtained from Dr. Nicola Festuccia in Dr. Ian Chambers' laboratory. Id1-null ESCs and

control wild-type cells (129sv genetic background, sex unknown) were obtained from Dr. Robert Benezra (Romero-Lanman et al., 2012). Nuclear envelope GFP-labelled Id1-null and control wild-type clonal ESC lines were obtained by random integration of a *pPyCAG-NLS-GFP-EmdTM-IRES-Pac* construct. "Id1-rescue" clonal ESC lines were generated by random integration of a *pPyCAG-3xFlag-Id1-IRES-Pac* into unlabelled Id1-null ESCs, and of a *pPyCAG-3xFlag-Id1-IRES-HygroR* into labelled Id1-null ESCs. Nanog-null ESCs (TβC44cre6, male) were derived from E14tg2a ESCs and were obtained from Dr. Ian Chambers (Chambers et al., 2007). "Nanog-rescue" clonal ESC lines were generated by random integration of *pPyCAG-3xFlag-Nanog-IRES-Pac*, *pPyCAG-3xFlag-Id1-IRES-Pac* or *pPyCAG-3xFlag-GFP-IRES-Pac* constructs into Nanog-null ESCs. Tcf15-null ESCs (male) were derived from E14Ju09 ESCs by replacing *Tcf15* Exon 1 with a *Venus-polyA* transgene (CYL, SL in preparation). Inducible 3xFlag-Id1 ESCs (male) were generated by random integration of a *CAG-rtTA-IRES-Bls* construct and of a *tetO-3xFlag-Id1-Pgk-HygroR* construct into E14Ju09 ESCs (Malaguti et al., 2013). Inducible 3xFlag-E47-E47 ESCs (male) were generated making use of the A2lox inducible cassette exchange cell line (Iacovino et al., 2014).

Cell Culture

Mouse embryonic stem cells were routinely maintained on gelatinised culture vessels in Glasgow Minimum Essential Medium (GMEM, Sigma) supplemented with 10% foetal calf serum (FCS, APS), 100U/ml LIF (produced in-house), 100nM 2-mercaptoethanol (Gibco), 1X non-essential amino acids (Gibco), 2mM L-Glutamine (Gibco), 1mM Sodium Pyruvate (Gibco) ("LIF+FCS culture"). 2i+LIF culture was performed as previously described (Ying et al., 2008): cells were cultured in N2B27 medium supplemented with 1μM PD0325901 (Axon Medchem), 3μM CHIR99021 (Axon Medchem) and 100U/ml LIF (produced in-house) on culture vessels coated sequentially with poly-L-ornithine (Sigma) and 5μg/ml laminin (Sigma). N2B27 medium was prepared as previously described (Pollard et al., 2006). Its composition is a 1:1 mixture of DMEM/F12 (Gibco) and Neurobasal Medium (Gibco), supplemented with 0.5X N2 Supplement (Gibco), 0.5X B27 Supplement (Gibco), 2mM L-Glutamine (Gibco) and 100nM 2-mercaptoethanol (Gibco). Epiblast stem cells were derived from embryonic stem cells *in vitro* as previously described (Guo et al., 2009), by transferring ESCs to EpiSC culture medium on cell culture vessels coated with 7.5μg/ml fibronectin (Sigma), and passaging them every 1-2 days. EpiSCs were used for experimentation between passages 10 and 20. EpiSC culture medium composition is as previously described (Osorno et al., 2012): N2B27 medium supplemented with 10ng/ml Fgf2 (R&D) and 20ng/ml Activin A (R&D). EpiLC differentiation was performed as previously described (Hayashi et al., 2011). Briefly, 2i+LIF cells were plated on cell culture vessels coated with 7.5μg/ml fibronectin (Sigma) in EpiLC medium at a density of $2.5x10^4$ cells/cm^2. EpiLC medium consists of N2B27 medium supplemented with 10ng/ml Fgf2 (R&D), 20ng/ml Activin A (R&D) and 1% KSR (Gibco). Medium was changed 24h after plating.Cells were cultured at 37°C in 5% CO_2.

METHOD DETAILS

Plasmid Preparation

pPyCAG overexpression plasmids and p2lox cassette exchange plasmids were generated through conventional restriction enzyme-mediated ligation of DNA fragments flanked by convenient restriction sites. The DNA sequences of genes of interest were amplified from mouse ESC cDNA. The nuclear envelope GFP overexpression construct encodes a fusion protein comprising an N-terminal NLS, followed by GFP, and a C-terminal sequence consisting of the transmembrane domain of the inner nuclear membrane protein emerin (structure: *pPyCAG-NLS-GFP-EmdTM-IRES-Pac*).

Transfection

Overexpression plasmids were lipofected into cells using Lipofectamine 3000 reagent (Invitrogen), following the manufacturer's instructions. p2lox cassette exchange were nucleofected into A2loxCre parental cells as previously described (Iacovino et al., 2014).

Embryo Collection

Pre- and peri-implantation embryos were obtained by flushing uteri with a large-bore blunted needle in M2 medium (Sigma). Post-implantation embryos were dissected at 5.5 and 6.5 d.p.c. in M2 medium. The sex of embryos used in this study was not determined.

Chimaera Generation

F1 female mice were superovulated (100 IU/ml PMSG, ProSpec, and 100 IU/ml HCG, Intervet, intraperitoneal injections 48h apart) and crossed with wild-type stud male mice. Pregnant mice were culled at 2.5 d.p.c. by cervical dislocation, ovaries with oviducts were dissected and collected in pre-warmed M2 medium. Oviducts were flushed using PBS and a 20-gauge needle attached to a 1ml syringe and filled with PB1. 2.5 d.p.c. embryos were collected and washed in PB1, the zona pellucida was removed using acidic Tyrode's solution (Sigma), and transferred to a plate with incisions where one clump of 8-15 cells were added to each embryo. Embryos were then incubated at 37°C in 5% CO_2 for 24h prior to transfer to pseudopregnant recipients, or for up to 72h for assessment of pre-implantation chimaerism. Blastocysts were selected and collected to be transferred into the uterus of a pseudopregnant CD-1 female. Embryos were dissected at 6.5 d.p.c. in M2 medium and observed for chimeric ESC contribution under an Olympus IX51 microscope, prior to fixation and immunostaining.

Embryo Immunofluorescence and Confocal Microscopy

Embryos were fixed with 4% formaldehyde/PBS/0.1% Triton X-100 (Sigma) for 10 (pre-implantation), 20 (peri-implantation) or 30 (post-implantation) minutes and quenched with 50mM ammonium chloride. Cellular permeabilization was carried out for 10 min in PBS/0.1% Triton X-100. The embryos were incubated in primary antibody in 3% donkey serum/PBS/0.1% Triton X-100 overnight, and subjected to 3 washes in PBS/0.1% Triton X-100. Secondary antibodies were applied subsequently for 2h to overnight, followed by 3 washes in PBS/0.1% Triton X-100. Embryos were then stained with DAPI (Biotium), mounted in PBS droplets covered with mineral oil in "microscope rings", and imaged on a Leica SP8 confocal microscope. Alternatively, following staining, chimaeric embryos requiring immunostaining quantification were dehydrated in methanol series in PBS/0.1% Triton X-100, clarified in 50% methanol/ 50% BABB (benzyl alcohol:benzyl benzoate 1:2 ratio, Alfa Aesar and Sigma), transferred into 100% BABB in glass capillaries and imaged on a Leica SP8 confocal microscope.

Cell Immunofluorescence and Confocal Microscopy

Cells for immunofluorescence were cultured on flamed glass coverslips coated with 7.5μg/ml fibronectin (for adherent culture), or cytospun onto polysine adhesion slides (Thermo Fisher) using a Shandon Cytospin 3 centrifuge (for sorted samples in suspension). They were fixed in 4% formaldehyde/PBS, quenched with 50mM ammonium chloride, blocked in 3% donkey serum and 0.1% Triton X-100. Cells were then incubated with primary antibody for 3 h at room temperature, washed 3 times with PBS, incubated with secondary antibody and/or 100ng/ml DAPI for 1h at room temperature, washed 3 times with PBS, mounted in ProLong Gold Antifade Mountant (Molecular Probes), and imaged on a Leica SP8 confocal microscope. Where recommended by antibody manufacturers, a methanol permeabilisation step was included prior to blocking.

Immunofluorescence Quantification

Nuclear immunofluorescence signal was quantified using nuclear segmentation based on nuclear envelope staining or DAPI, as well as manual editing of segmentation results, making use of the NesSys software described in (Blin et al., 2018).

Gene Expression Analysis

Total RNA was extracted from cells making use of the Absolutely RNA Miniprep Kit (Stratagene). 300ng total RNA were reverse transcribed into cDNA making use of M-MLV Reverse Transcriptase (Invitrogen). qRT-PCR was performed using the Universal ProbeLibrary system (Roche) with a Lightcycler 480 II instrument. Expression data are presented relative to the geometric mean of the housekeeping genes *Sdha*, *Tbp* and *Ywhaz*. The sequences of the primers used in this study are listed in Table S2.

Flow Cytometry

Cells were dissociated into single cell suspensions in ice-cold PBS+10% FCS, in the presence of either 100ng/ml DAPI or 1μg/ml propidium iodide to stain dead cells. Analysis of fluorescence was performed on a BD FACSCalibur. Cell sorting was performed on a BD FACSAria.

Clonal Self-renewal Assays and Alkaline Phosphatase Staining

Cells were plated 10-30 cells/cm^2 in media as indicated in figure legends, and media were changed every other day. After 7 days, alkaline phosphatase staining was performed using the Leukocyte Alkaline Phosphatase Kit (Sigma).

Transcriptome (Microarray) Analyses

Sample preparation for microarrays was performed as previously described (Davies et al., 2013). 100 ng of total RNA were reverse transcribed into double-stranded cDNA and transcribed/amplified into biotin labelled cRNA using an Illumina TotalPrep RNA Amplification Kit (Ambion). Labelled RNA was submitted to the WTCRF MRC Human Genetics Unit (University of Edinburgh) for further processing. cRNA quality was checked using an Agilent 2100 Bioanalyser and hybridisation performed on an MouseWG-6 v2 BeadChip (Illumina). Raw data were processed in R using the beadarray (Dunning et al., 2007) and limma (Wettenhall and Smyth, 2004) packages from the Bioconductor suite (Gentleman et al., 2004). Briefly, expression data were quantile-normalised and log_2-transformed before assessing differential expression with the limma algorithms. Principal component analysis was performed using the prcomp() function in the core R stats package. Quantile-normalised microarray data are available in Table S1.

Western Blotting

Cells were lysed in RIPA buffer + 1X PMSF (Alpha Diagnostics). 20μg protein lysates were run on 4%-12% NuPage Bis-Tris Gel (Novex) and transferred onto Amersham Hybond ECL Nitrocellulose Membrane (GE Healthcare). Membranes were blocked in 5% Amersham ECL Prime Blocking Agent (GE Healthcare) + 0.1% Tween 20 (Sigma) in PBS. Membranes were incubated in primary antibody overnight at 4°C, washed 3 times in PBS + 0.1% Tween 20, incubated in HRP-conjugated secondary antibody for 1 h at room temperature and washed 3 times in PBS + 0.1% Tween 20. The membrane was incubated in Amersham ECL Western Blotting Detection Reagent (GE Healthcare) or Amersham ECL Prime Western Blotting Detection Reagent (GE Healthcare), depending on the expected strength of signal. The membranes were used to expose Amersham Hyperfilm ECL (GE Healthcare), and films were developed using a Konica SRX-101A Medical Film Processor.

Southern Blotting

Genomic DNA was extracted from mouse ESCs using the DNeasy Blood and Tissue kit (Qiagen). Southern blotting was performed as previously described (Southern, 1975). Briefly, 5μg genomic DNA was digested with 100U BamHI-HF (NEB) overnight at 37°C, in the presence of 2.5mM spermidine (Sigma). The DNA was ethanol-precipitated, resuspended in 20μl dH_2O, and run on a 0.8% w/v agarose/TAE gel. λ DNA-HindIII digest was loaded as a size marker. The gel was placed in denaturing solution (aqueous solution of 86.77g/l sodium chloride + 20g/l sodium hydroxide) for 40 minutes at room temperature, and neutralising solution (aqueous solution of 116.8g/l sodium chloride, 121.1g/l Tris base, pH8.0) for 40 minutes at room temperature. DNA was transferred onto a positively charged nylon membrane (Roche) by capillary transfer of 20X SSC buffer (aqueous solution of 175.2g/l sodium chloride + 88.2g/l Tris base, pH7.4) for 48 h at room temperature. The membrane was then baked for 1 h at 120°C, rinsed in 2X SSC, and placed in a glass hybridisation bottle with PerfectHyb Plus (Sigma) hybridisation buffer at 65°C for 1 h. Probes were generated by PCR amplification of sequences of interest (Table S2), and labelled with [α-^{32}P]dCTP using the Amersham Rediprime II DNA Labelling System (GE Healthcare), alongside the control λ HindIII DNA to detect the size marker. 500μl sonicated herring sperm DNA (Sigma) and the probes were added to the hybridisation bottle overnight at 65°C. The hybridisation solution was removed, and the membrane was washed twice for 15 minutes in 2X SSC+0.1% SDS, and once for 30 minutes in 0.5X SSC+0.1% SDS. The membrane was used to expose Amersham Hyperfilm ECL (GE Healthcare), and films were developed using a Konica SRX-101A Medical Film Processor.

QUANTIFICATION AND STATISTICAL ANALYSIS

Definition of statistical significance and size of n is indicated in Figure Legends. Statistical analysis methods include two-tailed Student's t-test for comparison of two samples, one-way ANOVA followed by Tukey's multiple comparison test for comparison of more than two samples with normal distributions, Kruskal-Wallis test followed by Dunn's multiple comparison test for comparison of more than two samples with non-normal distributions, and empirical Bayes moderated t-statistics for linear model fit contrasts for microarray data, with p-values adjusted for multiple testing correction using the Benjamini & Hochberg method.

DATA AND CODE AVAILABILITY

The accession number for the raw and normalized microarray data reported in this paper is GEO: GSE108226.

Developmental Cell, Volume *50*

Supplemental Information

Id1 Stabilizes Epiblast Identity by Sensing Delays in Nodal Activation and Adjusting the Timing of Differentiation

Mattias Malaguti, Rosa Portero Migueles, Guillaume Blin, Chia-Yi Lin, and Sally Lowell

FIGURE S1

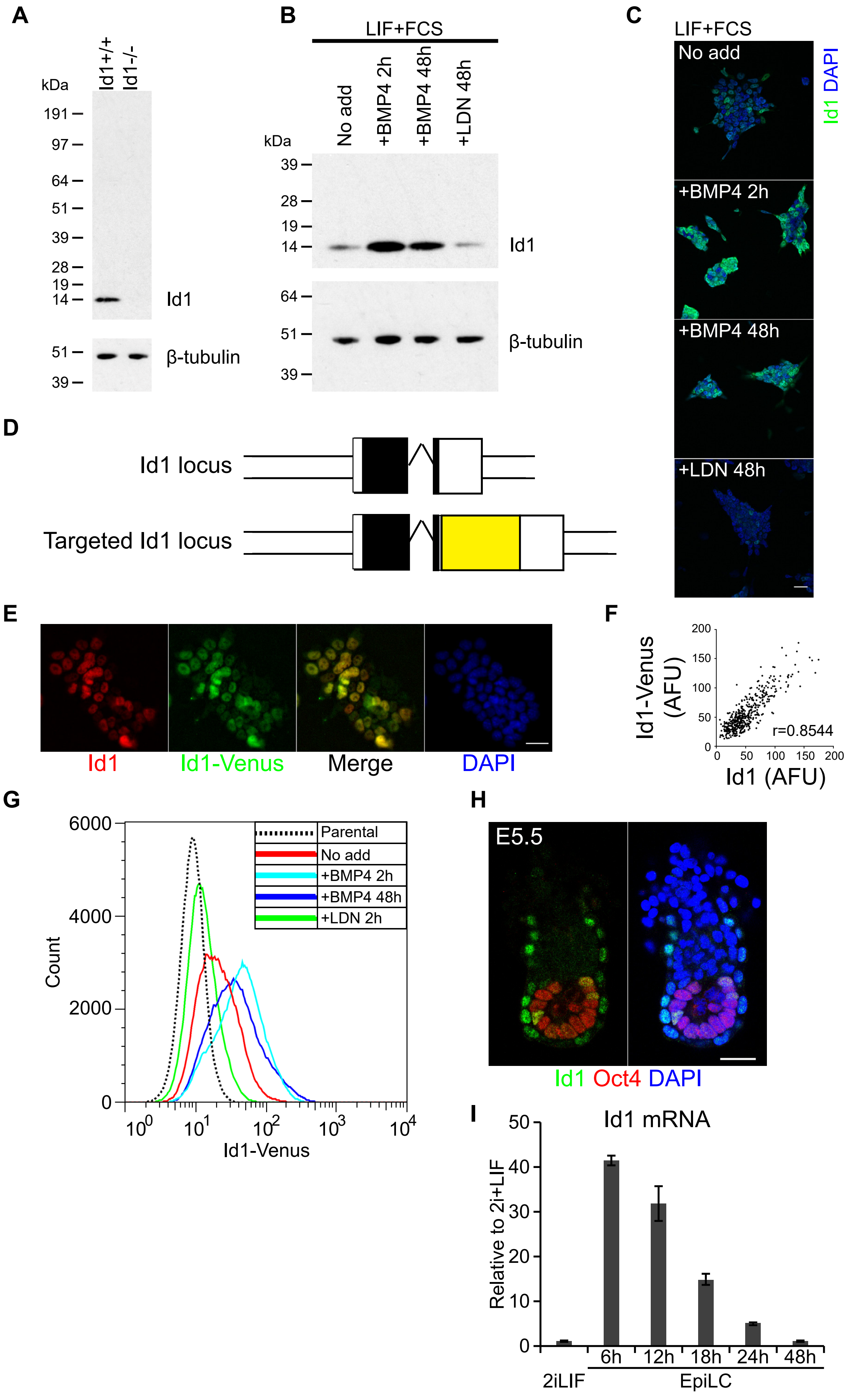
A
Id1+/+
Id1-/-
kDa
191
97
64
51
39
28
19
14
Id1
51
β-tubulin
39
B
LIF+FCS
No add
+BMP4 2h
+BMP4 48h
+LDN 48h
kDa
39
28
19
14
Id1
64
51
β-tubulin
39
C
LIF+FCS
No add
Id1 DAPI
+BMP4 2h
+BMP4 48h
+LDN 48h
D
Id1 locus
Targeted Id1 locus
E
Id1
Id1-Venus
Merge
DAPI
F
Id1-Venus (AFU)
Id1 (AFU)
r=0.8544
G
Parental
No add
+BMP4 2h
+BMP4 48h
+LDN 2h
Count
Id1-Venus
H
E5.5
Id1 Oct4 DAPI
I
Id1 mRNA
Relative to 2i+LIF
6h
12h
18h
24h
48h
2iLIF
EpiLC

Figure S1. Id1 immunodetection and the Id1-Venus reporter both provide faithful readouts of BMP responsiveness, Related to Figure 1.

(A) Western blot for Id1 in wild-type and Id1-null ES cells illustrates the high specificity of the Id1 antibody. B-tubulin is included as a loading control.

(B) Western blot for Id1 in wild-type ES cells cultured in LIF+FCS +/- 10ng/ml BMP4 for 2h or 48h, or with the addition of 100nM of the BMP inhibitor LDN193189 (LDN) for 48h. As expected, Id1 is upregulated upon BMP4 stimulation and downregulated upon BMP inhibition with LDN. B-tubulin is included as a loading control.

(C) Immunofluorescence staining of wild-type ES cells cultured in LIF+FCS with no addition, with the addition of 10ng/ml BMP4 for 2h or 48h, or with the addition of 100nM of the BMP inhibitor LDN193189 (LDN) for 48h. As expected, Id1 is upregulated upon BMP4 stimulation and downregulated upon BMP inhibition with LDN. Scale bar: 30µm.

(D) Diagrammatic structure of the Id1 loci in Id1-Venus reporter ES cells. Venus is fused to the C-terminus of Id1 via a flexible linker.

(E) Co-staining for Id1 and Venus in Id1-Venus cells cultured in LIF+FCS. Scale bar: 30µm.

(F) Immunofluorescence quantification of the cells in (E) show high correlation between Id1 and Venus expression (Pearson's r=0.8544). A.F.U.: arbitrary fluorescence units.

(G) Flow cytometry analysis of Id1-Venus cells cultured in LIF+FCS with no addition, with the addition of 10ng/ml BMP4 for 2h or 48h, or with the addition of 100nM of the BMP inhibitor LDN193189 (LDN). Parental non-fluorescent cells are included as a control. As expected, Id1-Venus is upregulated upon BMP4 stimulation and downregulated upon BMP inhibition with LDN.

(H) Co-staining for Id1 and Oct4 in E5.5 mouse embryo. Id1 is co-expressed with Oct4 in a subset of cells in the proximal region of the epiblast, and is expressed in the visceral endoderm.

(I) qRT-PCR for *Id1* in 2i+LIF to EpiLC differentiation of E14Ju09 ES cells. Error bars: standard deviation of 3 biological replicates.

FIGURE S2 - Malaguti et al

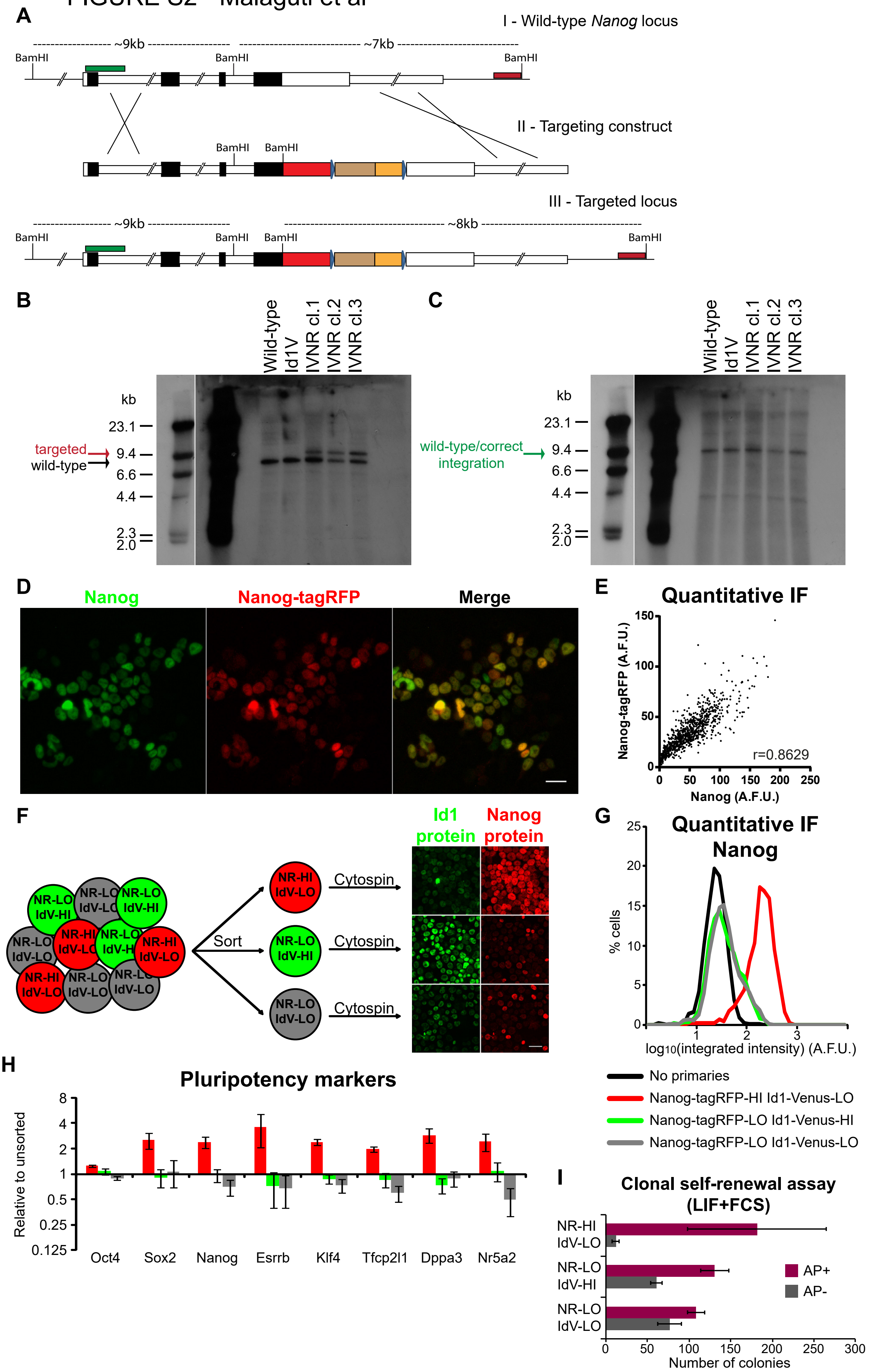

Figure S2. Characterisation of Id1-Venus Nanog-tagRFP reporter ES cells and their sorted subpopulations, Related to Figure 2.

(A) Strategy to target the Nanog locus with a *Nanog-tagRFP* fusion construct. Black boxes: exonic DNA, large white boxes: UTRs, medium white boxes: intronic DNA, single black line: intergenic DNA, large red box: *tagRFP*, brown box: *IRES*, yellow box: *Bls* resistance gene, blue triangles: *loxP* sites, medium red box: Southern blot targeting probe, medium green box: Southern blot multiple integration probe. Sizes of expected bands following BamHI restriction digest are indicated.

(B) Southern blot with targeting probe on BamHI-digested genomic DNA extracted from wild-type ES cells, Id1-Venus parental cells (Id1V) and three different Id1-Venus Nanog-tagRFP (IVNR) clones. All three clones present the expected banding pattern for correctly targeted clones. A lower exposure of the λ DNA-HindIII digest ladder is included to the left of the blot.

(C) Southern blot with multiple integration probe on BamHI-digested genomic DNA extracted from wild-type ES cells, Id1-Venus parental cells (Id1V) and three different Id1-Venus Nanog-tagRFP (IVNR) clones. All three clones present the expected banding pattern for correctly targeted clones. A lower exposure of the λ DNA-HindIII digest ladder is included to the left of the blot.

(D) Co-staining for Nanog and tagRFP in IVNR cells cultured in LIF+FCS. Scale bar: 30μm.

(E) Immunofluorescence quantification of the cells in (D) show high correlation between Nanog and tagRFP expression (Pearson's r=0.8629). A.F.U.: arbitrary fluorescence units.

(F) Sorting of IVNR ES cells cultured in LIF+FCS into 3 subpopulations based on Id1-Venus and Nanog-tagRFP expression: Nanog-tagRFP-high Id1-Venus-low (NR-HI IdV-LO), Nanog-tagRFP-low Id1-Venus-high (NR-LO IdV-HI) and Nanog-tagRFP-low Id1-Venus-low (NR-LO IdV-LO). Cytospin of the three subpopulations was performed immediately after sorting, and cells were stained for Id1 and Nanog expression. Scale bar: 30μm.

(G) Immunostaining quantification of Nanog expression in the three sorted subpopulations in (F) reveals the presence of similar low numbers of Nanog-medium cells in the two NR-LO subpopulations.

(H) qRT-PCR analysis of the sorted samples in (F) reveal similar levels of pluripotency marker expression between the two NR-LO subpopulations, with the exception of *Nr5a2*, which is higher in NR-LO IdV-HI than NR-LO IdV-LO ES cells. Data are represented as mean ± SEM of seven independent experiments.

(I) Clonal self-renewal assays for cells sorted in (F) reveal no significant differences in alkaline phosphatase-positive colony-forming ability between NR-LO IdV-HI and NR-LO IdV-LO cells. 300 cells were plated on $10cm^2$ for each sample. Data are represented as mean ± SEM of three independent experiments.

FIGURE S3

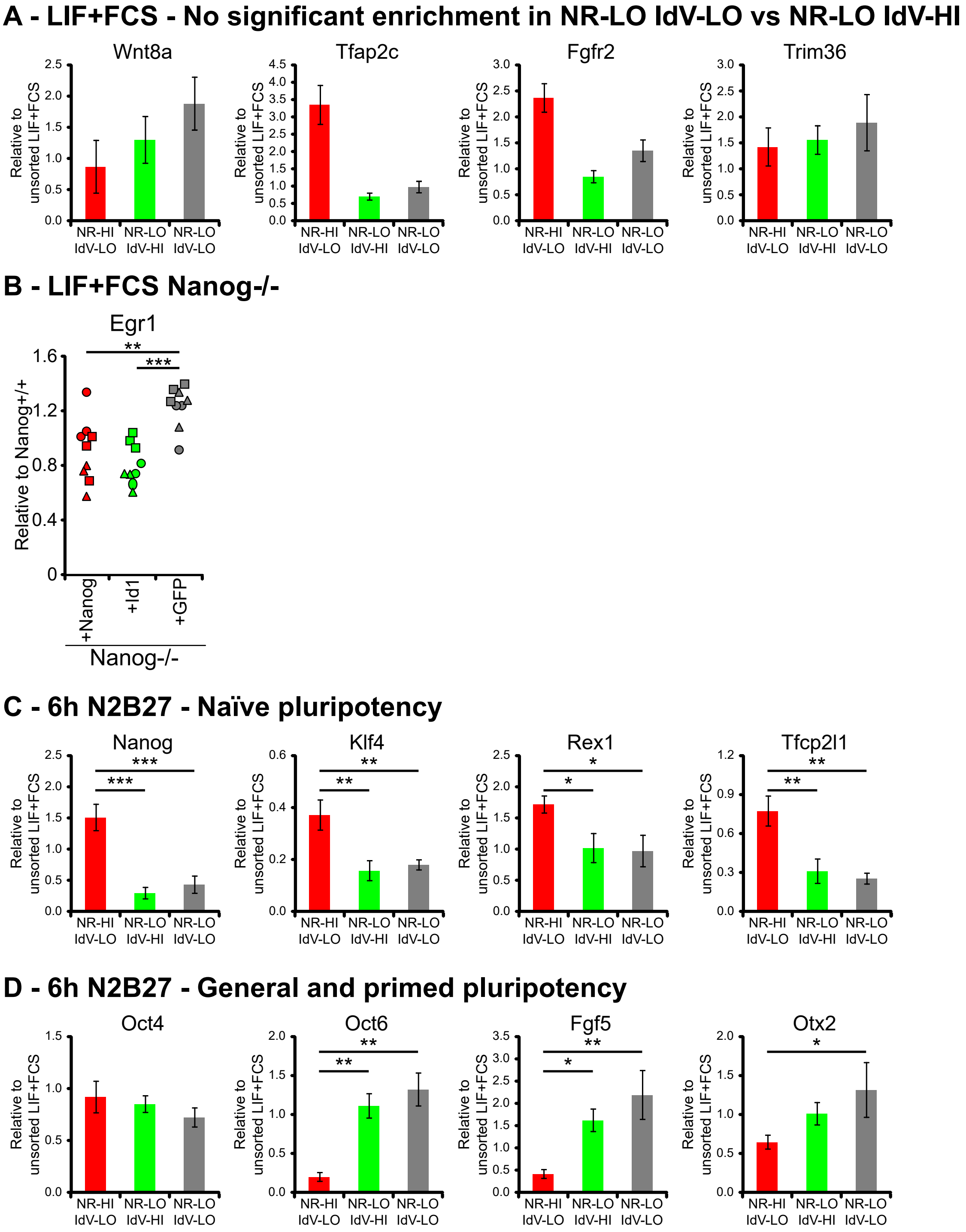
A - LIF+FCS - No significant enrichment in NR-LO IdV-LO vs NR-LO IdV-HI
Wnt8a
Tfap2c
Fgfr2
Trim36
Relative to unsorted LIF+FCS
NR-HI IdV-LO
NR-LO IdV-HI
NR-LO IdV-LO
B - LIF+FCS Nanog-/-
Egr1
Relative to Nanog+/+
+Nanog
+Id1
+GFP
Nanog-/-
C - 6h N2B27 - Naïve pluripotency
Nanog
Klf4
Rex1
Tfcp2l1
D - 6h N2B27 - General and primed pluripotency
Oct4
Oct6
Fgf5
Otx2

Figure S3. Comparison of Nanog-low Id1-low and Nanog-low Id1-high cells, Related to Figure 3.

(A) qRT-PCR experiments showing lack of statistically significant (one-way ANOVA followed by unpaired t-test, $p<0.05$) differential expression between NR-LO IdV-LO and NR-LO IdV-HI subpopulations for transcripts in Figure 3B. Data are presented as mean ± SEM of seven independent experiments. Validation of transcripts that showed statistically significant expression (*Egr1*, *Lefty1*) is shown in Figure 3C.

(B) qRT-PCR analysis for *Egr1* of Nanog-rescue cells cultured in LIF+FCS. Each shape represents a different clonal line.

(C) qRT-PCR analysis of naïve pluripotency marker expression in sorted subpopulations after the 6h N2B27 differentiation challenge.

(D) qRT-PCR analysis of general and primed pluripotency marker expression in sorted subpopulations after the 6h N2B27 differentiation challenge.

Data in (C) and (D) are represented as mean ± SEM of three independent experiments. Statistical analyses (unless specified): one-way ANOVA followed by Tukey's multiple comparison test. *$p<0.05$, **$p<0.01$, ***$p<0.001$.

FIGURE S4

A - Transcriptome data

Transcipts enriched in NR-LO IdV-HI vs NR-LO IdV-LO

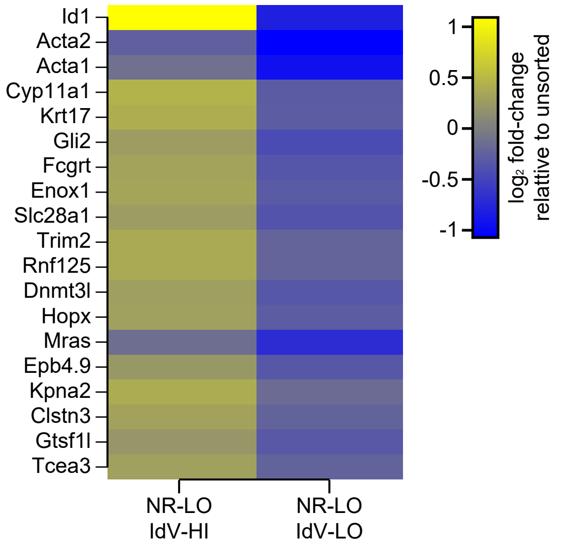

B - qRT-PCR - Significant enrichment in NR-LO IdV-HI vs NR-LO IdV-LO LIF+FCS

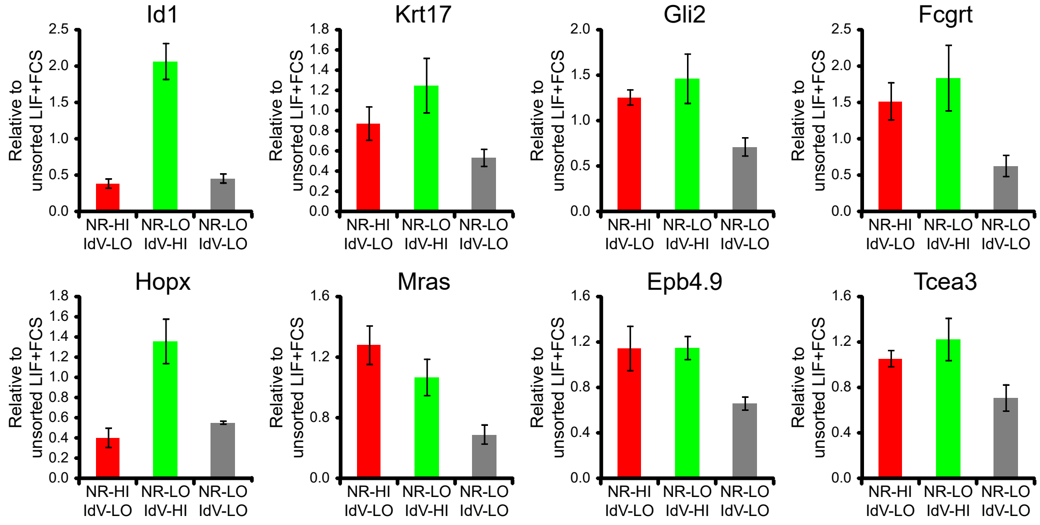

C - qRT-PCR - No significant enrichment in NR-LO IdV-HI vs NR-LO IdV-LO LIF+FCS

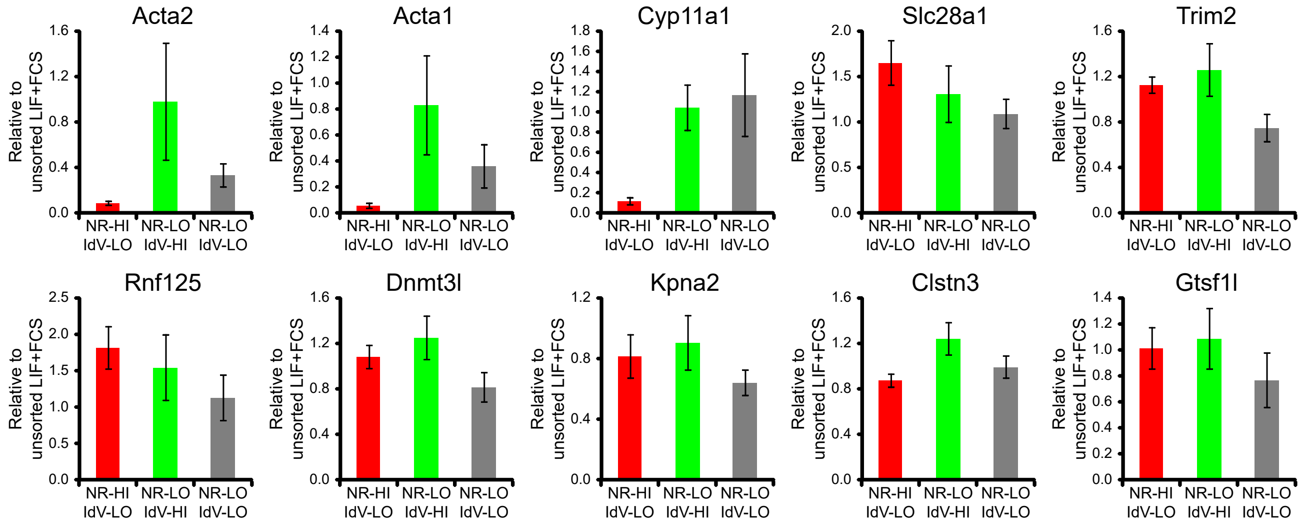

Figure S4. Transcripts enriched in Nanog-low Id1-high vs Nanog-low Id1-low cells, Related to Figure 4.

(A) Heatmap of transcripts significantly enriched in NR-LO IdV-HI relative to NR-LO IdV-LO subpopulations. Significance was defined as $\log_2$(fold-change)>0.5, p-value adjusted for multiple testing correction <0.5. Methods for statistical analysis of transcriptomic data are described in the STAR Methods section.

(B) qRT-PCR experiments showing statistically significant (ANOVA followed by unpaired t-test, $p<0.05$) differential expression between NR-LO IdV-HI and NR-LO IdV-LO subpopulations for transcripts in (A).

(C) qRT-PCR experiments showing lack of statistically significant (ANOVA followed by unpaired t-test, $p<0.05$) differential expression between NR-LO IdV-HI and NR-LO IdV-LO subpopulations for transcripts in (A).

qRT-PCR data are represented as mean ± SEM of seven independent experiments.

FIGURE S5

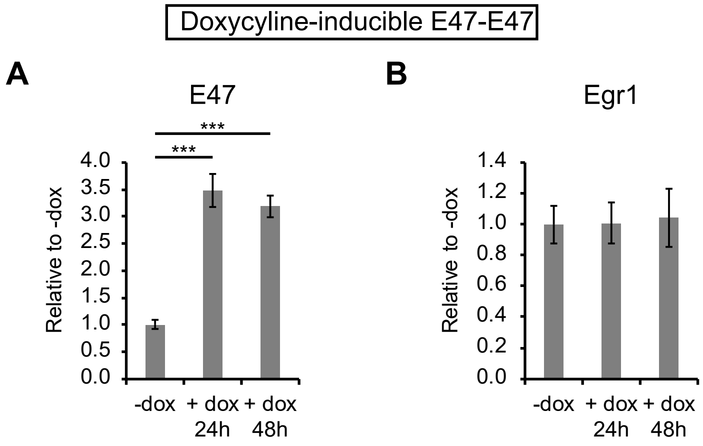

Figure S5. E47 homodimers do not induce Egr1 expression in ES cells, Related to Figure 5.

Mouse ES cells harbouring a doxycycline-inducible 3xFlag-E47-E47 transgene were cultured in LIF+FCS for 48h in the absence ("-dox") or the presence ("+dox") of 1µg/ml doxycycline (dox) for 24h or 48h. qRT-PCRs were performed to assess the expression of (A) *E47* and (B) *Egr1*. Data are presented as mean ± SEM of six independent experiments.

FIGURE S6

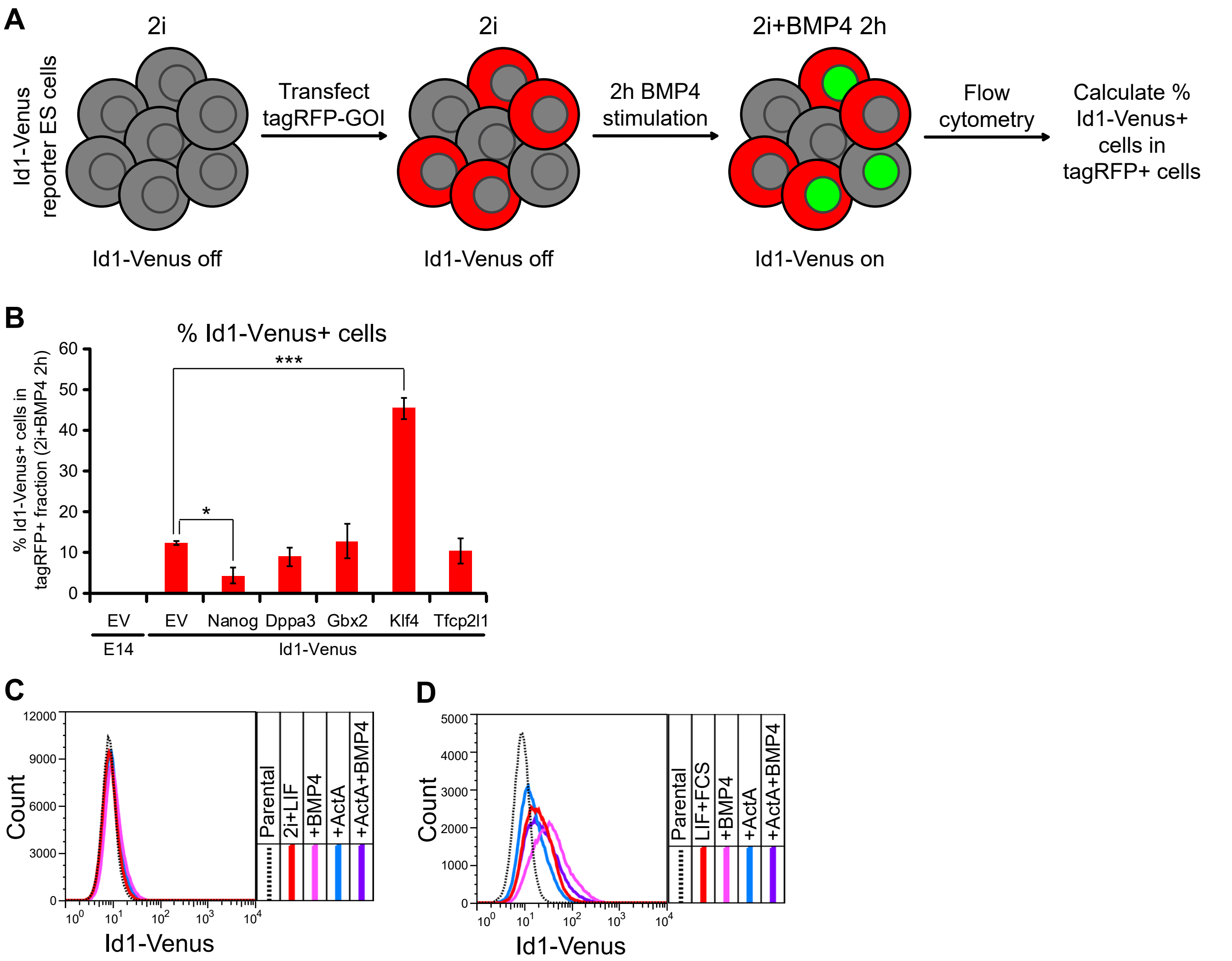

Figure S6. Nanog and Activin A repress Id1-Venus expression, Related to Figure 6.

(A) Experimental strategy. Id1-Venus reporter ES cells were cultured in 2i, a condition in which Id1-Venus is not expressed. Overexpression plasmids encoding tagRFP fused to genes of interest were lipofected into the cultures. After 48h, the cells were treated with 10ng/ml BMP4 for 2h to enable Id1-Venus expression (unlike 2i+LIF, Id1 is expressed in response to BMP4 in 2i), then subject to flow cytometry analysis. The percentage of cells upregulating Id1-Venus within the tagRFP-positive compartment was calculated.

(B) Quantification of the experiment described in (A). Five pluripotency transcription factors were tested, and of these only Nanog was able to repress Id1-Venus expression. EV: tagRFP-empty vector.

Data are presented as mean ± SEM of three independent experiments. Statistical analyses were performed using a one-way ANOVA, followed by Dunnett's multiple comparison test of all genes of interest with the empty vector control. *$p<0.05$, ***$p<0.001$.

(C) Flow cytometry analysis of Id1-Venus ES cells cultured in 2i+LIF with or without 48h stimulation with 10ng/ml BMP4 and/or 20ng/ml Activin A.

(D) Flow cytometry analysis of Id1-Venus ES cells cultured in LIF+FCS with or without 48h stimulation with 10ng/ml BMP4 and/or 20ng/ml Activin A.

FIGURE S7

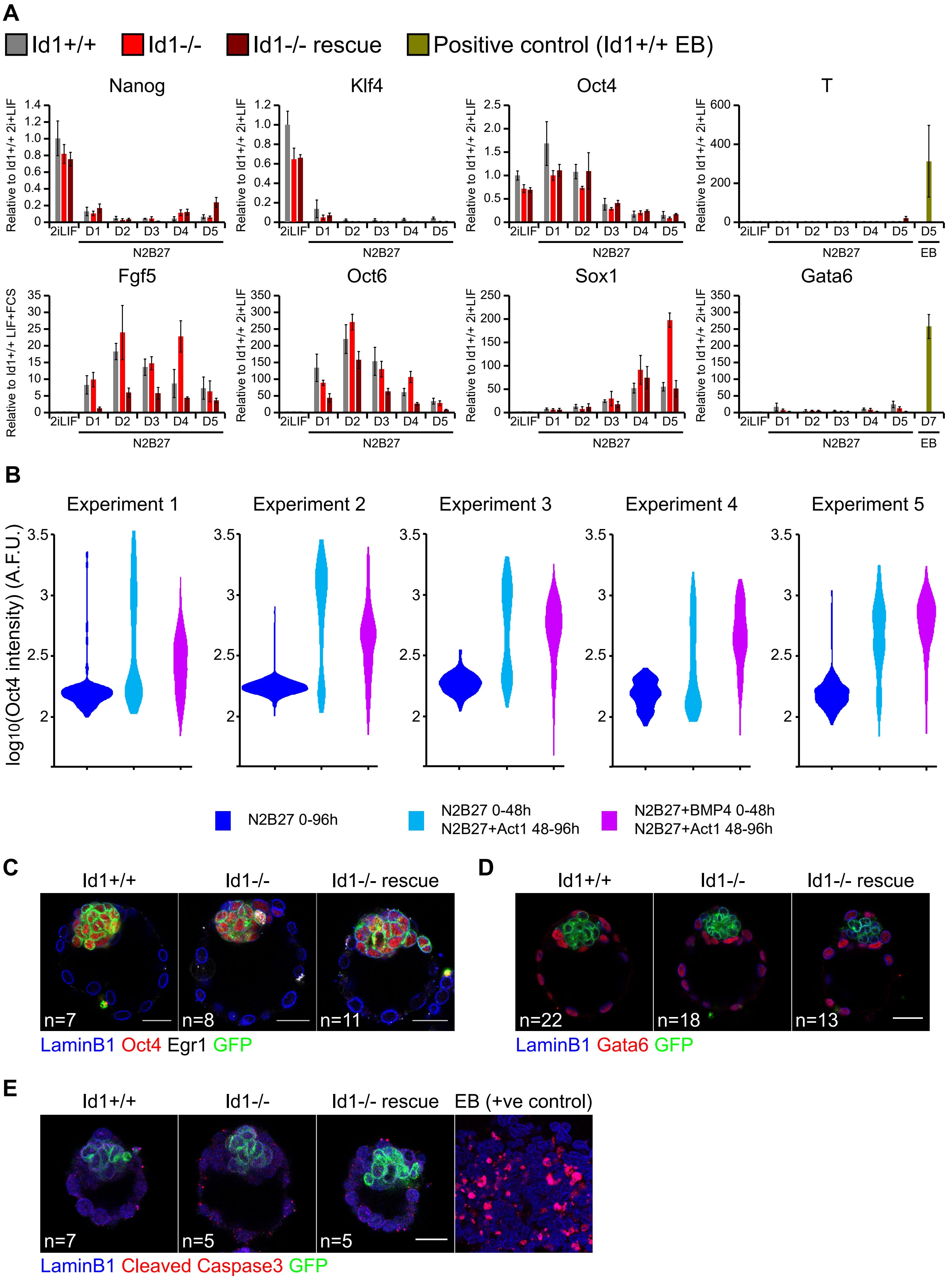
A
Id1+/+
Id1-/-
Id1-/- rescue
Positive control (Id1+/+ EB)
Nanog
Klf4
Oct4
T
Relative to Id1+/+ 2i+LIF
2iLIF
D1
D2
D3
D4
D5
N2B27
EB
Fgf5
Oct6
Sox1
Gata6
Relative to Id1+/+ LIF+FCS
D7
B
Experiment 1
Experiment 2
Experiment 3
Experiment 4
Experiment 5
log10(Oct4 intensity) (A.F.U.)
N2B27 0-96h
N2B27 0-48h
N2B27+Act1 48-96h
N2B27+BMP4 0-48h
N2B27+Act1 48-96h
C
Id1+/+
Id1-/-
Id1-/- rescue
n=7
n=8
n=11
LaminB1 Oct4 Egr1 GFP
D
n=22
n=18
n=13
LaminB1 Gata6 GFP
E
EB (+ve control)
n=7
n=5
n=5
LaminB1 Cleaved Caspase3 GFP

Fig S7. Id1 enables a robust transition from naïve to primed pluripotency in the face of conflicting signals, Related to Figure 7.

(A) qRT-PCR of wild-type, Id1-null and Id1-rescue ES cells undergoing 2i+LIF --> N2B27 differentiation. All cell lines are able to exit pluripotency, with Id1-null cells displaying a pro-neural phenotype. Positive control samples from wild-type embryoid bodies (EB) are included for *T* and *Gata6*. Data are presented as mean ± standard deviation of at least three biological replicates.

(B) Distribution of Oct4 expression in 5 replicate experiments described in Figure 7A-D. Experiment 3 is also shown in Figure 7C. Act1: 1ng/ml Activin A. A.F.U.: Arbitrary fluorescence units.

(C) Representative images of Id1+/+, Id1-/- and Id1-/- rescue chimaeras, cultured to the blastocyst stage and stained for LaminB1, GFP, Oct4 and Egr1. Egr1 expression was detected in a single cell of an Id1-/- chimaera (shown in Figure) across all stained blastocysts. Scale bars: 30μm.

(D) Representative images of Id1+/+, Id1-/- and Id1-/- rescue chimaeras, cultured to the blastocyst stage and stained for LaminB1, GFP and Gata6. Scale bar: 30μm.

(E) Representative images of Id1+/+, Id1-/- and Id1-/- rescue chimaeras, cultured to the blastocyst stage and stained for LaminB1, GFP and Cleaved Caspase3. An embryoid body (EB) section is included as a positive control. Scale bar: 30μm.

Table S2. List of primers used in this study, Related to STAR Methods.

qRT-PCR primer sequences		
**Description**	**Sequence**	**UPL Probe #**
Acta1 Forward	TGAAGCCTCACTTCCTACCC	81
Acta1 Reverse	CGTCGCACATGGTGTCTAGT	81
Acta2 Forward	CTCTCTTCCAGCCATCTTTCAT	58
Acta2 Reverse	TATAGGTGGTTTCGTGGATGC	58
Clstn3 Forward	TTGAGAGTGCTCGTCCTGTC	6
Clstn3 Reverse	GCCATTCATCATCACACACC	6
Cyp11a1 Forward	AAGTATGGCCCCATTTACAGG	104
Cyp11a1 Reverse	TGGGGTCCACGATGTAAACT	104
Dnmt3l Forward	AACCGACGGAGCATTGAA	34
Dnmt3l Reverse	AAACAAGGGGTGCCGAGT	34
Dppa3 Forward	GATAGGATGCACAACGATCCA	73
Dppa3 Reverse	AAAAATGCTTTTATTACAAATTTCTGG	73
E47 Forward	GTGGGCTCTGACAAGGAACT	79
E47 Reverse	ACAGGTAGCGGGAACATCAT	79
Epb4.9 Forward	TCATGATCTATGAGCCCCACT	79
Epb4.9 Reverse	GTGGATTTGGGTGACAGTGA	79
Egr1 Forward	CCTATGAGCACCTGACCACA	22
Egr1 Reverse	TCGTTTGGCTGGGATAACTC	22
Egr2 Forward	CTACCCGGTGGAAGACCTC	60
Egr2 Reverse	GTCAATGTTGATCATGCCATCT	60
Esrrb Forward	CGATTCATGAAATGCCTCAA	89
Esrrb Reverse	CCTCCTCGAACTCGGTCA	89
Fcgrt Forward	CCTCAAGACCCTGGAGAAGAT	40
Fcgrt Reverse	TATCCGAGGCCAGTTCACA	40
Fgf5 Forward	AAAACCTGGTGCACCCTAGA	29
Fgf5 Reverse	CATCACATTCCCGAATTAAGC	29
Fgfr2 Forward	CCTGCGGAGACAGGTAACAG	17
Fgfr2 Reverse	CGGGGTGTTGGAGTTCAT	17
Gata4 Forward	CCCCTCATTAAGCCTCAGC	77
Gata4 Reverse	CACCCTCGGCATTACGAC	77
Gata6 Forward	GGTCTCTACAGCAAGATGAATGG	40
Gata6 Reverse	TGGCACAGGACAGTCCAAG	40
Gli2 Forward	CCAGTGGCTCTTATGGACATC	26
Gli2 Reverse	GTAGGCCACAGGATTGATGG	26
Gtsf1l Forward	GGCTTTTCCTTTCGAGGTGT	18
Gtsf1l Reverse	TATGGATTCTGGCTCCATCC	18
Hopx Forward	ACCACGCTGTGCCTCATC	68
Hopx Reverse	GCGCTGCTTAAACCATTTCT	68
Id1 Forward	TCCTGCAGCATGTAATCGAC	78
Id1 Reverse	GGTCCCGACTTCAGACTCC	78

Klf4 Forward	CGGGAAGGGAGAAGACACT	62
Klf4 Reverse	GAGTTCCTCACGCCAACG	62
Kpna2 Forward	CTGCCCGACTTAACAGGTTC	56
Kpna2 Reverse	TTAGCTTTCCTGAGTTCCACATT	56
Krt17 Forward	AGGAGCTGGCCTACCTGAA	63
Krt17 Reverse	ACCTGGCCTCTCAGAGCAT	63
Lefty1 Forward	CTCAGTATGTGGCCCTGCTAC	76
Lefty1 Reverse	AACCTGCCTGCCACCTCT	76
Lefty2 Forward	GCCCTCATCGACTCTAGGC	97
Lefty2 Reverse	AGCTGCTGCCAGAAGTTCAC	97
Mras Forward	TGGGCCATCTTGGATGTT	51
Mras Reverse	CTGTGCGCATGTATTGTTCC	51
Nanog Forward	CCTCCAGCAGATGCAAGAA	25
Nanog Reverse	GCTTGCACTTCATCCTTTGG	25
Nr5a2 Forward	ATGGGAAGGAAGGGACAATC	22
Nr5a2 Reverse	TTGTTGAACGCGACTTCTGT	22
Oct4 Forward	GTTGGAGAAGGTGGAACCAA	95
Oct4 Reverse	CTCCTTCTGCAGGGCTTTC	95
Oct6 Forward	CTCAAGCCGCTGCTCAAC	25
Oct6 Reverse	CGCGATCTTGTCCAGGTT	25
Otx2 Forward	GACCCGGTACCCAGACATC	103
Otx2 Reverse	GCTCTTCGATTCTTAAACCATACC	103
Rex1 Forward	CAGCTCCTGCACACAGAAGA	16
Rex1 Reverse	ACTGATCCGCAAACACCTG	16
Rnf125 Forward	GAGGACTGAGATTTAAGGTGGTG	103
Rnf125 Reverse	GCAAGATCGGCAGAACCA	103
Sdha Forward	CAGTTCCACCCCACAGGTA	71
Sdha Reverse	TCTCCACGACACCCTTCTGT	71
Slc28a1 Forward	GCTGTTTGAGTGGATCAGCA	108
Slc28a1 Reverse	GACGATCAAGAAGCCGATGT	108
Sox1 Forward	GTGACATCTGCCCCCATC	60
Sox1 Reverse	GAGGCCAGTCTGGTGTCAG	60
Sox2 Forward	GTGTTTGCAAAAAGGGAAAAGT	34
Sox2 Reverse	TCTTTCTCCCAGCCCTAGTCT	34
Sox17 Forward	CACAACGCAGAGCTAAGCAA	97
Sox17 Reverse	CGCTTCTCTGCCAAGGTC	97
T Forward	ACTGGTCTAGCCTCGGAGTG	27
T Reverse	TTGCTCACAGACCAGAGACTG	27
Tbp Forward	GGGGAGCTGTGATGTGAAGT	97
Tbp Reverse	CCAGGAAATAATTCTGGCTCA	97
Tcea3 Forward	AGCTGAACAGTTGCCAGATG	42
Tcea3 Reverse	ACTGCCACTCCGATCCTG	42
Tcf15 Forward	GTGTAAGGACCGGAGGACAA	104
Tcf15 Reverse	GATGGCTAGATGGGTCCTTG	104
Tfcp2l1 Forward	GGGGACTACTCGGAGCATCT	53

Tfcp2l1 Reverse	TTCCGATCAGCTCCCTTG	53
Tfap2c Forward	CGCGGAAGAGTATGTTGTTG	62
Tfap2c Reverse	CGATCTTGATGGAGAAGGTCA	62
Trim2 Forward	GTGGACTCAAATGGGAACATC	5
Trim2 Reverse	TGATCCACTCCCATCAAAAAC	5
Trim36 Forward	CACCTCCCCTAGCATGGATA	64
Trim36 Reverse	TCGGGGTCAGTGAATTACG	64
Wnt8a Forward	ACTGCGGCTGTGACGAGT	75
Wnt8a Reverse	CCCGAACTCCACGTTGTC	75
Ywhaz Forward	TTACTTGGCCGAGGTTGCT	9
Ywhaz Reverse	TGCTGTGACTGGTCCACAAT	9

Southern blot probe amplification primer sequences

**Description**	**Sequence**
Nanog targeting probe forward	AAATTTATTTCTGGGCATGTGGTGAC
Nanog targeting probe reverse	GGATCCAGATTTCAGGATTGGAGG
Nanog multiple integration probe forward	TTCAGAAATCCCTTCCCTCG
Nanog multiple integration probe reverse	ATGCTAACTGCTTCTGCTGG
